# Triantennary GalNAc Molecular Imaging Probes for Monitoring Hepatocyte Function in a Rat Model of Nonalcoholic Steatohepatitis

**DOI:** 10.1002/advs.202002997

**Published:** 2020-11-09

**Authors:** Anurag Mishra, Tamara R. Castañeda, Erik Bader, Bettina Elshorst, Sheila Cummings, Petra Scherer, Dinesh S. Bangari, Claudia Loewe, Herman Schreuder, Christoph Pöverlein, Mike Helms, Seth Jones, Gernot Zech, Thomas Licher, Michael Wagner, Manfred Schudok, Meltsje de Hoop, Alleyn T. Plowright, Jens Atzrodt, Aimo Kannt, Iina Laitinen, Volker Derdau

**Affiliations:** ^1^ Industriepark Höchst 65926 Frankfurt Germany; ^2^ R&D Diabetes Sanofi‐Aventis Deutschland GmbH Industriepark Höchst 65926 Frankfurt Germany; ^3^ Global Discovery Pathology Translational In Vivo Models Sanofi Genzyme The Mountain Road Framingham MA 01701 USA; ^4^ Global Bioimaging Translational In Vivo Models Sanofi‐Aventis Deutschland GmbH Industriepark Höchst 65926 Frankfurt Germany; ^5^ R&D Drug Metabolism and Pharmacokinetics Sanofi‐Aventis Deutschland GmbH Industriepark Höchst 65926 Frankfurt Germany; ^6^ Wren Therapeutics Ltd. Department of Chemistry University of Cambridge Lensfield Rd Cambridge CB2 1EW UK; ^7^ R&D Transversal Operations German R&D Hub Sanofi‐Aventis Deutschland GmbH Industriepark Höchst 65926 Frankfurt Germany; ^8^ Experimental Pharmacology Medical Faculty Mannheim University of Heidelberg 68167 Mannheim Germany; ^9^ Fraunhofer IME Translational Medicine and Pharmacology 60596 Frankfurt Germany; ^10^ Present address: Antaros Medical, Bioventure Hub Mölndal 431 83 Sweden

**Keywords:** asialoglycoprotein receptor, *β*‐d‐galactose or *N*‐acetylgalactosamine, insulin resistance, nonalcoholic steatohepatitis, obesity, positron emission tomography imaging, Zucker fatty/spontaneously hypertensive heart failure F1 hybrid rats

## Abstract

Nonalcoholic steatohepatitis (NASH) is a progressive form of nonalcoholic fatty liver disease that can lead to irreversible liver cirrhosis and cancer. Early diagnosis of NASH is vital to detect disease before it becomes life‐threatening, yet noninvasively differentiating NASH from simple steatosis is challenging. Herein, bifunctional probes have been developed that target the hepatocyte‐specific asialoglycoprotein receptor (ASGPR), the expression of which decreases during NASH progression. The results show that the probes allow longitudinal, noninvasive monitoring of ASGPR levels by positron emission tomography in the newly developed rat model of NASH. The probes open new possibilities for research into early diagnosis of NASH and development of drugs to slow or reverse its progression.

## Graphical abstract

New multifunctional positron emission tomography (PET) probes are shown that target the hepatocyte‐specific asialoglycoprotein receptor, the expression of which decreases during nonalcoholic steatohepatitis progression. The probes allow longitudinal, noninvasive monitoring of receptor levels by PET in the newly developed rat model allowing to study the progress of nonalcoholic steatohepatitis progression without biopsy.

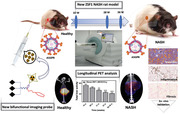

## 1 Introduction

Nonalcoholic fatty liver disease, the leading cause of chronic liver disease and liver transplantation, is characterized by progressive accumulation of lipids within the liver parenchyma, leading to inflammation, hepatocyte loss, and fibrogenesis. The global prevalence of nonalcoholic fatty liver disease is estimated to be around 25% in the general population, 66% among individuals with type 2 diabetes and 90% among obese individuals, and this prevalence may increase up to 2–3‐fold by 2030.^[^ ^1^, ^2^, ^3^, ^4^, ^5^, ^6^, ^7^ ^]^ As the disease progresses, simple steatosis and nonalcoholic steatohepatitis (NASH) can develop into life‐threatening cirrhosis and hepatocellular cancer.^[^ ^8^ ^]^

Early diagnosis and fibrosis staging of NASH would help clinicians stratify patients according to risk of progression, allowing them to focus monitoring, management and treatment accordingly. The current gold standard for diagnosing and staging NASH is invasive liver biopsy, which poses risks for patients, and cannot be performed repeatedly during longitudinal assessment of treatment response. Furthermore, it may lead to inaccurate results because of heterogeneity in the liver tissue and subjective interpretation.^[^ ^6^, ^9^, ^10^, ^11^ ^]^ Noninvasive ultrasonography and magnetic resonance imaging can diagnose advanced fibrosis, but they cannot accurately differentiate NASH from other forms of nonalcoholic fatty liver disease.^[^ ^9^ ^]^ Similarly, blood‐based biomarkers, which can easily be assayed noninvasively, cannot reliably distinguish different NASH stages.^[^ ^2^ ^]^

The lack of NASH‐specific biomarkers that can be monitored noninvasively hinders not only early recognition of the disease but also the development of effective treatments. Another hindrance is the lack of an animal model that recapitulates the complete NASH phenotype, including obesity and impaired glucose metabolism.^[^ ^12^, ^13^, ^14^, ^15^, ^16^, ^17^, ^18^ ^]^ We overcome both challenges in the present study by developing imaging probes that selectively bind hepatocyte‐specific asialoglycoprotein receptor (ASGPR), whose expression falls during development of liver fibrosis^[^ ^19^, ^20^ ^]^ and inflammatory processes,^[^ ^21^ ^]^ and by creating a new rat model of NASH progression that recapitulates the obesity and insulin resistance of the disease, in which ASGPR can be monitored in vivo using our imaging probes in combination with positron emission tomography (PET).

ASGPR can be a potential biomarker for monitoring NASH progression in our rat model via PET imaging. To date, ASGPR has not been proven to monitor NASH progression by any imaging modality. ASGPR, expressed exclusively on hepatocytes, normally binds nonreducing terminal *β*‐d‐galactose (d‐Gal) or *N*‐acetylgalactosamine (GalNAc) residues with high affinity.^[^ ^22^ ^]^ So far, the visualization of liver fibrosis progression by quantification of ASGPR levels with a ^18^F‐labeled monovalent galactose derivative via single‐photon emission computed tomography has been reported in a single study.^[^ ^23^ ^]^ However, high concentrations of the tracer in the µM range were necessary due to the low affinity of the tracer to the receptor. Another approach to study liver fibrosis was accomplished utilizing ^99m^Tc‐ mono GalNAc‐conjugated polyethylenimine.^[^ ^24^ ^]^ In this study part of the tracer was unselectively filtered in the liver due to the increased size of the polymer as could be observed in the control experiment. Other described ASGPR‐binding probes for PET imaging of further liver diseases rely on *β*‐d‐galactose (d‐Gal)^[^ ^25^, ^26^, ^27^ ^]^ to increase affinity for ASGPR and these probes were conjugated to human serum albumin (HSA) to prolong half‐life in blood circulation.^[^ ^18^, ^20^, ^26^, ^27^ ^]^ A few other approaches to use molecular tracers in nonalcoholic fatty liver (NAFLD) imaging using tryptophan‐rich sensory protein, Integrin *α_v_β*_3_ or GSA receptors have been attempted but the animal mouse models used in these studies were not clinically translatable to humans.^[^ ^28^ ^]^

We describe herein a method to overcome the above‐mentioned specificity problems by developing a series of bifunctional chemical imaging probes that selectively bind to hepatocyte specific ASGPR and to study the receptor decrease during development of liver fibrosis in a new rat model of NASH by PET imaging and fluorescence tomography. Using these “first‐in‐class” chemical imaging probes, we followed the progression from steatosis to fibrosis and thereby establish a new paradigm in noninvasive monitoring of hepatocyte function immediately relevant for a broad range of liver diseases.

## 2 Results and Discussion

### 2.1 Design and Synthesis of GalNAc‐NASH (GN) Imaging Probes

In our study, we used GalNAc, which as a monomer binds to the receptor with 10‐ to 50‐fold higher affinity than d‐Gal.^[^ ^25^ ^]^ The receptor binds triantennary GalNAc with much higher affinity than monomeric GalNAc^[^ ^29^, ^30^ ^]^ and the triantennary motif has been used previously for liver‐specific delivery of short interfering RNAs^[^ ^31^ ^]^ and nanoparticles.^[^ ^32^, ^33^, ^34^, ^35^ ^]^ We engineered bifunctional GN probes that combined triantennary GalNAc with ^68^Gallium (^68^Ga) labeled dodecane tetraacetic acid (DOTA) to allow PET imaging and/or with heptamethine cyanine dye (Cy 5.5) to allow near‐infrared optical imaging. The radioisotope, ^68^Ga, has a half‐life of 68 min, and it is strongly chelated by a macrocyclic chelator such as DOTA.

**Figure 1:**
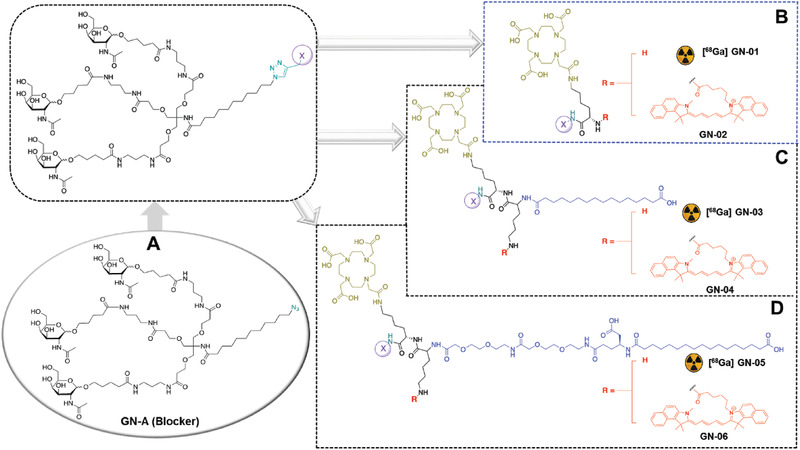
Structures of GN probes. A) All GN probes were derived from the parental GN‐A. B) GN‐01 and GN‐02 lacked moieties to interact with proteins. C) GN‐03 and GN‐04 contained such moieties, connected directly to the main backbone using palmitic acid. D) GN‐05 and GN‐06 contained serum protein‐binding moieties connected to the main backbone via an extended linker of palmitic acid and PEG.

In order to evaluate whether a prolonged half‐life of the chemical imaging probe was required in blood circulation, we developed a series of GN probes. The chemical probes GN‐01 and GN‐02 are the most straightforward and contain DOTA, triantennary GalNAc and/or Cy5.5 (**Figure** **1**B). Chemical probes GN‐03 to GN‐06 also contain palmitic acid to interact with albumin,^[^ ^36^ ^]^ to increase their half‐life in circulation and therefore allowing the probes to persist longer in the bloodstream. The palmitic acid is connected directly to the rest of the molecule in GN‐03 and GN‐04 (Figure 1C) or via an extended PEGylated linker in GN‐05 and GN‐06 (Figure 1D). GN‐02/‐04/‐06 are dye conjugated molecules which correspond to the parent [^68^Ga] GN‐01/‐03/‐05, respectively and these probes were further used to study the receptor binding behavior by optical imaging.

All GN probes were synthesized in multiple synthetic steps via connecting different building blocks involving glycosylation, tosylation, alkyl azidation, amidation, protection, deprotection, and enzymatic hydrolysis (Schemes S1–S5, Supporting Information). These building blocks were coupled to the intermediate triantennary GalNAc azide unit (GN‐A) (Figure 1A) via a copper‐catalyzed azide‐alkyne cycloaddition reaction. All intermediate and final molecules were extensively characterized spectroscopically and chromatographically and obtained in excellent purity (>95%) (for details see the Supporting Information).

### 2.2 Radiochemical Purity and Plasma Stability

For PET imaging applications, ^68^Ga labeling on GN probes (GN‐01/03/05) were performed using a commercial automated Scintomics GRP system (for details see the Supporting Information). [^68^Ga] GN probes were prepared in > 94% radiochemical purity (Figure S1, Supporting Information). The chemical probes were chemically stable in buffer formulation at ambient temperature for over 4 h. Furthermore, after incubation in Sprague–Dawley (SD) rat plasma the GN probes were determined to be sufficiently stable (≥92%) for up to 1 h. No degradation was observed up to 1 h incubation with HSA (Figure S2, Supporting Information).

### 2.3 Structural Studies of GN Probes Binding to ASGPR

**Figure 2:**
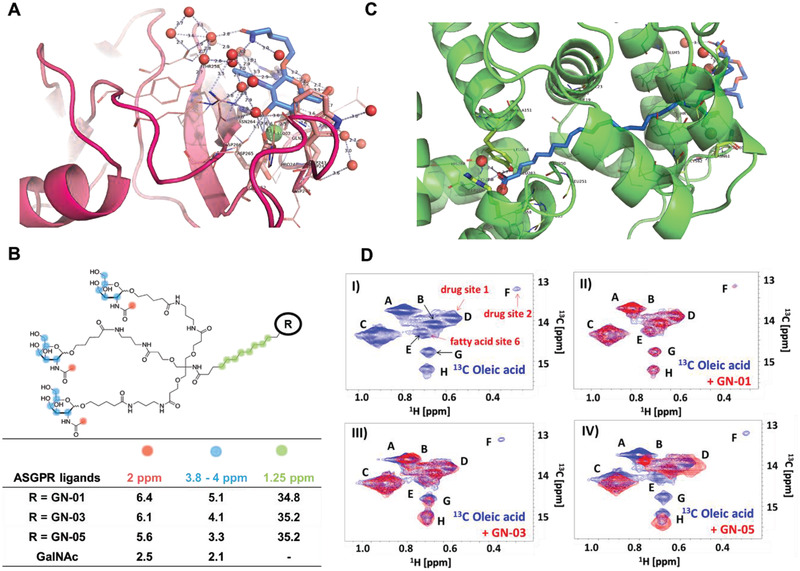
Structural analysis of GN probes. A) Crystal structure of GN‐A (blue) bound to the H1 domain of ASGPR (scarlet), showing interaction of the trimeric GalNAc to the protein. Electron density of the omit map is shown at a 2*σ* contour. Bound calcium ions are depicted as green spheres; water molecules, as red spheres. Close‐up look of the GN‐A binding site, in which the GalNAC interacts with a calcium ion (green sphere); the side chains of Arg236, Trp234, and Asn264; and water molecules (red spheres). Full crystal structure is displayed in supplementary file. B) STD NMR measurements of GN probes with ASGPR. Binding of compounds GN‐01, GN‐03, GN‐05, and monomeric GalNAc to ASGPR was quantified by the STD scaling factor for three groups of separated signals: the methyl protons of the acetyl group at 2 ppm (red), the protons of the sugar ring (blue) and the CH_2_ groups of the linker (green). C) Crystal structure of GN‐07 (blue) bound to HSA (green), showing interactions between the terminal fatty acid moiety of the probe and the protein. Electron density of the omit map is shown at a 2*σ* contour. Close‐up look of the terminal fatty acid moiety of the GN‐07 binding site. Full crystal structure is displayed in supplementary file. Zoomed crystal structure shows the binding site of the terminal carboxylate group (red) of the fatty acid, on the right is where the linker exits the binding site. Water molecules are shown as red spheres. D) Competition experiment between GN probes and ^13^C‐labeled oleic acid for binding to HSA. I) ^1^H,^13^C‐HSQC NMR reference spectrum of excess ^13^C‐labeled oleic acid (2 × 10^−3^ m) bound to HSA (0.5 × 10^−3^ m) in phosphate buffer. The labeled peaks correspond to different binding sites within HSA. Binding sites labeled in red were assigned according to Krenzel et al.^[^ ^40^ ^]^ II–IV) ^1^H,^13^C‐HSQC NMR spectrum of the complex of ^13^C‐oleic acid and HSA (blue) overlaid with the spectrum (red) after addition of II) GN‐01; III) GN‐03, showing disappearance of peak F (drug site 2); or IV) GN‐05, showing a prominent shift of peak A and disappearance of peaks E (fatty acid site 6), F (drug site 2), and G.

To analyze how the probes, bind to the potential NASH biomarker, X‐ray crystallography and saturation transfer difference nuclear magnetic resonance (STD NMR) spectroscopic methods were used. The H1 domain of human ASGPR, which contains the GalNAc binding site,^[^ ^37^ ^]^ was crystallized and the crystals were then soaked in GN‐A solutions. The large solvent channels and open binding site allowed entry of the large trimeric GalNAc moiety within the preformed crystals (**Figure** **2**A). The 1.4 Å electron density maps (Figure 2A) showed unambiguous density not only for the GalNAc moiety, but also for the first 4–7 atoms of the linker. The density for the linker confirmed that the binding site was occupied by our chemical probe, rather than by the free monomeric GalNAc (control experiment) in the mother liquor (see the Supporting Information).

In the crystal structure, the 3‐OH and 4‐OH groups of the GalNAc moiety are bound to the calcium ion in ASGPR where the 3‐OH group is in close proximity to the calcium ligands Glu252 and Asn264, while the 4‐OH is very close to the calcium ligands Gln239 and Asp241. In addition, Arg236 makes bidentate hydrogen bonds with the oxygen of the acetyl group and interacts with the oxygen at the 1‐position of GalNAc, where the linker is attached. The nitrogen of the *N*‐acetyl group interacts with the side chain of Asn264. Finally, the 5‐hydroxymethyl group of GalNAc is stacked onto the indole ring system of Trp243. The linker itself does not appear to interact with ASGPR and is mostly disordered.

The binding of GN‐01, GN‐03, and GN‐05 to ASGPR was investigated using STD NMR,^[^ ^38^ ^]^ which detects the binding of different chemical moieties of the chemical probes to the protein (Figure 2B). STD scaling factors were measured, which reflect the intermolecular saturation transfer and therefore the contact of different protons of the bound probes to the protein.^[^ ^39^ ^]^ These factors were similar for all three probes, suggesting similar magnetization transfer and therefore similar mode of binding to ASGPR. In comparison, the scaling factors of monomeric GalNAc are significantly lower indicating the lower affinity of the single sugar moiety compared to the triantennary GalNAc groups of the probes GN‐01, GN‐03, and GN‐05. Additionally, the CH_2_ groups of the aliphatic linker region of these probes show strong, positive STD signals due to binding to ASGPR.

### 2.4 Structural Studies of GN Probes Binding to HSA

As the chemical probes GN‐03 to GN‐06 were designed to interact with HSA to prolong their half‐life in blood circulation, we used X‐ray crystallography and heteronuclear single quantum coherence NMR to confirm and analyze the interactions of the probes with HSA. Our 1.89 Å crystal structure of GN‐07 (an intermediate molecule for GN‐05/6 synthesis, Scheme S4, Supporting Information) bound to HSA reveals polar and nonpolar interactions of both the terminal carboxylate oxygens and the aliphatic moiety of the probe with numerous residues of HSA (Figure 2C). The linker, mostly disordered, does not appear to contribute significantly to binding.

GN‐01, GN‐03, and GN‐05 competed with ^13^C‐methyl‐oleic acid for binding at multiple sites on HSA utilizing an NMR competition assay as described by Krenzel et al.^[^ ^40^ ^]^ This observation demonstrates the strong affinity of GN‐05 for serum albumin (Figure 2D, panel IV), in agreement with the co‐crystal structure of the fatty acid side chain from an intermediate GN‐07 with HSA. The NMR results also validate that GN‐01 does not interact with HSA at all as expected due to the lack of a fatty acid side chain (Figure 2D, panel II). Finally, the shorter protein binding linker present in GN‐03 displayed a weak binding affinity to HSA as the linker is too short to effectively bind GN‐03 to HSA (Figure 2D, panel I).

### 2.5 Hepatocyte Uptake

**Figure 3:**
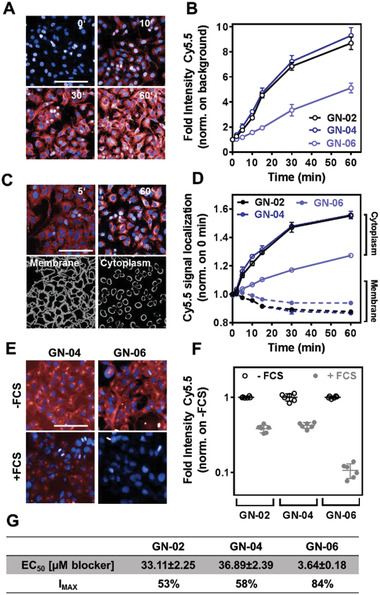
Internalization of Cy5.5‐conjugated GN probes by HepG2 cells. For internalization experiments, cells were incubated for 0, 2, 5, 10, 15, 30, or 60 min with 1 × 10^−6^ m GN‐02, GN‐04, or GN‐06 in fetal calf serum‐free DMEM. A) Representative confocal images taken at 0, 10, 30, and 60 min during GN‐04 incubation. B) Quantification of GN probe internalization based on Cy5.5 signal intensity normalized to background signal. C) Representative confocal images taken at 5 and 60 min during GN‐04 incubation (upper row) and visualization of membrane and inner cytoplasm regions (lower row). D) Quantification of Cy5.5 signal of GN probes on membrane and inner cytoplasm, normalized to 0 min. E) Representative confocal images taken at 60 min during incubation with GN‐04 (left column) or GN‐06 (right column) in the absence of fetal calf serum (FCS) (upper row) or its presence (lower row). F) Quantification of GN probe internalization based on Cy5.5 signal, normalized to the results obtained in the absence of FCS. G) EC_50_ and I_MAX_ for blocking GN probe uptake by HepG2 cells in the absence of fetal calf serum. In all micrographs, red color corresponds to Cy5.5 signal; blue color, to DAPI staining of nuclei. Data shown are mean ± SD (*n* = 4). Scale bar, 100 µm.

Next, we confirmed that the chemical probes are taken up by hepatocytes in culture. HepG2 human hepatoma cells, which express ASGPR like normal hepatocytes, were incubated with Cy5.5‐labeled optical imaging probes (GN‐02, GN‐04, or GN‐06), and the localization and intensity of fluorescence signal was monitored over time using confocal microscopy (**Figure** **3**A,B). Signal intensity increased with time without affecting cell viability (Figure S3, Supporting Information). A greater amount of GN‐02 and GN‐04 entered the cytoplasm than GN‐06 (Figure 3C,D). Consistent with the ability of serum albumin to “retain” the GN probe containing the longer side chain palmitic acid, we found that adding 5% fetal calf serum to the medium during HepG2 cell incubation with the probes reduced internalization of GN‐06 by 85% (Figure 3E–G).

To assess whether the internalization of the GN probes was mediated by specific ASGPR receptors, we preincubated HepG2 cells with GN‐A followed by the chemical probes GN‐02, GN‐04, or GN‐06 and the update of the chemical probes was significantly reduced. Here, GN‐A has been used as ASGPR blocker that binds to ASGPR with much higher affinity (*K*_d_ = 5.8 × 10^−9^ m) than the GN probes (*K*_d_ = 1.5‐1.9 × 10^−6^ m). This shows that the update of the chemical probes is receptor‐mediated, and the three optical imaging probes showed apparent half‐maximal effective concentrations in the micromolar range for interacting with the putative internalization receptor (Figure 3G; Figure S4, Supporting Information).

### 2.6 In vivo Biodistribution and Liver Targeting

**Figure 4:**
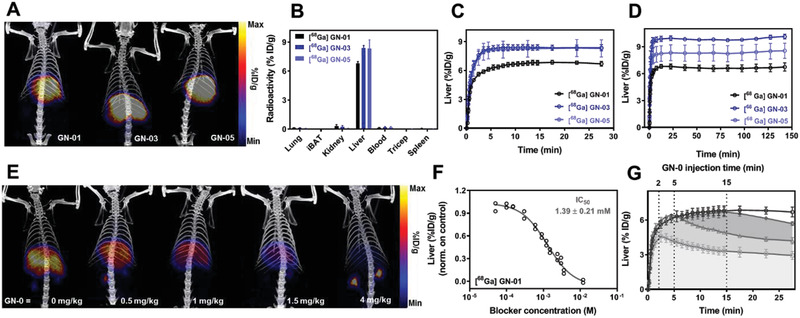
Biodistribution of [^68^Ga] GN‐01, GN‐03, and GN‐05 in Sprague–Dawley rats. In each experiment, a single intravenous bolus of [^68^Ga] GN probes (20 µg kg^−1^, 5 ± 0.5 MBq) was administered and PET scans were performed. A) Representative PET‐CT images after 30 min. B) Uptake of [^68^Ga] GN‐01 (*n* = 6), [^68^Ga] GN‐03 (*n* = 5), and GN‐05 [^68^Ga] (*n* = 6) into the indicated tissues at 30 min. All probes were taken up extensively by the liver. C) Uptake of [^68^Ga] GN‐01, [^68^Ga] GN‐03, and [^68^Ga] GN‐05 by the liver over time (*n* = 3). D) Uptake of [^68^Ga] GN‐01, [^68^Ga] GN‐03, [^68^Ga] GN‐05 in liver over time (*n* = 3). E) Dose‐dependent blocking of [^68^Ga] GN‐01 radioactivity signal in liver. Animals were given blocker (GN‐A) at the indicated doses followed 30 s later by [^68^Ga] GN‐01, then PET‐CT images were recorded at different time points. Here we show PET‐CT images at 30 min time points. F) IC_50_ for [^68^Ga] GN‐01 binding to ASGPR in liver in vivo. Each data point comes from a single animal at 30 min (*n* = ≤3). G) Chase study in which animals received [^68^Ga] GN‐01 followed by high‐dose (4 mg kg^−1^) blocker GN‐A after 2 min (red), 5 min (green), and 15 min (brown) (*n* = 3 at all‐time points). After 15 min, most [^68^Ga] GN‐01 could not be chased. Data shown are mean ± SEM.

Before investigating the progression of NASH with the selective GN chemical probes, it was essential to verify the in vivo biodistribution and liver targeting ability of these GN probes in healthy nonfasted SD rats using PET imaging. Hence, healthy nonfasted SD rats received a single intravenous bolus of [^68^Ga] GN probes and then radioactive uptake by the kidney, lungs, liver, brown adipose tissue, blood, triceps muscle, and spleen were analyzed up to 150 min after dosing. All [^68^Ga] GN probes were taken up more efficiently by the liver within 10 min, and the radioactivity persisted in the liver through to the final measurement at 150 min (**Figure** **4**A–C). Small amounts of radioactivity were observed in kidney and blood, while no radioactivity was detectable in the other tissues examined (Figure 4B).

To confirm the liver‐specific uptake of the [^68^Ga] GN probes, we first injected rats with the GN‐A blocker followed 30 s later by [^68^Ga] GN probes. A single intravenous bolus of 4 mg kg^−1^ GN‐A (blocker) almost abolished liver uptake of [^68^Ga] GN‐01 and moderately inhibited liver uptake of [^68^Ga] GN‐03 and GN‐05 (Figure S5, Supporting Information). These results are consistent with the design that neither GN‐01 nor GN‐A interacts with proteins in the blood and so are cleared from circulation much faster than GN‐03 and GN‐05. Further studies showed that GN‐A blocked liver uptake of [^68^Ga] GN‐01 in a dose‐dependent manner (Figure 4E,F), with [^68^Ga] GN‐01 showing an estimated half maximal inhibitory concentration of 1.39 ± 0.21  × 10^−3^ m.

Next, we reversed the order of injection of probe and blocker in order to determine how quickly [^68^Ga] GN‐01 was internalized by the liver. Animals were injected first with the probe, followed by the GN‐A blocker at 2, 5, or 15 min later. This “chase” with blocker at 2 or 5 min substantially reduced the amount of radioactivity in the liver, while the chase experiment at 15 min reduced the radioactivity only slightly (Figure 4G). This decrease of liver radioactivity was accompanied by an increase of radioactivity in kidneys and blood (Figure S6, Supporting Information). We conclude that under these experimental conditions, most probes bound to ASGPR on the hepatocyte surface and were internalized within approximately 15 min. Before then, blocker could “chase” probe off the ASGPR and release it into the circulation.

Hepatocyte‐specific uptake of [^68^Ga] GN probes was confirmed in CD‐1 mice, which, due of their small size, allowed the detection of probe accumulation in the urinary bladder (Figure S7A,B, Supporting Information). These results based on PET imaging of [^68^Ga] distribution in rats and mice were confirmed using tomographic fluorescence imaging of Cy5.5‐conjugated GN‐02 and GN‐06 with the IVIS Spectrum system (PerkinElmer) (Figure S8, Supporting Information). In animals injected with GN‐06, 50% of the liver fluorescence at 60 min after injection was still present one week later, likely reflecting the ability of GN‐06 to interact with proteins in the blood.

The results obtained in cell studies as well as in vivo PET and fluorescence tomographic studies reveals that even without the direct protein (HSA) conjugation, our described GN probes target the liver and persist in circulation even longer than we could detect with PET because of the short half‐life of ^68^Ga. This was confirmed using fluorescence labeling. Nevertheless, these results also indicate that the GN probes that interacts with proteins in bloodstream are not very specific to ASGPR and doesn't internalized in hepatocytes as compared to nonprotein binding probes like GN‐01 and GN‐02.

### 2.7 Development of an Obese, Insulin‐Resistant ZSF1 Rat Model of NASH

In order to apply the GN probe to NASH monitoring via PET imaging, a rat model that more closely resembles the metabolic and hepatic defects in NASH in humans needed to be developed. Available rodent models of NASH and fibrosis typically require genetic modifications, special diets, hepatotoxins, or some combination of these (Table S3, Supporting Information).^[^ ^12^, ^13^, ^14^, ^15^, ^16^, ^17^ ^]^ These models develop NASH‐like steatosis, inflammation and fibrosis, but the fibrosis is typically moderate and although the animals may be obese, they often do not show robust insulin resistance. Restricted diet or hepatotoxins can lead to more advanced fibrosis but, unlike in human NASH patients, they also lead to weight loss and diabetes regression.

**Figure 5:**
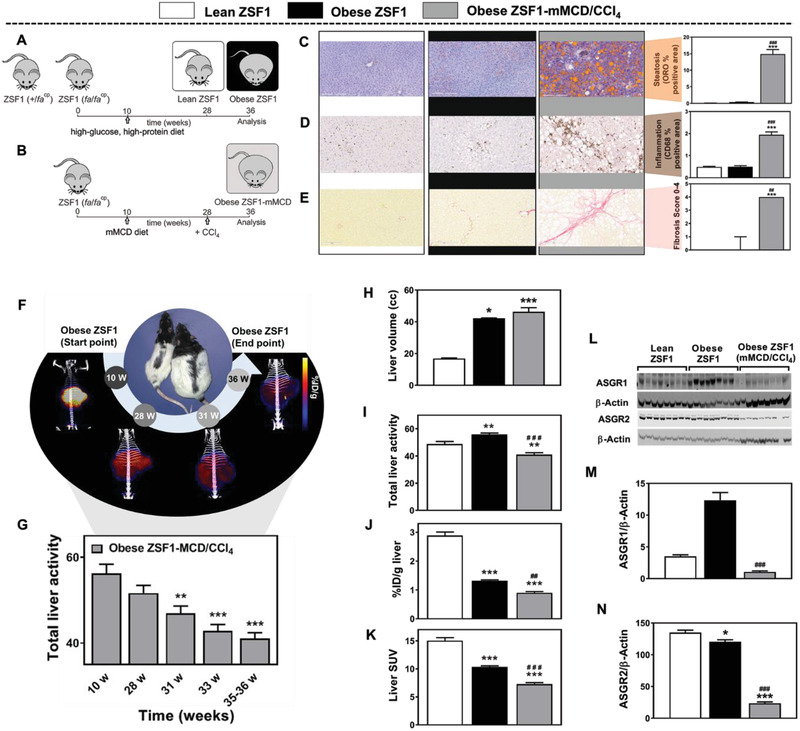
Noninvasive imaging of hepatocyte function in the ZSF1 rat model of NASH. A) ZSF1 (fa/fa^cp^) and ZSF1 (+/fa^cp^) animals served as controls. They were fed a standard high‐glucose, high‐protein diet but not exposed to hepatotoxin. The ZSF1 (fa/fa^cp^) animals became obese, while the ZSF1 (+/fa^cp^) animals remained lean (metabolically normal). B) ZSF1 (fa/facp) rats were fed a modified methionine/choline‐deficient diet (0% choline, 0.2% methionine) for 17 weeks together with administration of the hepatoxin CCl4 for 9 weeks. Histopathology of liver tissue showed that of the three rat groups, obese ZSF1‐mMCD/CCL4 rats mimicked the NASH phenotype best in terms of C) steatosis, based on the percentage of section area staining with oil red O (ORO); D) inflammation, based on the percentage of section area staining with anti‐CD68 antibody (clone ED1); and E) fibrosis, based on of area staining with Sirius Red. Scale bars, 200 µm. Data shown are mean ± SEM, except fibrosis scores, which are median ± range. In all PET experiments, a single intravenous bolus of [^68^Ga] GN‐01 (20 µg kg^−1^, 5 ± 0.5 MBq) was administered and PET scans were performed. F) Representative PET‐CT same scale images at different time points. Animal age is indicated in weeks (w). G) Total liver activity (%ID g^−1^ *x* liver volume) of [^68^Ga]‐GN‐01 at 10 w (before start of mMCD diet), 28 w (start of CCl_4_ administration) and of longitudinal follow up time points. H–K) PET showed greater liver volume and lower probe levels in the livers of obese ZSF1‐MCD/CCl_4_ rats than in the other rats based on total liver activity, %ID, and standardized uptake value (SUV). L). Western blotting of ASGR1 and ASGR2. M,N) Quantitation of the Western blot analysis. Data shown are mean ± SEM Differences were analyzed using one‐way ANOVA with Tukey's multiple comparisons test or Kruskal–Wallis test. ^*^ *p* < 0.05, ^**^ *p* < 0.01, ^***^ *p* < 0.001 versus the lean ZSF1 group; ^###^ *p* < 0.001 versus the obese ZSF1 group (*n* = 7–8).

We developed a more phenotypically accurate rodent model of NASH by starting from the Zucker fatty/spontaneously hypertensive heart failure F1 hybrid (ZSF1) rats, which show obesity and progressive diabetes mellitus in the absence of a modified diet or hepatotoxin.^[^ ^41^, ^42^ ^]^ Lean ZS1 (+/fa^cp^) and obese ZSF1 (fa/fa^cp^) animals were used as controls (**Figure** **5**A). The animals were fed a standard high‐glucose, high‐protein diet but not exposed to hepatotoxin. Using a modified methionine/choline‐deficient (mMCD) diet (0% choline, 0.2% methionine) in combination with CCl_4_ on ZSF1 (fa/facp) rats up to 26 weeks (Figure 5B) led to liver steatosis (Figure 5C), inflammation (Figure 5D), and progressive fibrosis (Figure 5E), while the animals remained morbidly obese and insulin‐resistant. This protocol may also induce a more representative NASH‐like phenotype in other rat or mouse strains as well. The complete details and characterization of NASH animal model development can be seen in Figures S9–S14 in the Supporting Information.

### 2.8 Longitudinal PET Analysis of Hepatocyte Function in the Obese ZSF1 Rat Model of NASH

We assessed hepatocyte function in this validated rat model of NASH using [^68^Ga] GN‐01, since this GN probe was specifically taken up by hepatocytes and did not interact with blood proteins. The chemical probe was injected into our rat model, as well as into lean and obese ZSF1 rats fed on the standard diet, as a single intravenous bolus of [^68^Ga] GN‐01 at the constant radioactivity dose. The animals were then monitored for 30 min using PET imaging.

The acquired PET‐CT scans (Figure 5F), and total liver activity (Figure 5G) in the obese ZSF1‐mMCD/CCl_4_ NASH rat model at different time points showed a progressive decrease (≈30%) in the signal (Figure S15C,D, Supporting Information). Comparably, in the control obese ZSF1 rats at 24 and 36 week of age (14 and 26 week of NASH protocol intervention respectively) no changes in total liver activity and standardized uptake value (SUV) was observed (Figure S15C,D, Supporting Information). The sequential decline in total liver activity in the NASH model (obese ZSF1‐mMCD/CCl_4_) was observed even though there was no change in body weight and liver volume from week 28 to 36 (Figure S15A,B, Supporting Information). We observed that final liver volume at 36 weeks was higher in both obese ZSF1 groups compared with the lean ZSF1 group, with no differences between the obese ZSF1 and the obese ZSF1‐mMCD/CCl_4_ (Figure 5H). Conversely, PET analysis showed significantly lower radioactivity (total liver activity, %ID g^−1^ and SUV) in the obese ZSF1‐mMCD/CCl_4_ compared with the lean and obese ZSF1 rats (Figure 5I–K).

These results suggest the downregulation of ASGPR expression or inhibition of ASGPR function during NASH progression. To assess this further, a Western blot analysis showed significantly lower levels of the two ASGPR isoforms in our NASH model than in the obese ZSF1 control rats (Figure 5L–N).

Our work substantially extends a previous study that used an ^18^F‐labeled and fluorescein isothiocyanate‐conjugated monomeric galactose PET probe to track the decrease in ASGPR expression in a rodent model of fibrotic liver disease.^[^ ^43^ ^]^ That reported probe was not restricted to the liver and was eliminated much faster than our [^68^Ga] GN probes. Furthermore, the animal model allowed detection of fibrosis, but data on other NASH features such as steatosis and inflammation were not reported.

In our ZSF1 rat model of NASH, we observed a progressive decrease in [^68^Ga] GN‐01 radioactivity in the liver during disease progression. Since the animals also showed a large increase in body mass and liver volume, mirroring the situation in patients, we normalized our data to body mass (generating the parameter of SUV or liver volume (generating the parameter of “total liver activity”) and still observed a decrease in radioactivity of chemical probe in the liver. This suggests that ASGPR levels in the liver fall during NASH progression and are not simply “diluted” by the increase in fat or glycogen levels. Consistent with this, we observed downregulation of the two ASGPR isoforms, ASGR1 and ASGR2 and also associated with pronounced steatohepatitis, fibrosis and high plasma transaminase activity.

Our NASH‐ZSF1 rat model in combination with our highly liver‐specific ^68^Ga‐labeled GalNAc‐based probe GN‐01 supports repetitive experiments to noninvasively monitor progression of NASH and fibrosis under different situations such as time or diet, or measure the response to treatment interventions, over a long period of time without the need to sacrifice the animals. This approach also supports efforts to reduce the number of animals used for in vivo studies. Using rats rather than mice allows repetitive blood sampling to investigate response to interventions or changes in circulating biomarkers associated with NASH progression, as well as larger amounts of tissue and blood samples for follow‐up analyses.

## 3 Conclusion

Here we describe a liver‐selective PET imaging probe that can be used to quantify gradual changes during NASH progression in our ZSF1 rat model of NASH that recapitulates the obesity and insulin‐resistance characteristic of the disease in humans. The different probes GN‐1‐6 were synthesized in >20 serial chemical steps with high purity (≥95%). Fluorophore containing probes (GN‐02/04/05) were used in in‐vitro studies to determine the selectivity and binding of the pharmacophore. The radioactive gallium‐68 tracer (GN‐01/03/05) showed a sufficient stability in plasma and was applied in in‐vivo studies in rodents. Our PET‐based approach potentially more clinically relevant than other approaches to differentiate steatosis from fibrosis, such as ultrasound based transient elastography, which is less accurate,^[^ ^44^ ^]^ or magnetic resonance elastography, which is not readily available in the clinic. We demonstrate that tracking reduction of ASGPR levels can be useful for monitoring NASH progression, analogous to the way in which ASGPR reduction can be followed by computed tomography to monitor progression of hepatocellular carcinoma and cirrhosis.^[^ ^45^ ^]^We have demonstrated the ability to follow, noninvasively and longitudinally, NASH progression based on gradual downregulation of ASGPR using PET detection of GN probes that specifically target hepatocytes. We validated this approach in the first rat model of NASH that recapitulates the obesity and insulin resistance of the human disease, which allows analysis of the earliest stages of the disease as well as monitoring of the effects and mechanisms of drugs that may slow or reverse this progression. Our structural studies of GN probe binding to ASGPR and HSA provide a basis for probe optimization not only for imaging studies but also potentially for liver‐specific drug delivery via ASGPR.^[^ ^46^, ^47^, ^48^ ^]^

Overall, we describe a complete toolkit including “first‐in‐class” chemical imaging probes to launch new lines of preclinical investigation into the molecular pathways of NASH onset and progression as well as to more effectively explore and understand the impact of treatment intervention in a more relevant in vivo model which will significantly aid the translation of preclinical research into the clinical setting.

## Conflict of Interest

The authors declare no conflict of interest.

## Supporting information

Supporting Information
