# Supplementary material for: Triantennary GalNAc Molecular Imaging Probes for Monitoring Hepatocyte Function in a Rat Model of Nonalcoholic Steatohepatitis
Source: Adv Sci (Weinh). 2020 Nov 9;7(24):2002997. doi: 10.1002/advs.202002997 (PMC7739951; doi:10.1002/advs.202002997)
Supplement: Supplementary file 1 — Supporting Information [file ADVS-7-2002997-s001.pdf]

© 2020 Wiley-VCH GmbH

ADVANCED SCIENCE Open Access

Supporting Information

for *Adv. Sci.,* DOI: 10.1002/advs.202002997

Triantennary GalNAc Molecular Imaging Probes for Monitoring Hepatocyte Function in a Rat Model of Non-alcoholic Steatohepatitis

Anurag Mishra[1,†,*], *Tamara R. Castañeda*[3,†], *Erik Bader*[1], *Bettina Elshorst*[1], *Sheila Cummings*[4], *Petra Scherer*[2], *Dinesh S. Bangari*[4], *Claudia Loewe*[1], *Herman Schreuder*[1], *Christoph Pöverlein*[1], *Mike Helms*[2], *Seth Jones*[1], *Gernot Zech*[1], *Thomas Licher*[1], *Michael Wagner*[1], *Manfred Schudok*[5], *Meltsje de Hoop*[1], *Alleyn T. Plowright*[1,8], *Jens Atzrodt*[6], *Aimo Kannt*[3,7,9], *Iina Laitinen*[2], *Volker Derdau*[1,*]

Triantennary GalNAc molecular imaging probes for monitoring hepatocyte function in a rat model of non-alcoholic steatohepatitis

Anurag Mishra[1,†,*], Tamara R. Castañeda[3,†], Erik Bader[1], Bettina Elshorst[1], Sheila Cummings[4], Petra Scherer[2], Dinesh S. Bangari[4], Claudia Loewe[1], Herman Schreuder[1], Christoph Pöverlein[1], Mike Helms[2], Seth Jones[1], Gernot Zech[1], Thomas Licher[1], Michael Wagner[1], Manfred Schudok[5], Meltsje de Hoop[1], Alleyn T. Plowright[1,8], Jens Atzrodt[6], Aimo Kannt[3,7,9], Iina Laitinen[2], Volker Derdau[1,*]

1. Integrated Drug Discovery, Sanofi-Aventis Deutschland GmbH, Industriepark Höchst, 65926 Frankfurt, Germany

2. Global Bioimaging, Translational In Vivo Models, Sanofi-Aventis Deutschland GmbH, Industriepark Höchst, 65926 Frankfurt, Germany

3. R&D Diabetes, Sanofi-Aventis Deutschland GmbH, Industriepark Höchst, 65926 Frankfurt, Germany

4. Global Discovery Pathology, Translational In Vivo Models, Sanofi Genzyme, The Mountain Road, Framingham, MA 01701, USA

5. R&D Drug Metabolism and Pharmacokinetics, Sanofi-Aventis Deutschland GmbH, Industriepark Höchst, 65926 Frankfurt, Germany

6. R&D Transversal Operations, German R&D Hub, Sanofi-Aventis Deutschland GmbH, Industriepark Höchst, 65926 Frankfurt, Germany

7. Experimental Pharmacology, Medical Faculty Mannheim, University of Heidelberg, 68167 Mannheim, Germany

8. Present address: Wren Therapeutics Ltd, Department of Chemistry, University of Cambridge, Lensfield Rd, Cambridge, CB2 1EW, United Kingdom

9. Fraunhofer IME, Translational Medicine and Pharmacology, 60596 Frankfurt, Germany

[†] These authors contributed equally.

* Corresponding authors: anurag.mishra2@sanofi.com, volker.derdau@sanofi.com

Keywords: NASH, ZSF1 rats, obesity, insulin resistance, ASGPR, PET imaging, GalNAc

Table of Contents

1. Detailed results and discussion from crystallography and NMR **S4-S7**

2. Development of an obese, insulin resistant ZSF1 rat model of NASH. **S8-S9**

Materials and Methods

3. General synthetic experimental details. **S10-S11**

4. **Scheme 1:** Synthesis of intermediates **S12**

5. **Scheme 2:** Synthesis of **GN-01** and **GN-02** **S13**

6. **Scheme 3:** Synthesis of **GN-03** and **GN-04** **S14**

7. **Scheme 4:** Synthesis of **GN-05** and **GN-06** **S15**

8. **Scheme 5:** Synthesis of **GN-A** (**Blocker)** **S16**

9. Synthesis of all intermediates and final **GN-probes**. **S17-S54**

10. General radiochemistry experimental details. **S55-S56**

11. Physicochemical characterization by NMR **S57-S58**

- **a)** HSQC NMR analysis: HSA-GN probes interaction
- **b)** STD NMR analysis: ASGPR-GN probes interaction

12. X-ray diffraction data collection and structure determination **S58-S62**

- **a)** ASGPR – CPDY complex.
- **b)** HSA – CPDY complex

13. *In cellulo* characterization of **GN probes** **S63**

14. Florescence polarization assays. **S63**

15. Animal studies for in vivo biodistribution studies **S63**

16. Animal studies for NASH development **S64**

17. Characterization of the NASH animal model. **S64-S66**

18. *In vivo* PET imaging. **S66-S67**

19. PET image analysis. **S67**

20. PCR and Western blot. **S68**

21. Statistical analysis. **S69**

22. **Figure S1.** Representative radioHPLC chromatogram of

GN probes (GN-01, GN-03, GN-05). **S70**
23. **Figure S2:** HSA and Rat plasma studies of **GN probes** (GN-01, GN-03, GN-05). **S71**
24. **Figure S3:** Cell toxicity studies of **GN probes** (GN-01, GN-03, GN-05). **S72**
25. **Figure S4:** *In vitro* characterization of **GN probes** (GN-02, GN-04, GN-06) on HepG2 cells. **S73**
26. **Figure S5:** Comparative in vivo blocking studies of ^{68}Ga labeled GN probes by PET imaging. **S74**
27. **Figure S6:** *In vivo* chasing studies of GN-01 in SD-Rats. **S75**
28. **Figure S7:** *In vivo* biodistribution studies of GN-01 in mice via PET imaging. **S76**
29. **Figure S8:** *In vivo* biodistribution studies GN-02 and GN-06 in mice via fluorescence imaging (IVIS). **S77**
30. **Figure S9:** Obesity and diabetes **S78**
31. **Figure S10:** Liver NAFLD/NASH. **S79**
32. **Figure S11:** The ZSF1 rat as an obese, diabetic model of NASH. **S80**
33. **Figure S12:** Liver glycogen. **S81**
34. **Figure S13:** Liver NAFLD/NASH – gene expression determination. **S82**
35. **Figure S14:** Serum lipids. **S83**
36. **Figure S15:** Comparative PET analysis for NASH time course. **S84**
37. **Table S3:** **S85-S86**
38. **References** **S87-S89**

Detailed Results Description

1. Detailed results discussion of crystallography and NMR studies

Crystal structure with ASGPR protein. Following the publication of Meier *et al.*[1], we did not crystallize the full-length ASGPR, but only the H1 domain, which contains the GalNAc binding site. The resulting crystals had large solvent channels and an open binding-site, allowing to soak even large trimeric GalNAc molecules like GN-A (**Figure 1A**). The resulting high resolution 1.4 Å electron density maps (**Figure 2A,**) show very clear density for the GalNAc part of GN-A, as well as the first 4-7 atoms of the linker. This clear density for the beginning of the linker shows that we are looking at the compound 28 we soaked in, and not at the GalNAc used for crystallization (see method section later).

The 3-OH of the GalNAc moiety is bound to the calcium ion of ASGPR and is also very close to the calcium ligands Glu252 and Asn264. The 4-OH of the GalNAc moiety is also bound to the calcium ion and is very close to the calcium ligands Gln239 and Asp241. Arg236 makes bidentate hydrogen bonds with the oxygen of the acetyl group and interacts with the oxygen at the 1-position, where the linker is attached. The nitrogen of the N-acetyl group interacts with the side chain of Asn264. Finally, the 5-hydroxymethyl group is stacked onto the indole ring system of Trp243. The linker itself does not have any interactions with ASGPR and becomes quickly disordered.

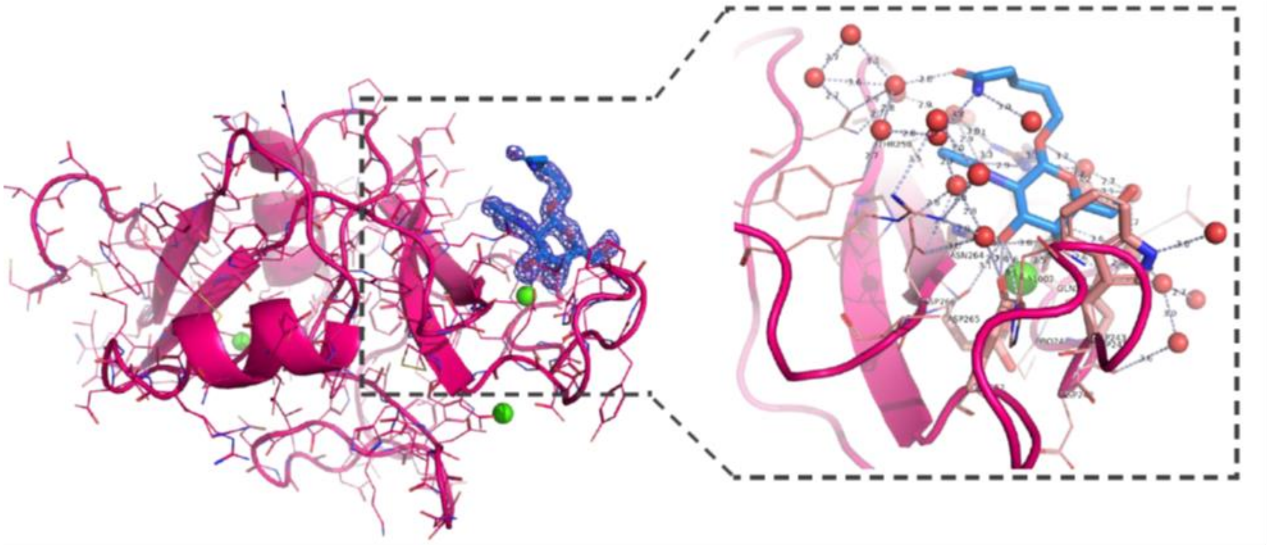

Figure 2A (Extended). Binding of **GN-A** to ASGPR-H1 domain. Left panel: cartoon of the complete ASGPR-H1 molecule (scarlet) with the bound **GN-A** molecule shown in blue, as well as the omit-electron density map contoured at 2σ. Some bound calcium ions are depicted as green spheres. Right panel: close-up of the **GN-A** binding site. Besides direct interactions of **GN-A** with the ASGPR calcium ion and the side chains of Arg236, Trp234 and Asn264, there is network of bound water molecules around the GalNAC binding site that is depicted as well.

Saturation Transfer Difference Spectroscopy (STD) NMR. The binding of GN-01, GN-03, GN-05 to the ASGPR protein was investigated by STD NMR spectroscopy[2], where magnetization is transferred from the protein to its bound ligand and measured by directly observing NMR signals from the ligand itself (**Figure 2B**). Low-power irradiation was applied to 0.4 ppm, a ^{1}H NMR spectral region containing ASGPR protein signals but no ligand signals. The two resulting spectra were subtracted to yield the difference spectrum, which gave positive signals for ligands bound to the protein. For quantification, the STD scaling factor was evaluated by the ratio of the intensities of the STD signals to the signal intensities of the ^{1}H 1D spectrum. The value is arbitrary and depends on the parameter of the NMR experiment but can be used for probes with similar chemical structure to compare the binding mode to ASGPR.

For all investigated GN-probes and GalNAc, STD scaling factors were evaluated for three separate groups of protons: the methyl protons of the acetyl group, the protons of the sugar ring and the protons of the CH_2 groups of the linker (**Figure 2B**). For all trimeric compounds GN-01,

GN-03, GN-05, the scaling factors are very similar, suggesting similar magnetization transfer due to a very similar binding mode of these compounds to the protein ASGPR. Also, the CH_2 groups of the aliphatic linker region (marked in green) show positive STD signals and strengthen the binding to ASGPR. This interaction is missing for single GalNAc and therefore, the STD values are lower. However, the linker region is too flexible to be seen in the X-ray structure (**Figure 2A**).

Crystal structure with HAS. As depicted in **Figure 2C**, our 1.89 Å crystal structure clearly shows electron density for a GN-07 (scheme S4) bound in the fatty acid sites 2 and 8 [3]. The two sites are connected and form a long hydrophobic tunnel, spanning the HSA domains IA and IIA. While the fatty acid part of GN-07 is well defined, the electron density becomes weak and fragmented from the linker onwards, the position where the molecule leaves the binding site, indicating that the linker, as expected, is disordered in the solvent.

The terminal carboxylate oxygens of GN-07 interact with the N_{ε} of Arg257, the OH of Tyr150, the main-chain *N* of Ala258 and a bound water molecule. The aliphatic part of the molecule has van-der-Waals interactions with the side chains of Leu22, Val23, Val46, Phe49, Leu66 and Leu69. The first carbonyl of the linker, at the exit of the binding site, does not have any particularly good interactions with HSA. The formate group has a polar interaction with the side chain of Asn61. The remainder of the linker does not have any interactions with HSA, indicating that the linker does not contribute much to the binding affinity of GN-07.

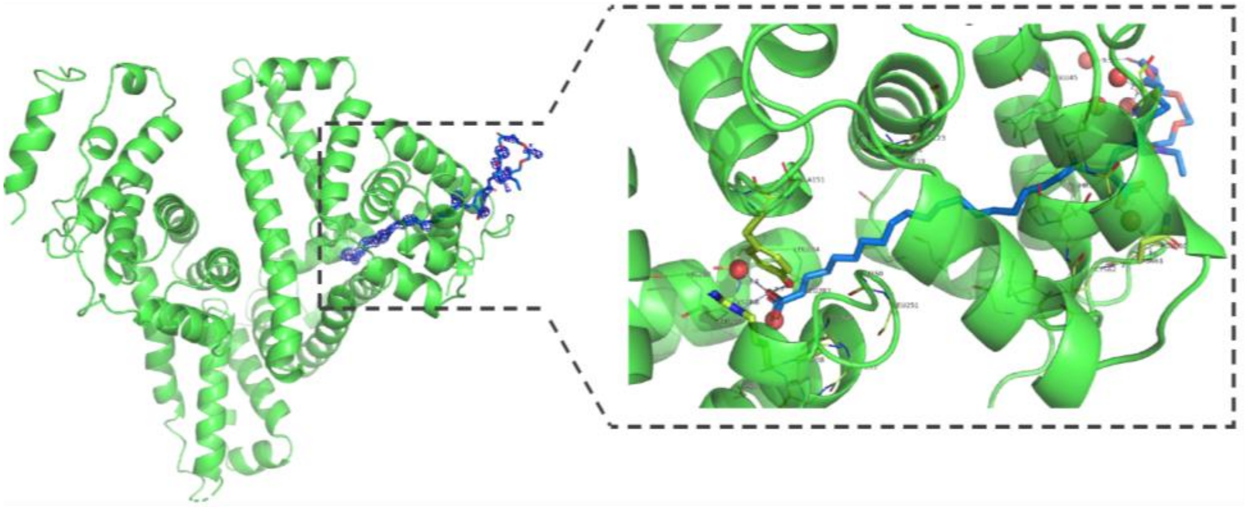

Figure 2C (Extended). Binding of **GN-07** to HSA. Left panel: cartoon of the complete HSA molecule (green) with the bound **GN-07** molecule shown in blue, as well as the omit-electron density map contoured at 2σ. Right panel: close-up of the **GN-07** binding site. On the left is the binding site of the terminal (fatty acid) carboxylate group, on the right is the point where the linker exits the binding site.

HSQC NMR. The binding of GN probes to HSA was investigated by an NMR competition assay according to Krenzel et al.[4]. In the $^{1}H,^{13}C$-HSQC spectrum of this sample, several peaks were observed corresponding to Methyl-^{13}C oleic acid (^{13}C-OA) bound to different binding sites at HSA (**Figure 2D, panel I**). The most intense peaks A, B and C are correlated with the three high affinity sites for fatty acids 2, 4 and 5[5] . Three other peaks D, E and F were identified as drug site 1, drug site 2 and fatty acid site 6.

Addition of GN probes (GN-01, GN-03 and GN-05) led to different competition with the ^{13}C-OA for the binding sites, which can be observed as disappearing peaks in the NMR spectrum. In **Figure 2D, panels II, III and IV** present an overlay of two spectra. The blue spectrum corresponds to HSA saturated with ^{13}C-OA. The overlaid red spectrum is recorded with the same sample after incremental addition of GN probes. Upon addition of compound GN-01, no peaks disappeared in the spectrum because this compound does not contain a fatty acid side chain to interact with HSA (panels II). For GN-03, peak F disappears from the spectrum. This peak is assigned to the drug site 2, which has a very low affinity for oleic acid. Compound GN-03 contains a fatty acid side chain which is suitable for binding to serum albumin. The explanation for this weak binding is that the linker to the soluble GalNAc part of the molecule is very short and the binding to HSA is therefore hampered. In GN-05, several peaks in the HSQC spectrum disappear due to competitive displacement. Peak A, one of the high affinity binding sites for oleic acids is clearly shifted. Peak E and peak G disappear from the spectrum. Peak E is assigned to fatty acid site 6 in human serum albumin. GN-05 displaced oleic acid out of several binding sites of HSA. This observation demonstrates the strong affinity of compound GN-05 to serum albumin which is also in agreement with the co-crystal structure.

2. Development of an obese, insulin resistant ZSF1 rat model of NASH.

The lack of an animal model that recapitulates the complete NASH phenotype, including obesity and impaired glucose metabolism.[6-12] The so-called ob/ob and DIO-NASH mouse models come close, but the amylin diet originally used to induce NASH in these animals[7] is no longer commercially available, and severe fibrosis in these animals is not reproducibly induced by the original amylin diet, a recent modification[9], or a Western diet combined with drinking water containing fructose and glucose. [10] A suitable experimental animal model specifically displaying the pathogenic and therapeutic symptoms with the characteristics of NASH is essential for validation of NASH biomarkers and for the effective establishment of treatment efficacy, which will lead to the accurate monitoring and translation of results from animal studies to experiments in human. Herein, we developed a new and more phenotypically accurate rodent model of NASH by starting from the ZSF1 rat, which shows obesity and progressive diabetes mellitus in the absence of a modified diet or hepatotoxin. Using a modified methionine/choline-deficient diet in combination with CCl4 led to liver steatosis, inflammation and progressive fibrosis, while the animals remained morbidly obese and insulin-resistant.

In order to apply the GN probes to NASH monitoring via PET imaging, we developed a rat model that resembles the metabolic and hepatic defects in NASH closely to humans. We used Zucker fatty/spontaneously hypertensive heart failure F1 hybrid (ZSF1) rats, which are obese and diabetic and mirror the progressive pathology observed in obese diabetic patients, including hypertension, kidney and cardiac complications[13,14]. The animals were fed a modified methionine/choline-deficient (mMCD) diet (0% choline, 0.2% methionine) that prevents the weight loss typically seen on the standard methionine/choline-deficient diet. We confirmed that during 23 weeks on this modified diet, the animals showed a similar obese, diabetic phenotype as the obese ZSF1 rats fed the standard diet for this model **(Figure S9A-D).** The obese ZSF1-mMCD also had higher levels of alanine aminotransferase, aspartate aminotransferase, liver triglycerides and cholesterol **(Figure S10 A-D)**, as well as liver steatosis **(Figure S10E)**. Nevertheless, liver fibrosis was mild, and inflammation was not detectable **(Figure S10F)**.

When we fed ZSF1 rats with mMCD diet for 17 weeks, followed by another 9 weeks on the diet together with the administration of the hepatotoxin CCl_4, the animals remained obese **(Figure 11A)** but not diabetic, instead showing strong insulin resistance **(Figure 11B-C)**. Both

obese ZSF1 groups showed hepatomegaly compared with the lean ZSF1 group **(Figure 11D)**, being more pronounced in the obese ZSF1-mMCD/CCl_4 rats due to dramatically higher liver triglyceride and cholesterol levels **(Figure 11E-F)** instead of higher glycogen levels like in the obese ZSF1 group **(Figure S12)**. Altogether, the obese ZSF1-mMCD/CCl_4 rats recapitulated key features of NASH when compared to the obese ZSF1 rats consuming the standard diet, including higher serum levels of transaminases **(Figure 11G-H);** more severe liver steatosis, inflammation and fibrosis **(Figure 11G-K).** In line with these results, gene expression values for chemokine (C-C motif) ligand 2 (*Ccl2*) (inflammation), transforming growth factorbeta (*Tgfb*) (inflammation/fibrosis) and collagen, type I, alpha 1 (*Col1a1*) (fibrosis) were also significantly higher (**Figure S13A-C)**.

As expected with mMCD consumption, the obese ZSF1-mMCD/CCl_4 rats did not have high serum triglycerides as observed in human NASH. However, this feature was also present in other models with different diets[15-17]. Although triglycerides were low, serum free fatty acids (FFA) were significantly higher, as observed in NAFLD, in the ZSF1-mMCD/CCl_4 compared with the standard diet lean and obese ZSF1 rats. This was in line with the higher liver steatosis observed in the obese ZSF1-mMCD/CCl_4 rats. Furthermore, the modification of the MCD allowed for higher serum cholesterol in the obese ZSF1-mMCD/CCl_4 compared with the lean ZSF1 rats, although still lower than in the obese ZSF1 rats (**Figure S14A-C**).

Martials and Methods

2. General synthetic experimental details.

Reagents and Solvents.

All solvents and reagents were purchased at analytical grade from their respective commercial suppliers (Aldrich, Fluka, Merck, Strem and VWR) and were used without further purification unless otherwise stated. All air or water-sensitive chemicals were stored under an inert atmosphere. Water refers to high purity water obtained from the "PuriteSTILL Plus" purification system, with conductivity of 0.04 µS cm- 1.

Spectroscopy.

All NMR spectra (1D ^{1}H and ^{13}C, COSY, HSQC, HMBC and ROESY), were recorded on a Bruker Avance III 300 MHz and Bruker Avance-Neo 700 MHz spectrometer (Bruker, Germany) at 300 K. Chemical shifts are reported in ppm with trimethylsilyl-3-propionic acid D4-2,2,3,3 as the internal standard. Coupling constants are reported in Hz and refer to ^{3}J H-H couplings, unless otherwise stated. Spectra were recorded in commercially available deuterated solvents.

Electron Spray Ionisation-Low Resolution Mass Spectroscopy (ESI-LRMS in positive and negative ion mode) were carried out on Advion CMS Mass spectrometer with ESI ion source, Advion, Ithaka, NY connected to an Agilent 1260 HPLC, Agilent Waldbronn, Germany. Electron Spray Ionisation-High Resolution Mass Spectroscopy (ESI-HRMS in positive and negative ion mode) were carried out Bruker µTOF-Q II with ESI ion source, Bruker Daltonik, Bremen, Germany connected to a Dionex Ultimate 3000RS HPLC, Thermo/Dionex, Germering, Germany.

Chromatography.

Thin layer chromatography was performed on neutral aluminium sheet silica gel plates (Merck Art 5554) and visualised under UV irradiation (254 nm), Dragendorff reagent and sulfuric acid staining.

Preparative reverse phase high performance flash chromatography was performed on Teledyne Isco CombiFlash® NextGen 100 by using various size of RediSep Rf Gold C18 Columns.

Reverse phase preparative HPLC were performed on a Gilson apparatus (131 pump equipped with 171 Diode Array Detector) with Luna 10 microns C18(2) 100 A, 100 x 21.20 mm column (big column that purify up to 30 mg) /Supelco Acentis® C18 15cm x 10mm 5 microns column (small column that purify up to 10 mg). A gradient elution with a solvent system composed of H_2O + 0.1% CF_3COOH/ACN was performed for a total run time of 20 min.

3. **Scheme 1:** Synthesis of ***GalNAc.NASH*** intermediates**.**

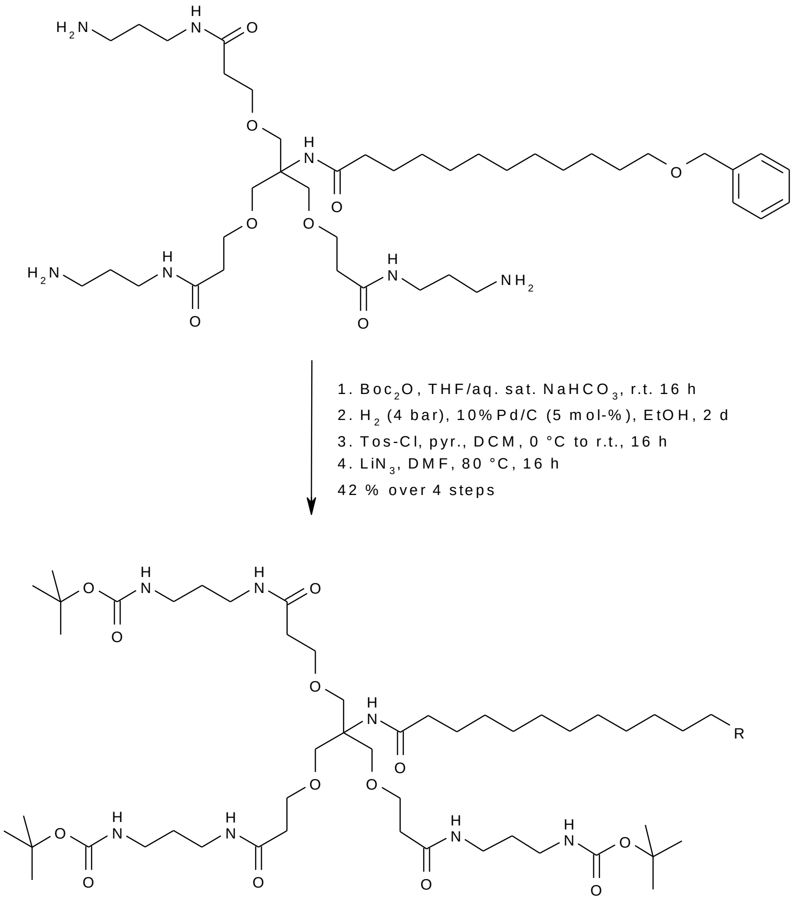

1: R = OBn
2: R = OH
3: R = OTos
4: R = N$_3$

4. Scheme 2: Synthesis of *GalNAc.NASH-01* and *GalNAc.NASH-02*

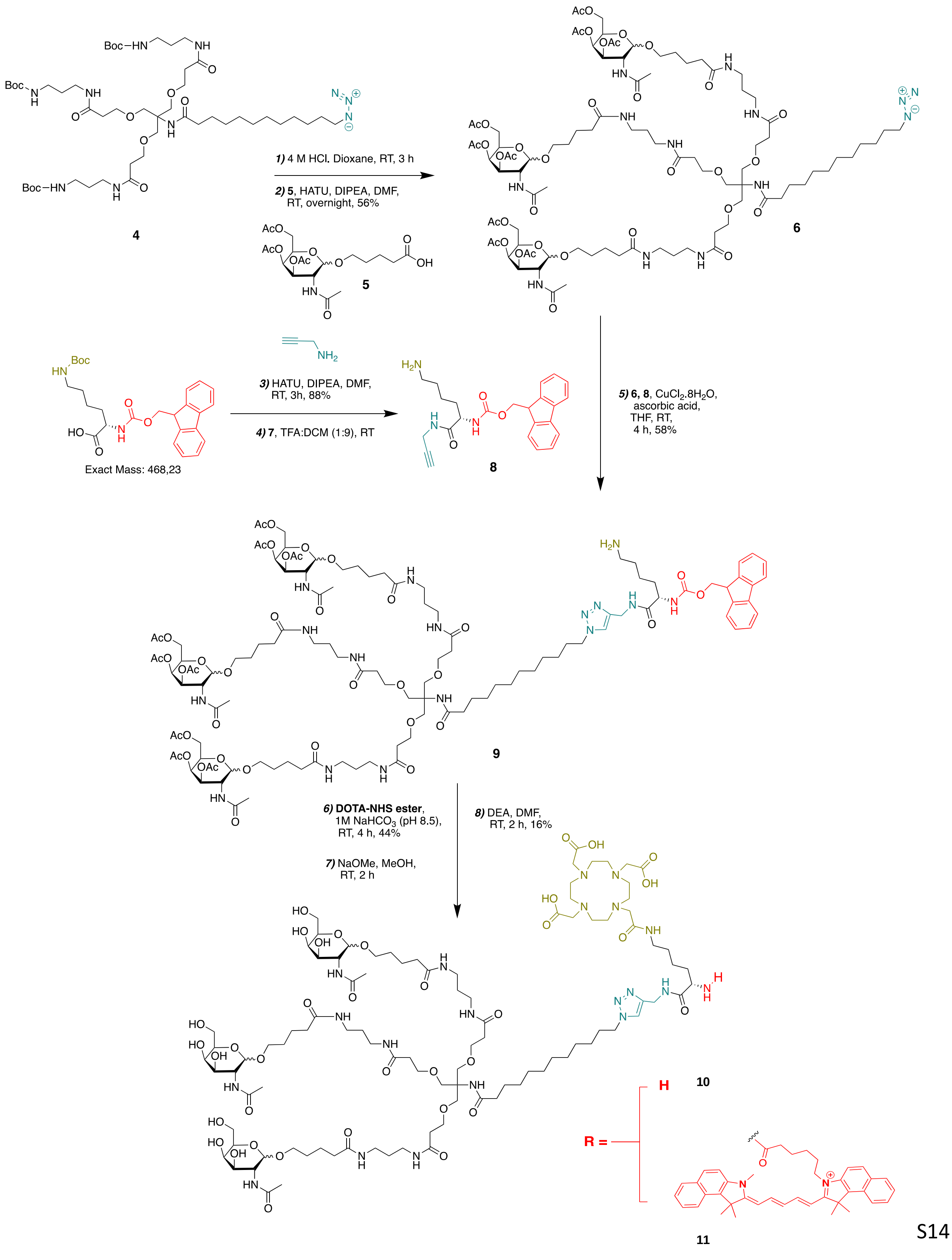

5. Scheme 3: Synthesis of *GalNAc.NASH-03* and *GalNAc.NASH-04*

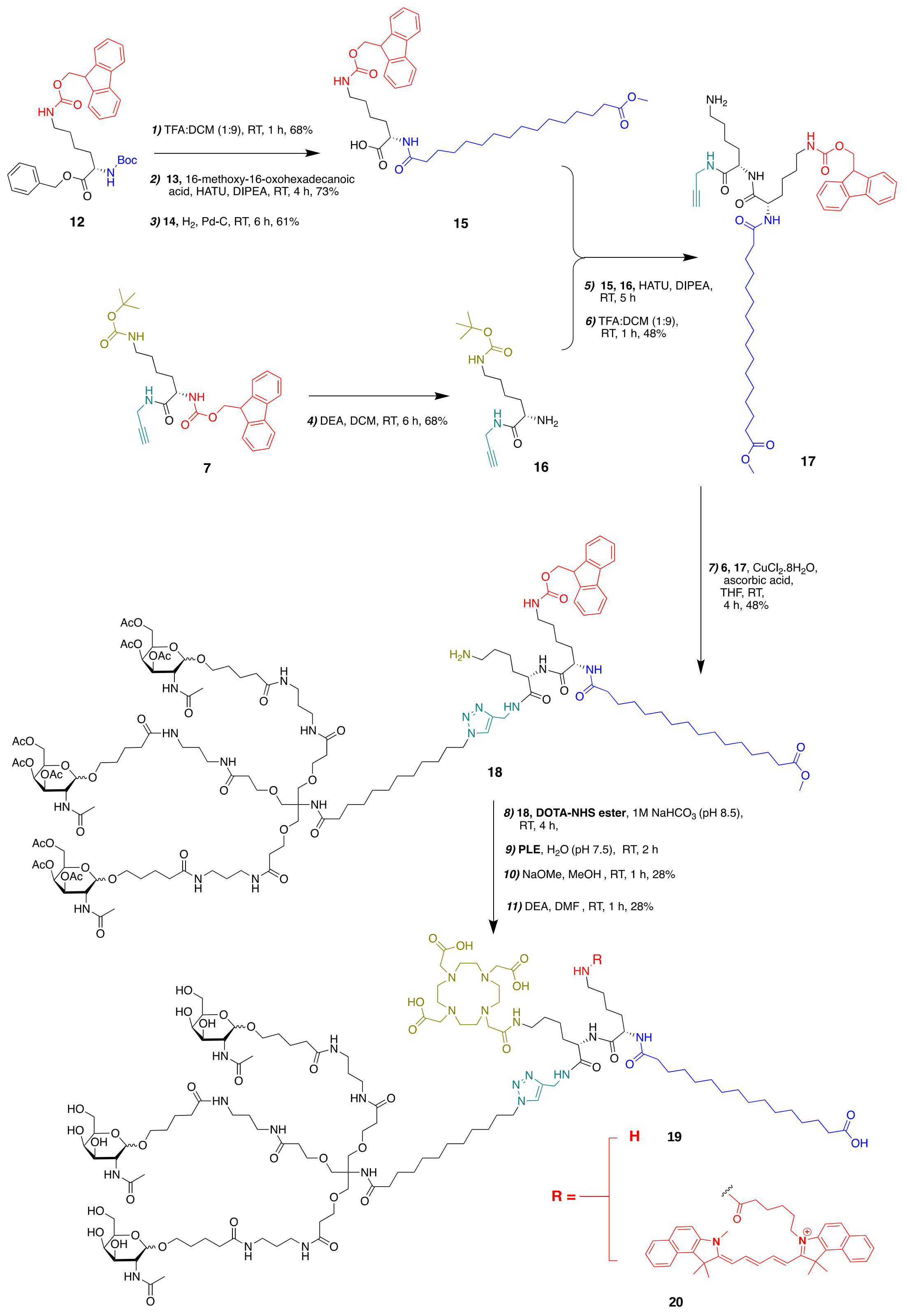

6. Scheme 4: Synthesis of *GalNAc.NASH-05* and *GalNAc.NASH-06.*

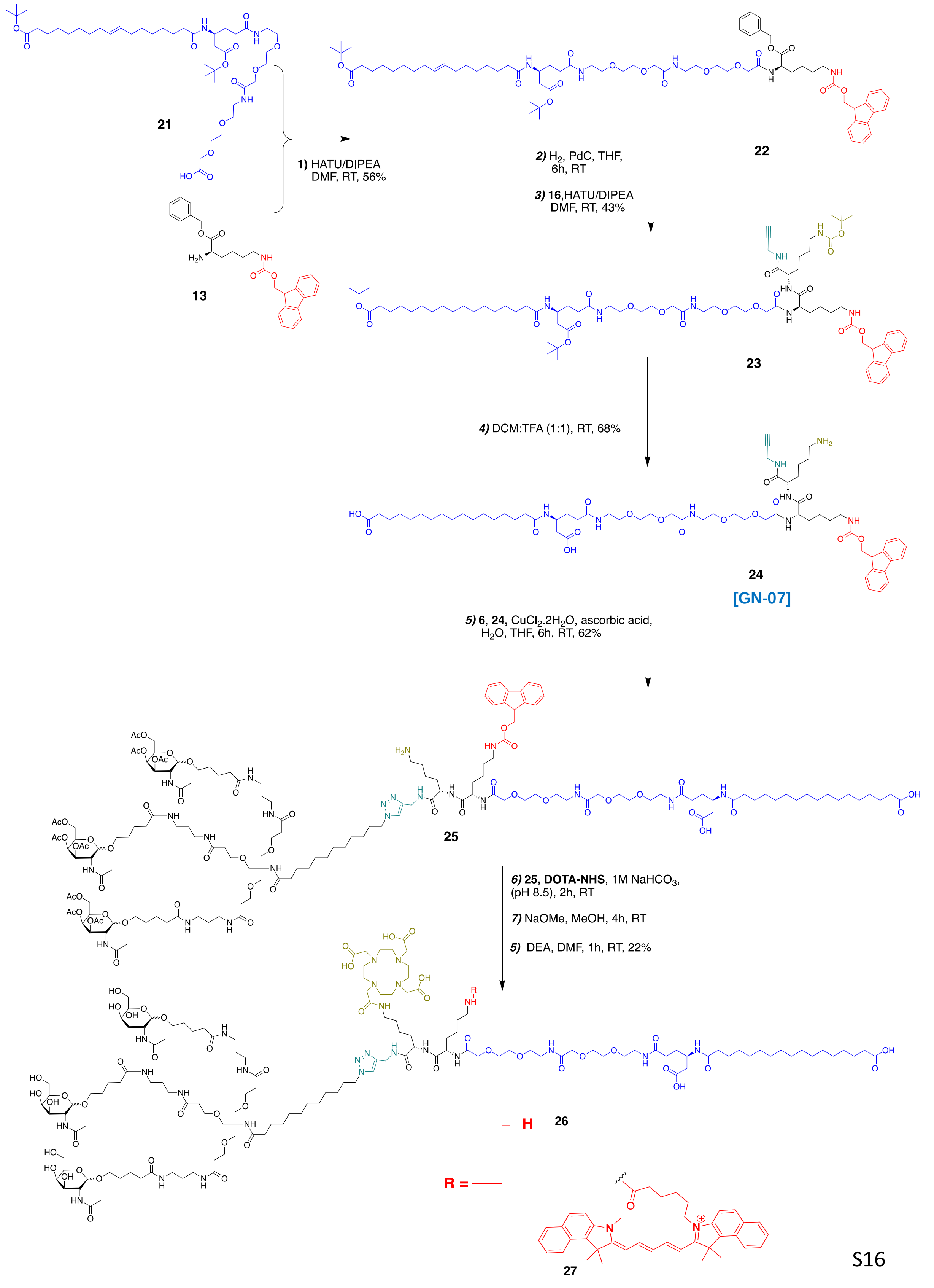

7. Scheme 5: Synthesis of GN-0 (***Blocker)***

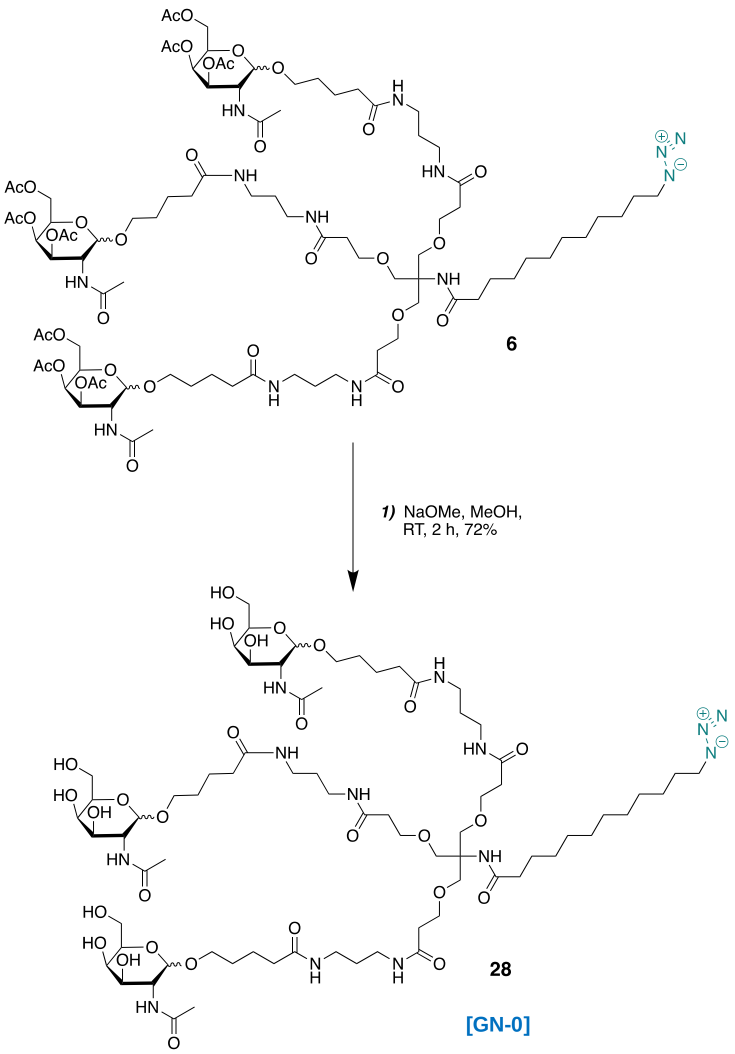

28

[GN-0]

8. Synthesis of all intermediates and final GalNAc.NASH probes.

Compound 2: The triamine starting material (trihydrochloride, 2.025 g, 2.24 mmol) was dissolved in a mixture of THF (40 mL) and aq. sat. NaHCO3-solution (20 mL) and Boc2O (2.99 g, 13.70 mmol, 6.11 equiv.) were added. The reaction mixture was stirred over night until LC/MS indicated complete Boc-protection. EtOAc (100 mL) and water (20 mL) were added, the layers were separated, the organic layer was washed with brine (30 mL), dried (MgSO4), filtered and concentrated in vacuo. The crude product of the Boc-protected compound 1 was directly used for the hydrogenation.

The crude Boc-potected product **1** (max. 2.24 mmol) was dissolved in EtOH (100 mL), 10% Pd/C (118 mg, 112 µmol, approx. 5 mol-%) were added and the mixture was hydrogenated in an autoclave at r.t. and 4 bar hydrogen gas for 2 d. After complete benzylether deprotection was monitored by LC/MS, the reaction mixture was filtered and concentrated *in vacuo*. The crude product of free alcohol **2** was directly used for the next step without further purification. For analytical purpose, a small sample of the crude product was purified by HPLC chromatography.

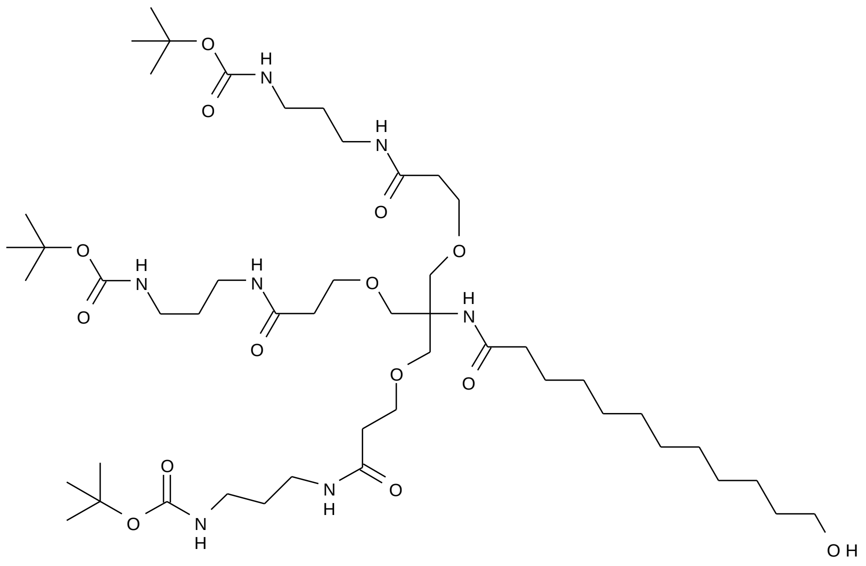

2

Atom#	C Shift	XHn	H Label	H Shift	H Multiplicity
14	59,504	C			
16		NH	M12	6,362	br s
20	174,057	C			
21	37,209	CH2	H 29	2,161	br t (6.59, 6.59)
22	32,701	CH2	H 30	1,558	m
30	25,666	CH2	H 41	1,329	m
31	25,692	CH2	H 40	1,560	m
32	62,722	CH2	H 32	3,614	m
1, 42, 53	156,547	C			
10, 33, 44	67,427	CH2	H 19	3,686	br t (4.90, 4.90)
13, 15, 17	69,626	CH2	H 21	3,664	br s
2, 41, 52		NH	M11	5,345	m
23, 24, 25, 26, 27, 29	29,346	CH2	H 38	1,261	br d (7.54)
4, 40, 51	37,209	CH2	H 25	3,139	m
5, 39, 50	30,019	CH2	H 27	1,635	br d (4.94)
59, 63, 67	79,181	C			
6, 38, 49	36,112	CH2	H 26	3,278	m
60, 61, 62, 64, 65, 66, 68, 69, 70	28,412	CH3	H 23	1,423	br s
7, 37, 47		NH	H 28	7,020	br s
8, 35, 46	171,806	C			
9, 34, 45	36,669	CH2	H 22	2,419	br s

Compound 3. To a solution of the crude alcohol 2 (max. 2.24 mmol) in DCM (20 mL) at 0 °C were added pyridine (2.4 ml, aprox. 10 equiv.) and tosyl chloride (854 mg, 4,48 mmol, approx. 2.0 equiv.). The reaction mixture was stirred over night to warm up slowly to r. t. After 16 h, the reaction was stopped by addition of water (1 mL). After 15 min of stirring, EtOAc (100 mL) and 1 N HCl (50 mL) were added, the layers were separated, the organic layer was washed with brine (30 mL), dried ($MgSO_4$), filtered and concentrated in vacuo. The crude product of tosylate 3 was used without further purification for the next reaction. For analytical purpose, a small sample of the crude product was purified by HPLC chromatography.

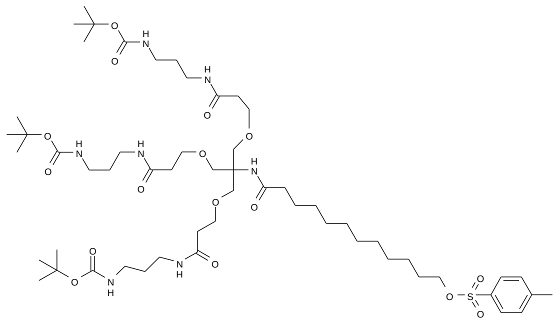

3

Atom#	C Shift	XHn	H Label	H Shift	H Multiplicity
14	59,501	C			
16		NH	M20	6,352	m
20	174,019	C			
21	37,201	CH2	H 24	2,169	br t (7.59, 7.59)
22	25,766	CH2	H 33	1,571	m
30	25,715	CH2	H 35	1,341	m
31	28,820	CH2	H 31	1,592	m
32	70,749	CH2	H 8	4,013	m
62	133,317	C			
65	144,687	C			
68	21,641	CH3	H 37	2,456	s
1, 42, 53	156,561	C			
10, 33, 44	67,436	CH2	H 29	3,695	t (1.00, 1.00)
13, 15, 17	69,650	CH2	H 30	3,673	s
2, 41, 52		NH	H 14	5,333	m
23, 24, 25, 26, 27, 29	29,333	CH2	H 34	1,276	br d (9.80)
4, 40, 51	37,221	CH2	H 10	3,142	br d (5.55)
47, 7, 37		NH	H 17	6,983	br s
5, 39, 50	30,064	CH2	H 15	1,631	m
5, 39, 50		CH2	M16	1,622	m
6, 38, 49	36,104	CH2	H 9	3,297	m
63, 67	127,860	CH	H 2	7,784	d (8.15)
64, 66	129,830	CH	H 3	7,349	d (8.06)
69, 73, 77	79,203	C			
70, 71, 72, 74, 75, 76, 78, 79, 80	28,436	CH3	H 13	1,432	s
8, 35, 46	171,800	C			
9, 34, 45	36,700	CH2	H 28	2,427	m

Compound 4 [di-*tert*-butyl (10-(12-azidododecanamido)-10-(13,13-dimethyl-5,11-dioxo-2,12-dioxa-6,10-diazatetradecyl)-5,15-dioxo-8,12-dioxa-4,16-diazanonadecane-1,19-diyl)dicarbamate]: The crude tosylate 3 (max. 2.24 mmol) was diluted in DMF (3 mL), a 2N LiN_3 solution in DMF (2.3 mL, 4.6 mmol, approx. 2.0 equiv.) was added and the reaction mixture was heated over night for 16 h at 80 °C. The reaction mixture was filtered and directly purified by HPLC chromatography (15 min gradient, H_2O: MeCN 60 to 99%, 10 runs). The product containing fractions were collected, concentrated in vacuo to remove the acetonitrile and freeze-dried to obtain the desired product **4** (941 mg, 915 µmol, 42 % over 4 steps) as a colorless oil.

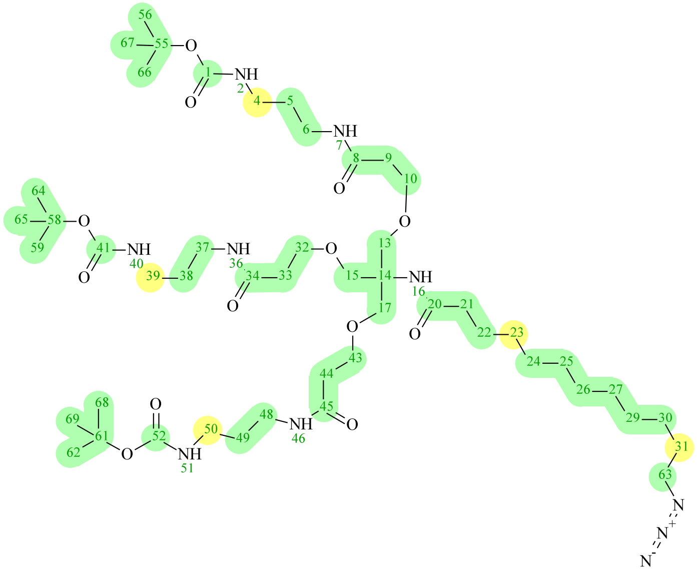

4

Atoms#	C Shift [ppm]	XHn	H Shift	C/X Multiplicity	H Multiplicity
31	24,053	CH_2	1,592	m	m
23	24,998	CH_2	1,374	s	q (1.00, 1.00, 1.00)
56, 59, 62, 64, 65, 66, 67, 68, 69	26,727	CH_3	1,454	s	s
22, 31	27,121	CH_2	1,618	s	m
24, 25, 26, 27, 29, 30	27,432	CH_2	1,303	s	br s
5, 38, 49	28,437	CH_2	1,659	s	quin (6.07, 6.07, 6.07, 6.07)
6, 37, 48	34,256	CH_2	3,320	u	q (6.00, 6.00, 6.00)
6, 37, 48	34,363	CH_2		s	
44, 9, 33	35,046	CH_2	2,444	s	t (5.59, 5.59)
21	35,477	CH_2	2,186	s	t (1.00, 1.00)
4, 39, 50	35,550	CH_2	3,176	s	br q (6.00, 6.00, 6.00)
63	49,778	CH_2	3,273	s	t (6.98, 6.98)
14	57,772	C		s	
10, 32, 43	65,730	CH_2	3,713	s	br t (5.59, 5.59)
13, 15, 17	68,000	CH_2	3,695	s	s
55, 58, 61	77,516	C		s	
1, 41, 52	154,839	C		br s	
8, 34, 45	169,983	C		s	
20	172,216	C		s	
2, 40, 51		NH	5,241		br t (5.51, 5.51)
16		NH	6,284		s
7, 36, 46		NH	6,876		t (1.00, 1.00)

HRMS (ES^+) m/z $C_{49}H_{92}N_{10}O_{13}$ requires 1029.67 $[M+H]^+$; found 1029.6971 $[M+H]^+$.

Compound 5 [5-(((3*R*,4*R*,5*R*,6*R*)-3-acetamido-4,5-diacetoxy-6-(acetoxymethyl)tetrahydro-2*H*-pyran-2-yl)oxy)pentanoic acid]: To a solution of O-Acetyl N-Acetyl-D-galactosamine (2 g, 5.14 mmol) in DCE (20 mL) was added TMSOTf (1.7 g, 1.33 mL, 7.71 mmol) dropwise at 15°C. The mixture was heated to 50°C for 1 h and then stirred at 30°C overnight. The reaction mixture was poured into saturated $NaHCO_3$ (100 mL) and extracted with DCM (200 mL*2). The combined organic layers were dried over anhydrous Na_2SO_4, filtered and concentrated in vacuo. The residue was dissolved in DCE (20 mL), 5-hydroxy-1-benzylpentanoate (1.6 g, 7.71 mmol) and 4A sieves powder (0.5 g) was added at 15°C. After stirred at this temperature for 1 h, TMSOTf (0.57 g, 0.45 mL 2.57 mmol) was added dropwise at 15°C. The suspension was then stirred at room temperature for overnight. The reaction mixture was poured into sat. $NaHCO_3$ solution (100 mL), filtered and extracted with DCM (200 mL*2). The combined organic layers

were dried over Na_2SO_4, filtered and concentrated in *vacuo*. The residue was purified by CombiFlash high performance flash chromatography (5% MeOH in DCM, R_f = 0.55) to give benzylated intermediate as a colorless oil (1.19 g, 43%).

Now a solution of purified intermediate (1.1 g, 2.05 mmol) in MeOH (20 mL) was added 10% Pd/C (0.1 g). The mixture was stirred at room temprature under H_2 (40 psi) overnight. TLC showed no starting material was remained. The reaction mixture was filtered, and the filtrate was concentrated in vacuo to give compound **5** (0.915 g, yield 100% w/w) as white solid.

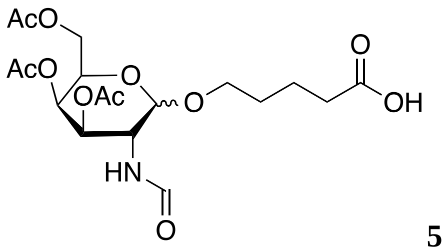

5

^{1}H NMR (300 MHz, CHLOROFORM-*d*) δ ppm: 1.59 - 1.76 (m, 4H), 1.94 - 1.99 (s, 3H), 2.02 (s, 3H), 2.06 (s, 3H), 2.18 (s, 3H), 2.30 - 2.40 (m, 2H), 3.48 - 3.57 (m, 2H), 3.92 - 3.98 (m, 2H), 4.10 - 4.16 (m, 1H), 4.69 (d, J=8.3 Hz, 1H), 5.30 (dd, J=3.4, 11.2 Hz, 1H), 5.37 (d, J=3.1 Hz, 1H), 6.19 (d, J=8.8 Hz, 1H).

MS (ES^+) m/z $C_{19}H_{29}NO_{11}$ requires 448.18 $[M+H]^+$; found 448.23 $[M+H]^+$

Compound 6: A solution of compound **4** (1 g, 0.97 mmol) was dissolved in CH_2Cl_2 (7 mL), TFA (3 mL) was added and stirred at room temperature for 1 h. After completion, the reaction mixture was evaporated, dissolved in CH_2Cl_2 (10 ml) and evaporated under reduced pressure to give amine derivative as a light-yellow sticky oil. Now, a solution of compound **5** (1.5 g, 3.35 mmol), DIPEA (0.86 mL, 4.94 mmol) and HATU (1.25 g, 3.29 mmol) in anhydrous DMF (5 mL) was stirred at room temp for 1 h. After this, crude amine residue (0.6 g, 0.82 mmol) was added in a reaction mixture and was stirred at room temp for overnight. The completion of reaction was verified by ESI-LRMS, the residue was purified by RP-HPLC and lyophilized to give compound **6** as an off-white solid (0.93 g, 56%).

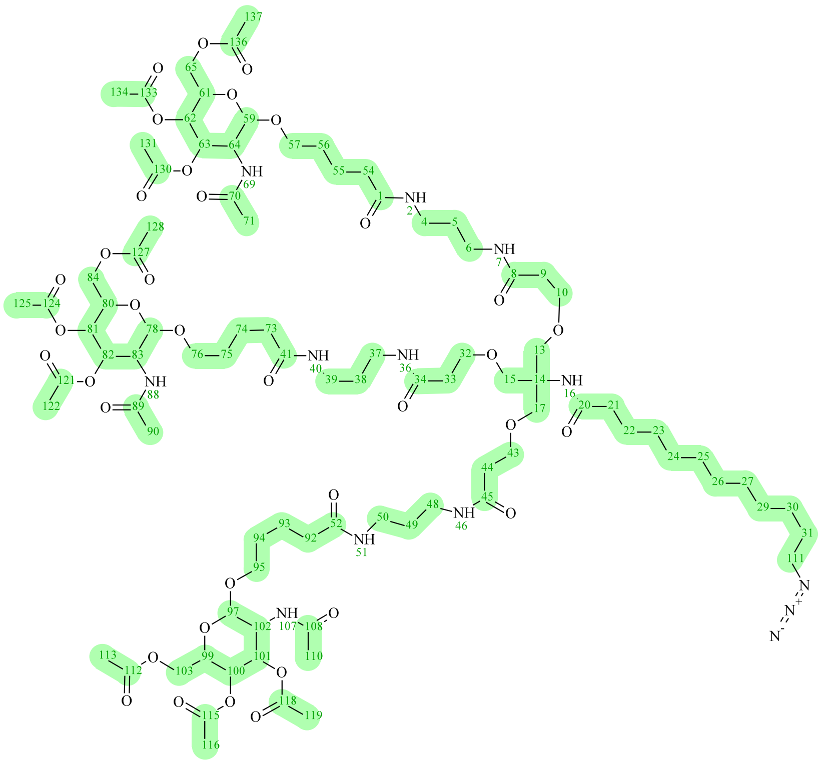

6

Atoms #	C Shift [ppm]	XHn	H Shift [ppm]	C Calc Shift (Neural Net)	H Calc Shift (Neural Net)	H Multiplicity
	17,427	CH	1,413			d (6.68)
	18,555	CH	1,456			m
	20,639	CH	2,178			m
113, 128, 137	20,677	CH_3	2,055	21,010	2,069	s
119, 122, 131	20,678	CH_3	2,005	21,019	2,037	s
116, 125, 134	20,683	CH_3	2,155	20,792	2,042	m
	20,920	CH	2,095			br s
55, 74, 93	22,575	CH_2	1,721	22,569	1,618	m
71, 90, 110	23,269	CH_3	1,960	23,195	1,954	s
22	25,770	CH_2	1,569	26,144	1,608	m
31	26,684	CH_2	1,374	29,685	1,593	m
56, 75, 94	28,352	CH_2	1,615	29,732	1,519	m
	28,807	CH_2	1,606			m
	29,097	C or CH_2				
30	29,281	CH_2	1,314	26,296	1,222	m
	29,396	C or CH_2				
	29,417	C or CH_2				
23, 24, 25, 26, 27, 29	29,479	CH_2	1,283	29.236, 29.094, 28.776, 28.930, 29.017, 28.884	1.220, 1.114, 1.159, 1.139, 1.217, 1.189	br s
5, 38, 49	29,602	CH_2	1,681	27,203	1,697	u
	29,659	C or CH_2				
	36,004	C or CH_2				
54, 73, 92	36,044	CH_2	2,248	35,674	2,243	m
	36,189	CH_2	2,199			m

6, 37, 48, 4, 39, 50	36,206	CH_2	3,289	37.079, 36.991	3.254, 3.269	m
9, 33, 44	36,792	CH_2	2,449	35,913	2,381	br t (5.33, 5.33)
21	37,184	CH_2	2,169	37,444	2,230	m
64, 83, 102	51,166	CH	4,082	52,920	4,348	br d (10.92)
111	51,480	CH_2	3,267	50,668	3,223	m
	53,749	C				
14	59,606	C		58,812		
65, 84, 103	61,510	CH_2	4,160	61,914	4,179	m
62, 81, 100	66,791	CH	5,364	68,205	5,139	d (3.03)
10, 32, 43	67,534	CH_2	3,699	66,510	3,807	m
57, 76, 95	69,402	CH_2	3,521	68,682	3,466	m
57, 76, 95	69,402	CH_2	3,940	68,682	3,466	m
13, 15, 17	69,872	CH_2	3,692	69,350	3,727	s
63, 82, 101	70,351	CH	5,209	71,175	5,193	br dd (11.14, 3.16)
61, 80, 99	70,641	CH	3,930	70,572	4,098	m
59, 78, 97	101,188	CH	4,628	99,518	4,768	br d (8.32)
115, 124, 133	170,239	C		169,828		
112, 127, 136	170,432	C		170,323		
121, 130, 118	170,466	C		169,905		
70, 89, 108	171,034	C		171,713		
8, 34, 45	172,063	C		172,929		
1, 41, 52	173,834	C		173,440		
20	174,088	C		172,667		
			7,283			s
69, 88, 107		NH	6,739		5,571	br d (8.93)
2, 40, 51		NH	6,984		6,558	br t (5.77, 5.77)
7, 36, 46		NH	7,230		6,803	br t (5.77, 5.77)
16		NH	3,655<br>6,449		6,552	u<br>s

HRMS (ES^+) m/z $C_{91}H_{149}N_{13}O_{37}$ requires 2018.01 $[M+H]^+$; found 2018.0274 $[M+H]^+$

Compound 7 [(9*H*-fluoren-9-yl)methyl *tert*-butyl (6-oxo-6-(prop-2-yn-1-ylamino)hexane-1,5-diyl)(*S*)-dicarbamate]: A solution of N^2-(((9*H*-fluoren-9-yl)methoxy)carbonyl)-N^6-(*tert*-butoxycarbonyl)-*L*-lysine (1 g, 2.14 mmol), 2-propynylamine (0.24 mL, 4.27 mmol), DIPEA (0.75 mL, 4.27 mmol) and HATU (0.97 g, 2.56 mmol) in anhydrous DMF (5 mL) was stirred at room temp for 2 h. The completion of reaction was verified by TLC, the solution poured into water (50 mL) and extracted with EtOAc (3x50 mL). The combined organic layers were dried over anhydrous Na_2SO_4, filtered and the filtrate evaporated under reduced pressure. The residue was purified by CombiFlash chromatography (20% EtOAc in n-hexane, R_f = 0.65) to give **7** as a yellowish oil (0.95 g, 88%).

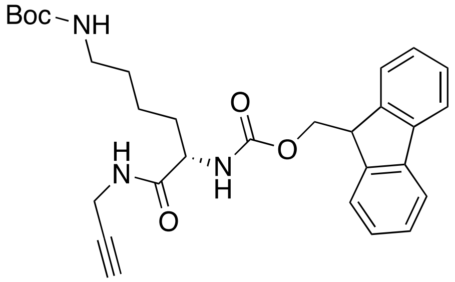

7

^{1}H NMR (300 MHz, CHLOROFORM-*d*) δ ppm: 1.30 – 1.40 (m, 2 H, -C*H*$_2$-), 1.44 (s, 9 H, 3x –CH_3), 1.48 – 1.94 (m, 4 H, 2 x -C*H*$_2$-), 2.16 - 2.23 (m, 1 H, -C-C*H*-), 3.04 – 3.19 (m, 2 H, -C*H*$_2$-NH-OCO-), 3.93 - 4.08 (m, 2 H, C-C*H*$_2$-NH-), 4.09 - 4.25 (m, 2 H, 2 x -C*H*-), 4.38 – 4.47 (m, 2 H, -OC*H*$_2$-), 4.64 (s, 1 H, -N*H*-), 5.55 (s, 1 H, -N*H*-), 6.58 (s, 1 H, -N*H*-), 7.33 (t, *J*=7.5, 2 H, 2 x C*H*$_{Ar}$), 7.41 (t, *J*=7.2 Hz, 2 H, 2 x -C*H*$_{Ar}$), 7.59 (d, *J*=7.5 Hz, 2 H, 2 x -C*H*$_{Ar}$), 7.77 (d, *J*=7.2 Hz, 2 H, 2 x -C*H*$_{Ar}$).

^{13}C NMR (75 MHz, CHLOROFORM-*d*) δ ppm: 22.39, 28.43, 29.20, 29.58, 31.88, 38.61, 47.13, 54.67, 67.05, 71.78, 79.15, 79.31, 120.00, 125.05, 127.10, 127.76, 141.31, 143.73, 156.24, 171.42.

HRMS (ES^+) m/z $C_{29}H_{35}N_3O_5$ requires 506.26 $[M+H]^+$; found 506.2699 $[M+H]^+$

Compound 8 [(9*H*-fluoren-9-yl)methyl (*S*)-(6-amino-1-oxo-1-(prop-2-yn-1-ylamino)hexan-2-yl)carbamate]: A solution of compound 6 (0.5 g, 0.99 mmol) was dissolved in CH_2Cl_2 (9 mL), TFA (1 mL) was added and stirred at room temperature for 1 h. After completion, the reaction mixture was evaporated, dissolved in CH_2Cl_2 (10 ml) and extracted with saturated aqueous Na_2CO_3 solution (10 mL). The organic layer was evaporated under reduced pressure to give compound 7 as a light yellow hygroscopic solid (0.40g, 100% w/w). This compound was used in further reactions without purification.

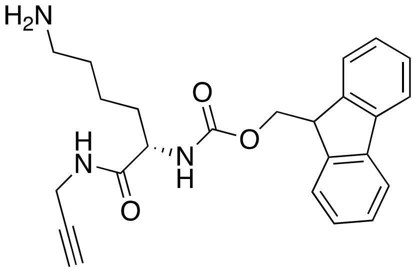

8

^{1}H NMR (300 MHz, MeOD) δ ppm: 1.26 – 1.45 (m, 2 H, -C*H₂*-), 1.50 – 1.85 (m, 4 H, 2 x -C*H₂*-), 2.50 - 2.58 (m, 1 H, -C-C*H*), 3.04 – 2.84 (t, *J*=7.55 Hz, 2 H, -C*H₂*-NH-OCO-), 3.86 – 3.96 (m, 2 H, C-C*H₂*-NH-), 3.99 - 4.06 (m, 2 H, -C*H*-), 4.11 - 4.25 (m, 1 H, -C*H*-), 4.36 (d, *J*=6.4 Hz, 2 H, -OC*H₂*-), 7.27 (t, *J*=6.8 Hz, 2 H, 2 x -CH_{Ar}), 7.34 (t, *J*=7.2 Hz, 2 H, 2 x -CH_{Ar}), 7.61 (d, *J*=7.0 Hz, 2 H, 2 x -CH_{Ar}), 7.75 (d, *J*=7.5 Hz, 2 H, 2 x -CH_{Ar}).

^{13}C NMR (75 MHz, MeOD) δ ppm: 23.89, 28.20, 29.66, 32.73, 39.03, 40.62, 56.12, 68.01, 72.44, 80.56, 121.11, 126.26, 128.32, 128.97, 142.78, 145.46, 158.64, 174.42.

HRMS (ES^+) m/z $C_{24}H_{27}N_3O_3$ requires 406.21 $[M+H]^+$; found 406.2154 $[M+H]^+$

Compound 9: A solution of compound **6** (0.3 g, 0.15 mmol), compound **8** (90 mg, 0.22 mmol) and additive tris((1-benzyl-1H-1,2,3-triazol-5-yl)methyl)amine (79 mg, 0.15 mmol) were dissolved in THF:H_2O (9:1, 1 mL), stirred at room temperature and flushed by argon. Freshly prepared hydrated solution of sodium ascorbate (7.8 mg, 45 μmol) and $CuCl_2.2H_2O$ (7.6 mg, 45 μmol) were properly mixed in closed vial. This mixture was added to reaction mixture as one lot and stirring continued at room temperature for 1 h. The reaction progress was monitored ESI-LRMS. After completion of reaction, Cu was quenched by 1M EDTA (100 μL) addition and

product was purified by RP-HPLC and lyophilized to give compound 9 as a white solid (0.2 g, 58%).

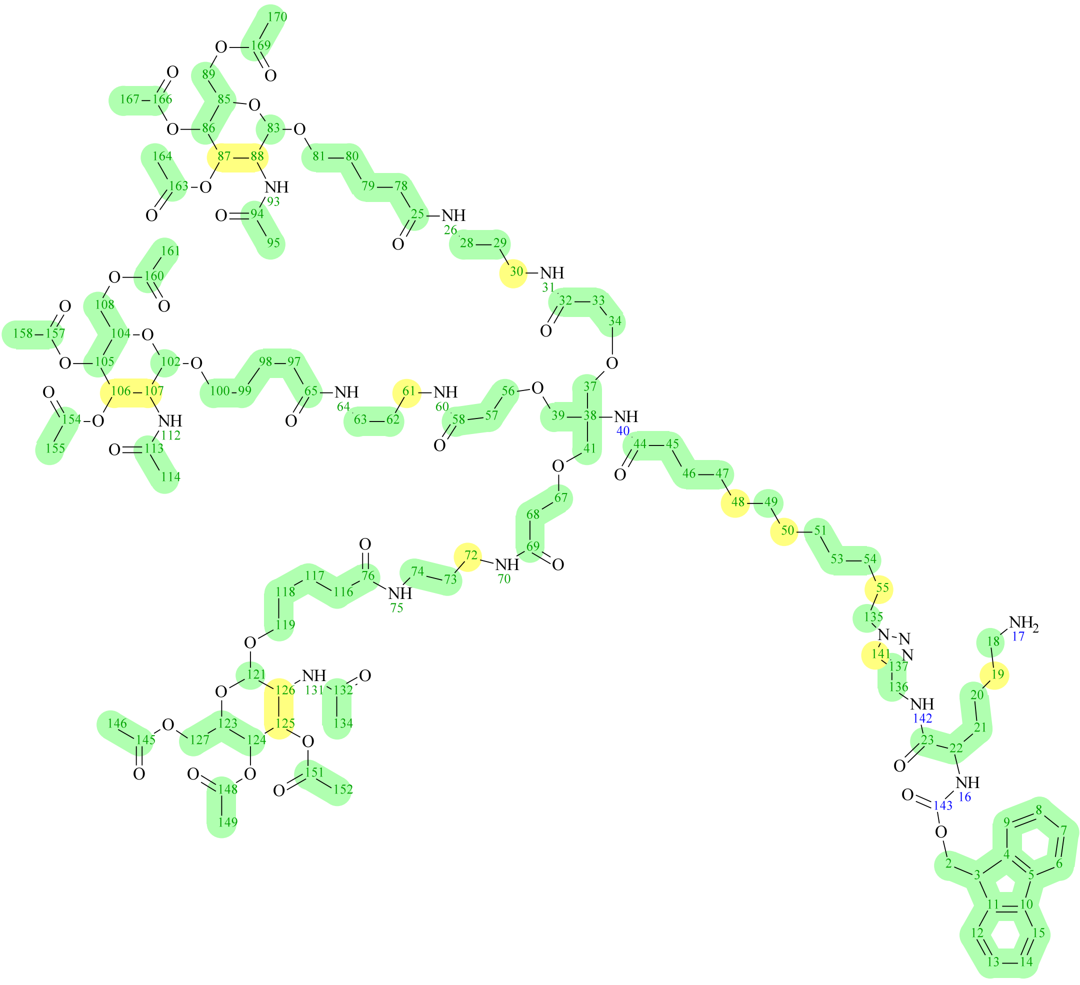

9

Atoms #	C Shift [ppm]	XHn	H Shift [ppm]	C/X Multiplicity	H Multiplicity
149, 158, 167	19,934	CH_3	1,935	s	m
152, 155, 164	19,942	CH_3	2,120	s	m
146, 161, 170	20,018	CH_3	1,992	s	m
20	22,222	CH_2	1,326	u	br dd (15.69, 8.67)
95, 114, 134	22,222	CH_3	1,868	u	m
79, 98, 117	22,222	CH_2	1,565	u	m
46	25,540	CH_2	1,461	s	m
54, 53, 51, 50, 49, 48	25,895	CH_2	1,162	s	m
19	26,209	CH_2	1,605	s	m
80, 99, 118	28,421	CH_2	1,515	s	m
30, 61, 72, 29, 62, 73	28,683	CH_2		s	
29, 62, 73	28,739	CH_2	1,617	s	m
30, 61, 72	28,739	CH_2	3,138	s	t (1.00, 1.00)
55	29,063	CH_2	1,177	s	m
47	29,063	CH_2	1,222	s	br s
21	30,916	CH_2	1,736	u	br s
21	30,916	CH_2	1,617	u	m
136	34,283	CH_2	4,394	u	br s
78, 97, 116	35,457	CH_2	2,144	s	m
33, 57, 68	36,293	CH_2		s	
33, 57, 68	36,446	CH_2	2,367	s	t (6.03, 6.03)
45	36,491	CH_2	2,097	s	m
28, 63, 74	36,584	CH_2	3,131	s	t (1.00, 1.00)
18	39,120	CH_2	2,871	s	br t (1.00, 1.00)
3	46,920	CH	4,231	u	m
88, 107, 126	50,101	CH	3,989	s	m
135	50,101	CH_2	4,246	u	m
22	54,889	CH	4,005	u	m
38	59,848	C		s	
89, 108, 127	61,818	CH_2	4,108	u	dd (1.00, 1.00)
89, 108, 127	61,818	CH_2	4,062	u	dd (1.00, 1.00)
2	66,546	CH_2	4,325	u	m
87, 106, 125	66,990	CH	5,276	s	m
34, 56, 67	67,414	CH_2	3,614	s	m
37, 39, 41	68,723	CH_2	3,587	u	br s
81, 100, 119	69,414	CH_2	3,497	s	dt (1.00, 1.00, 1.00)
81, 100, 119	69,414	CH_2	3,803	s	dt (1.00, 1.00, 1.00)
85, 104, 123	70,323	CH	3,974	s	m
86, 105, 124	70,783	CH	4,987	s	dd (11.18, 3.38)
83, 102, 121	101,078	CH	4,534	s	d (8.50)
6, 15	120,059	CH	7,826	s	br d (7.20)
141	122,989	CH	7,685	u	s

9, 12	125,239	CH		s	
93, 112, 131	125,330	NH	7,546	u	m
9, 12	125,330	CH	7,648	u	br d (7.46)
8, 13	127,268	CH	7,322	m	m
7, 14	127,862	CH		s	
7, 14	127,876	CH	7,414	s	br d (7.37)
5, 10	141,075	C		s	
4, 11	143,907	C		s	
137	144,509	C		m	
148, 157, 166	171,141	C		s	
145, 160, 169	171,354	C		s	
154, 163, 151	171,440	C		s	
94, 113, 132	172,461	C		s	
32, 58, 69	172,829	C		s	
23	173,131	C		s	
25, 65, 76	174,875	C		s	
44	175,134	C		m	
26, 64, 75		NH	7,587		m
31, 60, 70		NH	7,492		m

HRMS (ES^+) m/z $C_{115}H_{176}N_{16}O_{40}$ requires 2422.2201 $[M+H]^+$; found 2422.2303 $[M+H]^+$

Compound 10 [2,2',2''-(10-(2-(((*S*)-6-(((1-(1-(((3*R*,4*R*,5*R*,6*R*)-3-acetamido-4,5-dihydroxy-6-(hydroxymethyl)tetrahydro-2*H*-pyran-2-yl)oxy)-16,16-bis((3-((3-(5-(((3*R*,4*R*,5*R*,6*R*)-3-acetamido-4,5-dihydroxy-6-(hydroxymethyl)tetrahydro-2*H*-pyran-2-yl)oxy)pentanamido)propyl)amino)-3-oxopropoxy)methyl)-5,11,18-trioxo-14-oxa-6,10,17-triazanonacosan-29-yl)-1*H*-1,2,3-triazol-4-yl)methyl)amino)-5-amino-6-oxohexyl)amino)-2-oxoethyl)-1,4,7,10-tetraazacyclododecane-1,4,7-triyl)triacetic acid]: Compound **9** (0.15 g, 0.06 mmol) was dissolved in DMF (0.5 mL), added to 1M $NaHCO_3$ solution (2 mL) and stirred at room temperature. DOTA-NHS ester (94.0 mg, 0.12 mmol) was dissolved in DMF (0.5 mL), added dropwise to reaction mixture and stirred for 1 h at room temperature. The reaction progress was monitored ESI-LRMS. The residue was purified by RP-HPLC and lyophilized. The lyophilized product was further dissolved in MeOH (2 mL) and NaOMe (39.0 mg, 0.73 mmol) was added to the reaction mixture. The completion of reaction was confirmed by ESI-LRMS. Solvent was evaporated under reduced pressure and the residue was dissolved in DMF (2 mL), diethylamine (1 mL) was added and stirred at room temperature for 1 h. The completion of

reaction was confirmed by ESI-LRMS and the final product was purified by RP-HPLC and lyophilized to give compound **10** as a white solid (22 mg, 16%).

^{1}H NMR (700 MHz, DMSO-d$_6$) δ ppm: 1.03 - 1.09 (m, 2 H) 1.22 (s, 27 H) 1.24 - 1.30 (m, 6 H) 1.25 - 1.32 (m, 4 H) 1.39 - 1.43 (m, 3 H) 1.39 - 1.45 (m, 18 H) 1.41 - 1.44 (m, 1 H) 1.48 - 1.52 (m, 20 H) 1.68 - 1.73 (m, 4 H) 1.73 - 1.73 (m, 1 H) 1.75 - 1.80 (m, 7 H) 1.80 - 1.81 (m, 11 H) 1.81 - 1.81 (m, 1 H) 1.81 (s, 15 H) 1.87 (s, 1 H) 1.89 - 1.90 (m, 1 H) 2.03 - 2.04 (m, 1 H) 2.05 (br t, *J*=7.37 Hz, 14 H) 2.04 - 2.07 (m, 2 H) 2.07 (s, 3 H) 2.99 - 3.00 (m, 2 H) 3.04 (br s, 34 H) 3.04 - 3.07 (m, 10 H) 3.16 - 3.30 (m, 66 H) 3.30 (br d, *J*=6.33 Hz, 31 H) 3.34 - 3.38 (m, 246 H) 3.39 - 3.45 (m, 1 H) 3.40 (br s, 30 H) 3.41 - 3.44 (m, 17 H) 3.58 (br dd, *J*=10.71, 2.90 Hz, 10 H) 3.57 - 3.59 (m, 1 H) 3.60 - 3.63 (m, 10 H) 3.66 - 3.76 (m, 11 H) 3.69 (br s, 4 H) 3.68 - 3.72 (m, 1 H) 3.69 - 3.72 (m, 1 H) 3.72 (br s, 2 H) 3.74 (br d, *J*=6.16 Hz, 4 H) 3.86 (br d, *J*=11.10 Hz, 3 H) 4.05 (dd, *J*=11.70, 1.73 Hz, 2 H) 4.03 - 4.06 (m, 1 H) 4.13 (d, *J*=2.86 Hz, 2 H) 4.33 - 4.35 (m, 1 H) 4.33 - 4.35 (m, 2 H) 4.37 (br d, *J*=5.29 Hz, 2 H) 4.39 - 4.41 (m, 2 H) 4.42 - 4.43 (m, 1 H) 6.55 (br s, 18 H) 6.94 - 6.97 (m, 1 H) 6.99 (s, 2 H) 7.38 - 7.57 (m, 2 H) 7.64 (s, 2 H) 7.66 - 7.68 (m, 2 H) 7.75 (s, 1 H) 7.85 (t, *J*=5.55 Hz, 6 H) 7.97 (s, 2 H) 8.31 (s, 2 H) 8.45 - 8.46 (m, 1 H) 8.87 - 9.01 (m, 1 H) 11.85 - 11.91 (m, 1 H).

^{13}C NMR (176 MHz, DMSO-*d*$_6$) δ ppm: 1.61, 15.22, 19.63, 22.33, 22.43, 25.77, 26.35, 29.04, 28.91, 29.09, 29.38, 29.41, 29.46, 29.78, 31.08, 35.57, 36.47, 36.75, 36.84, 39.33, 40.17, 40.43, 49.78, 52.51, 59.95, 60.94, 67.78, 67.98, 68.22 - 68.29 (m, 1 C), 68.37, 68.68, 68.71, 70.58, 71.97, 75.70, 79.62, 84.48, 101.56, 101.85, 110.63, 115.16, 116.86, 118.57, 123.32, 132.11, 144.14, 158.29, 158.47, 161.68, 165.52, 167.82, 168.90, 170.11, 170.66, 172.62, 177.67.

HRMS (ES$^+$) m/z $C_{98}H_{174}N_{20}O_{36}$ requires 1104.61 $[M-H]^{2+}$; found 1104.6168 $[M-H]^{2+}$

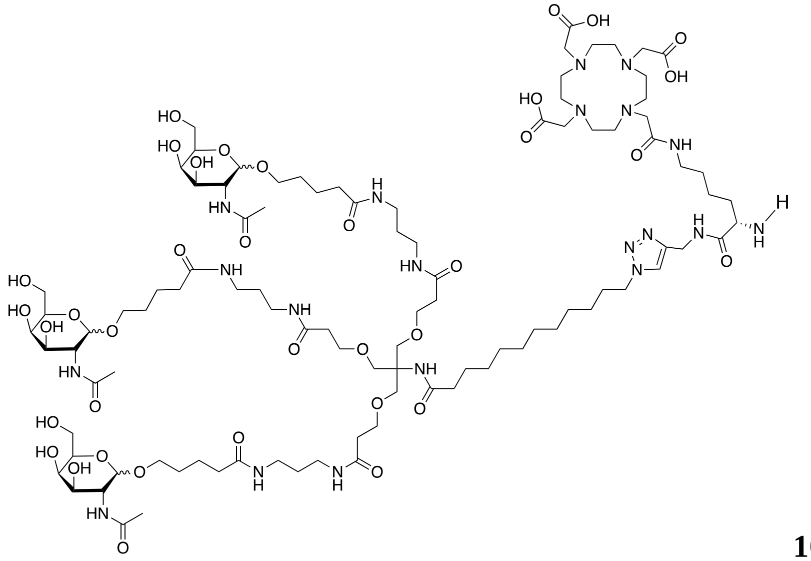

10

Compound 11 [3-(6-((((*S*)-1-(((1-(1-(((3*R*,4*R*,5*R*,6*R*)-3-acetamido-4,5-dihydroxy-6-(hydroxymethyl)tetrahydro-2*H*-pyran-2-yl)oxy)-16,16-bis((3-((3-(5-(((3*R*,4*R*,5*R*,6*R*)-3-acetamido-4,5-dihydroxy-6-(hydroxymethyl)tetrahydro-2*H*-pyran-2-yl)oxy)pentanamido)propyl)amino)-3-oxopropoxy)methyl)-5,11,18-trioxo-14-oxa-6,10,17-triazanonacosan-29-yl)-1*H*-1,2,3-triazol-4-yl)methyl)amino)-1-oxo-6-(2-(4,7,10-tris(carboxymethyl)-1,4,7,10-tetraazacyclododecan-1-yl)acetamido)hexan-2-yl)amino)-6-oxohexyl)-1,1-dimethyl-2-((1*E*,3*E*,5*Z*)-5-(1,1,3-trimethyl-1,3-dihydro-2*H*-benzo[*e*]indol-2-ylidene)penta-1,3-dien-1-yl)-1*H*-benzo[*e*]indol-3-ium]: Compound **10** (6.0 mg, 2.7 μmol) was dissolved in 1 M phosphate buffer (0.5 mL). Cy 5.5-NHS ester (2.5 mg, 3.2 μmol) in DMF (0.5 mL) was added dropwise to reaction and mixture was stirred for 1 h at room temperature. The completion of reaction was confirmed by ESI-LRMS. The product was purified by RP-HPLC and lyophilized to give compound **11** as a blue solid (1.8 mg, 23%).

HRMS (ES$^+$) m/z $C_{138}H_{215}N_{22}O_{37}^+$ requires 1386.6754 [M-H]$^{2+}$; found 1386.6431 [M-H]$^{2+}$. RP-HPLC: 97.8% purity. $\lambda_{max}/\lambda_{em}$ (H_2O) 640/680 nm. Molar extinction coefficient = 188,000 M^{-1} cm^{-1}.

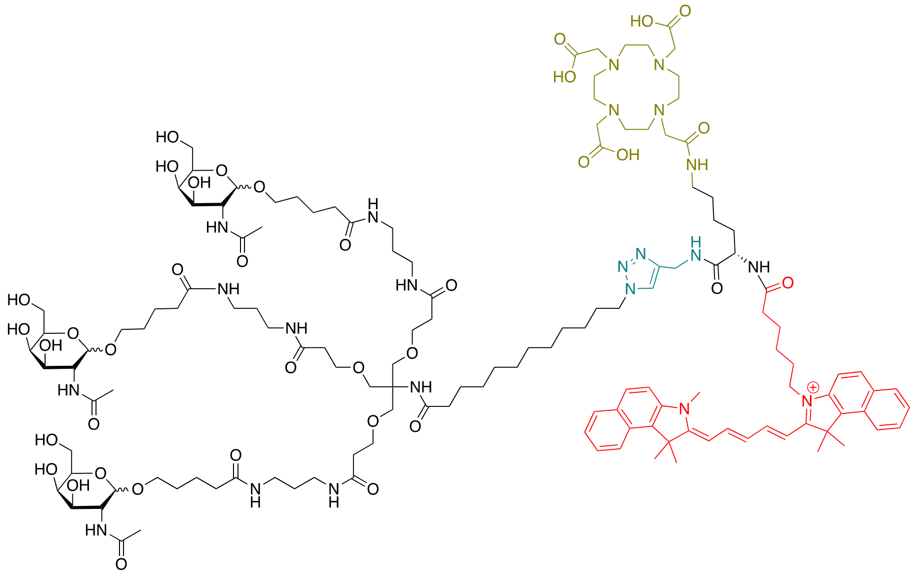

11

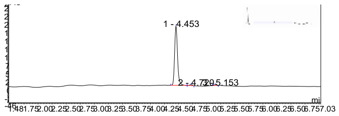

Compound 12 [benzyl N^6-(((9*H*-fluoren-9-yl)methoxy)carbonyl)-N^2-(*tert*-butoxycarbonyl)-*L*-lysinate]: A solution of N^2-(((9*H*-fluoren-9-yl)methoxy)carbonyl)-N^6-(*tert*-butoxycarbonyl)-*L*-lysine (1 g, 2.14 mmol), benzyl alcohol (0.46 g, 4.27 mmol), DIPEA (0.75 mL, 4.27 mmol) and HATU (0.97 g, 2.56 mmol) in anhydrous DMF (5 mL) was stirred at room temp for 2 h. The completion of reaction was verified by TLC, the solution poured into water (50 mL) and extracted with EtOAc (3x50 mL). The combined organic layers were dried over anhydrous Na_2SO_4, filtered and the filtrate evaporated under reduced pressure. The residue was purified by CombiFlash high performance flash chromatography (20% EtOAc in n-hexane, R_f = 0.65) to give **12** as a yellowish oil (1.05 g, 88%).

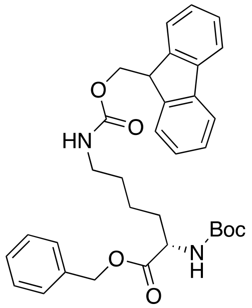

12

^{1}H NMR (300 MHz, CHLOROFORM-*d*) δ ppm: 1.31 – 1.40 (m, 2 H, -CH_2-), 1.45 (s, 9 H, 3x –CH_3), 1.48 – 1.91 (m, 4 H, 2 x -CH_2-), 2.56 – 2.79 (m, 2 H, -CH_2-NH-OCO-), 2.88 - 3.31 (m, 2 H, C-CH_2-NH-), 3.91 - 4.18 (m, 1 H, - C*H*-), 4.20 – 4.48 (m, 1 H, -C*H*-), 5.15 (s, 2 H, -OCH_2-C_6H_5), 6.09 (s, 1 H, -N*H*-), 7.07 (s, 1 H, -N*H*-), 7.28 - 7.46 (m, 9 H, 9 x -CH_{Ar}), 7.60 (d, *J*=7.2 Hz, 2 H, 2 x -CH_{Ar}), 7.78 (d, *J*=7.2 Hz, 2 H, 2 x -CH_{Ar}).

^{13}C NMR (75 MHz, CHLOROFORM-*d*) δ ppm: 22.45, 28.28, 28.46, 32.43, 46.29, 52.95, 54.13, 65.18, 66.92, 79.41, 119.70, 120.96, 124.30, 126.62, 126.92, 127.01, 127.54, 128.29, 128.55, 135.40, 154.52, 155.77, 170.12.

HRMS (ES^+) m/z $C_{33}H_{38}N_2O_6$ requires 559.58 $[M+H]^+$; found 559.2822 $[M+H]^+$.

Compound 13 [benzyl *N*^{6}-(((9*H*-fluoren-9-yl)methoxy)carbonyl)-*L*-lysinate]: A solution of compound **12** (1.00 g, 1.79 mmol) was dissolved in CH_2Cl_2 (9 mL), TFA (1 mL) was added and stirred at room temperature for 1 h. After completion, the reaction mixture was evaporated, dissolved in CH_2Cl_2 (10 ml) and extracted with saturated aqueous Na_2CO_3 solution (10 mL). The organic layer was evaporated under reduced pressure. The residue was purified by CombiFlash high performance flash chromatography (5% MeOH in CH_2Cl_2, R_f = 0.3) to give **13** as a yellowish oil (0.56 g, 68%).

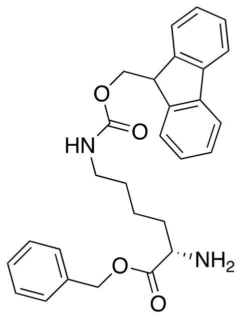

13

^{1}H NMR (300 MHz, CHLOROFORM-*d*) δ ppm: 1.20 – 1.50 (m, 4 H, 2 x -CH_2-), 1.78 – 2.02 (m, 2 H, -CH_2-), 2.88 – 3.11 (m, 2 H, -CH_2-NH-OCO-), 3.97 - 4.10 (m, 1 H, -C*H*-), 4.12 – 4.24 (m, 1 H, -C*H*-), 4.29 – 4.48 (m, 2 H, C-CH_2-NH-), 5.19 (s, 2 H, -OCH_2-C_6H_5), 7.23 - 7.44 (m, 9 H, 9 x -CH_{Ar}), 7.55 (d, *J*=7.2 Hz, 2 H, 2 x -CH_{Ar}), 7.75 (d, *J*=7.5 Hz, 2 H, 2 x -CH_{Ar}), 7.97 (br. s, 3 H, -N*H*-, -NH_2).

^{13}C NMR (75 MHz, CHLOROFORM-*d*) δ ppm: 21.28, 28.93, 29.50, 46.99, 51.45, 53.07, 68.65, 69.35, 119.98, 124.91, 127.08, 127.76, 128.62, 128.73, 128.99, 134.07, 141.29, 143.64, 161.26, 172.0.

HRMS (ES^+) m/z $C_{28}H_{30}N_2O_4$ requires 459.22 $[M+H]^+$; found 459.2321 $[M+H]^+$.

Compound 14 [methyl (S)-16-((6-((((9H-fluoren-9-yl)methoxy)carbonyl)amino)-1-(benzyloxy)-1-oxohexan-2-yl)amino)-16-oxohexadecanoate]: A solution of compound **13** (0.25 g, 0.56 mmol), 16-methoxy-16-oxohexadecanoic acid (0.20 g, 0.66 mmol), DIPEA (0.2 mL, 1.1 mmol) and HATU (0.25 g, 0.66 mmol) in anhydrous DMF (5 mL) was stirred at room temp for overnight. The completion of reaction was verified by ESI-LRMS, the solvent was evaporated under reduced pressure and purified by CombiFlash high performance flash chromatography (5% MeOH in CH_2Cl_2, R_f = 0.35, 0.31 g, 73%).

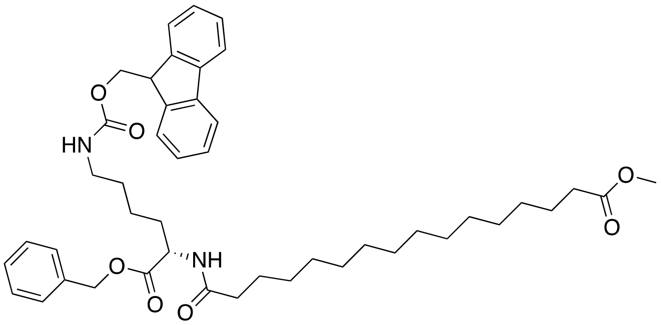

14

^{1}H NMR (300 MHz, CHLOROFORM-*d*) δ ppm: 1.22 – 1.36 (m, 22 H, 11 x -C*H$_2$*-), 1.54 – 1.69 (m, 8 H, 4 x -C*H$_2$*-), 2.21 (t, *J*=7.2 Hz, 2 H, -NHCO-C*H$_2$*-), 2.26 – 2.36 (m, 2 H, -C*H$_2$*-COOCH$_3$), 3.05 – 3.23 (m, 2 H, -C*H$_2$*-NH-OCO-), 3.67 (s, 3 H, -C*H$_3$*), 4.15 - 4.30 (m, 1 H, - C*H*-), 4.34 – 4.51 (m, 2 H, C-C*H$_2$*-NH), 4.60 – 4.74 (m, 1 H, -C*H*-), 4.70 (s, 1 H, -N*H*-), 5.19 (s, 2 H, -OC*H$_2$*-C$_6$H$_5$), 6.04 (s, 1 H, -N*H*-), 7.28 - 7.37 (m, 7 H, 7 x -C*H*$_{Ar}$), 7.41 (t, *J*=7.5 Hz, 2 H, 2 x -C*H*$_{Ar}$), 7.60 (d, *J*=7.5 Hz, 2 H, 2 x -C*H*$_{Ar}$), 7.77 (d, *J*=7.5 Hz, 2 H, 2 x -C*H*$_{Ar}$).

HRMS (ES$^+$) m/z $C_{45}H_{60}N_2O_7$ requires 741.44 [M+H]$^+$; found 741.4522 [M+H]$^+$.

Compound 15 [*N*^{6}-(((9*H*-fluoren-9-yl)methoxy)carbonyl)-*N*^{2}-(16-methoxy-16-oxohexadecanoyl)-*L*-lysine]: A solution of compound 14 (0.30 g, 0.41 mmol), (10%) Pd-C (1/10, w/w) in THF (5 mL) under H$_2$ (20 psi) was stirred at room temperature in a Parr apparatus for 6 h. The reaction mixture was filtered through Celite, the filtrate evaporated under reduced pressure. The residue was purified by RP-HPLC and lyophilized to give compound **15** as a colorless oil (0.16 g, 61%).

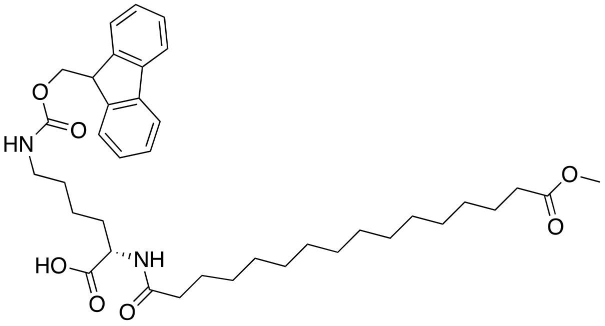

15

^{1}H NMR (300 MHz, CHLOROFORM-*d*) δ ppm: 1.21 – 1.32 (m, 24 H, 12 x -C*H$_2$*-), 1.53 – 1.71 (m, 6 H, 3 x -C*H$_2$*-), 2.21 (t, *J*=7.2 Hz, 2 H, -NHCO-C*H$_2$*-), 2.30 – 2.36 (m, 2 H, -C*H$_2$*-COOCH$_3$), 3.08 – 3.33 (m, 2 H, -C*H$_2$*-NH-OCO-), 3.68 (s, 3 H, -C*H$_3$*), 4.15 - 4.30 (m, 1 H, - C*H*-

), 4.30 – 4.75 (m, 3 H, -C-CH_2-NH, -C*H*-), 6.46 (s, 1 H, -N*H*-), 7.31 - 7.37 (m, 2 H, 2 x -CH_{Ar}), 7.41 (t, *J*=7.5 Hz, 2 H, 2 x -CH_{Ar}), 7.59 (d, *J*=7.5 Hz, 2 H, 2 x -CH_{Ar}), 7.78 (d, *J*=7.5 Hz, 2 H, 2 x -CH_{Ar}).

^{13}C NMR (75 MHz, CHLOROFORM-*d*) δ ppm: 21.18, 23.11, 23.63, 29.13, 29.23, 29.41, 29.56, 30.30, 34.22, 35.34, 36.49, 38.82, 41.53, 47.49, 54.08, 57.96, 65.95, 120.02, 125.52, 128.24, 135.74, 143.38, 145.15, 155.64, 174.77, 176.54, 177.81.

HRMS (ES^+) m/z $C_{38}H_{54}N_2O_7$ requires 651.39 $[M+H]^+$; found 651.4031 $[M+H]^+$.

Compound 16 [(*tert*-butyl (*S*)-(5-amino-6-oxo-6-(prop-2-yn-1-ylamino)hexyl)carbamate]: A solution of compound **7** (0.40 g, 0.80 mmol) was dissolved in CH_2Cl_2 (4 mL), diethylamine (2 mL) was added and stirred at room temperature for 1 h. After completion, the reaction mixture was evaporated, dissolved in CH_2Cl_2 (10 ml) and extracted with 1M HCl solution (10 mL). The organic layer was evaporated under reduced pressure and purified by CombiFlash high performance flash chromatography (10% MeOH in CH_2Cl_2, R_f = 0.3) to give compound **16** as colorless oil (150 mg, 68%).

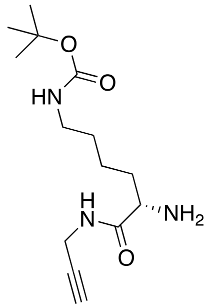

16

^{1}H NMR (300 MHz, CHLOROFORM-*d*) δ ppm: 1.34 – 1.41 (m, 2 H, -CH_2-), 1.43 (s, 9 H, 3x –CH_3), 1.61 – 1.73 (m, 64 H, 2 x -CH_2-), 2.19 - 2.29 (m, 1 H, -C-C*H*-), 3.10 (s, 1 H, -NH_2), 3.35 – 3.52 (m, 1 H, -C*H*-NH_2), 3.99 - 4.10 (m, 2 H, -C-CH_2-NH), 4.64 (s, 1 H, -N*H*-), 7.65 (s, 1 H, -N*H*).

^{13}C NMR (75 MHz, CHLOROFORM-*d*) δ ppm: 22.50, 28.40, 28.78, 29.76, 34.25, 44.72, 54.73, 71.31, 79.05, 79.66, 156.19, 171.15.

HRMS (ES^+) m/z $C_{14}H_{25}N_3O_3$ requires 284.19 $[M+H]^+$; found 284.1980 $[M+H]^+$

Compound 17 [methyl 16-(((9*S*,12*S*)-12-(4-aminobutyl)-1-(9*H*-fluoren-9-yl)-3,10,13-trioxo-2-oxa-4,11,14-triazaheptadec-16-yn-9-yl)amino)-16-oxohexadecanoate]: A solution of compound **15** (150 mg, 0.23 mmol), compound **16** (74.0 mg, 0.26 mmol), DIPEA (0.09 mL, 0.52 mmol) and HATU (0.10 g, 0.26 mmol) in anhydrous DMF (2 mL) was stirred at room temp for overnight. The completion of reaction was verified by ESI-LRMS, the solvent was evaporated under reduced pressure, dissolved in CH_2Cl_2 (5 mL), TFA (1 mL) was added in solution and it was stirred at room temperature for 30 minutes. The completion of reaction was verified by ESI-LRMS, the product was purified by RP-HPLC and lyophilized to give compound **17** as a colorless oil (0.1 g, 48%).

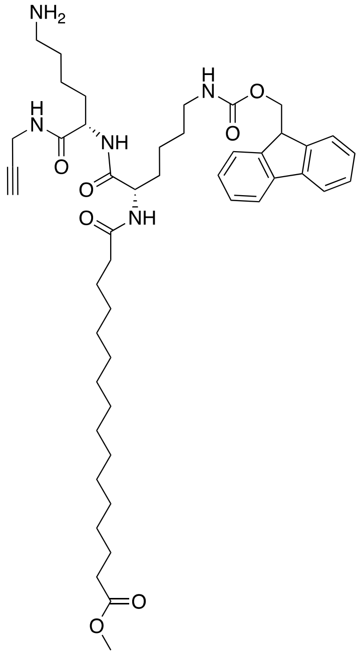

17

^{1}H NMR (300 MHz, MeOD) δ ppm: 1.18 – 1.93 (m, 38 H, 19 x -C*H*$_2$-), 2.10 – 2.22 (m, 2 H, -C*H*$_2$-COOCH$_3$), 2.25 (t, *J*=7.2 Hz, 2 H, -NHCO-C*H*$_2$-), 2.49 – 2.59 (m, 1 H, -C-C*H*-), 2.76 – 2.95 (m, 2 H, -C*H*$_2$-NHCOO-), 3.00 – 3.14 (m, 2 H, -C*H*$_2$-NH$_2$), 3.60 (s, 3 H, -C*H*$_3$), 3.85 – 3.97 (m, 2 H, -C-C*H*$_2$-NHCO-), 4.07 - 4.22 (m, 1 H, -C*H*-), 4.25 – 4.35 (m, 2 H, -NHCOO-C*H*$_2$-), 7.28 (t, *J*=7.4 Hz, 2 H, 2 x -C*H*$_{Ar}$), 7.35 (t, *J*=7.4 Hz, 2 H, 2 x -C*H*$_{Ar}$), 7.60 (d, *J*=7.5 Hz, 2 H, 2 x -C*H*$_{Ar}$), 7.76 (d, *J*=7.2 Hz, 2 H, 2 x -C*H*$_{Ar}$).

^{13}C NMR (75 MHz, MeOD) δ ppm: 23.74, 24.25, 26.17, 27.07, 28.07, 29.66, 30.33, 30.53, 30.89, 34.95, 36.87, 40.67, 41.46, 46.44, 52.12, 55.33, 57.35, 67.85, 72.55, 80.54, 121.14, 126.31, 128.31, 128.98, 142.77, 145.49, 150.09, 173.67, 175.00, 176.21, 177.12.

HRMS (ES$^+$) m/z $C_{47}H_{69}N_5O_7$ requires 816.49 $[M+H]^+$; found 816.5293 $[M+H]^+$.

Compound 18: A solution of compound **6** (200.0 mg, 0.099 mmol), compound **17** (0.12 g, 0.15 mmol) and additive tris((1-benzyl-1H-1,2,3-triazol-5-yl)methyl)amine (52.0 mg, 0.099 mmol) were dissolved in THF:H_2O (9:1, 1 mL), stirred at room temperature and flushed by argon. Freshly prepared hydrated solution of sodium ascorbate (5.20 mg, 0.03 mmol) and $CuCl_2.2H_2O$ (5.00 mg, 0.03 mmol) were properly mixed in closed vial. This mixture was added to reaction mixture as one lot and stirring continued at room temperature for 1 h. The reaction progress was monitored ESI-LRMS. After completion of reaction, Cu was quenched by 1M EDTA (100 µL) and product was purified by RP-HPLC and lyophilized to give compound 18 as a white solid (0.13 g, 48%).

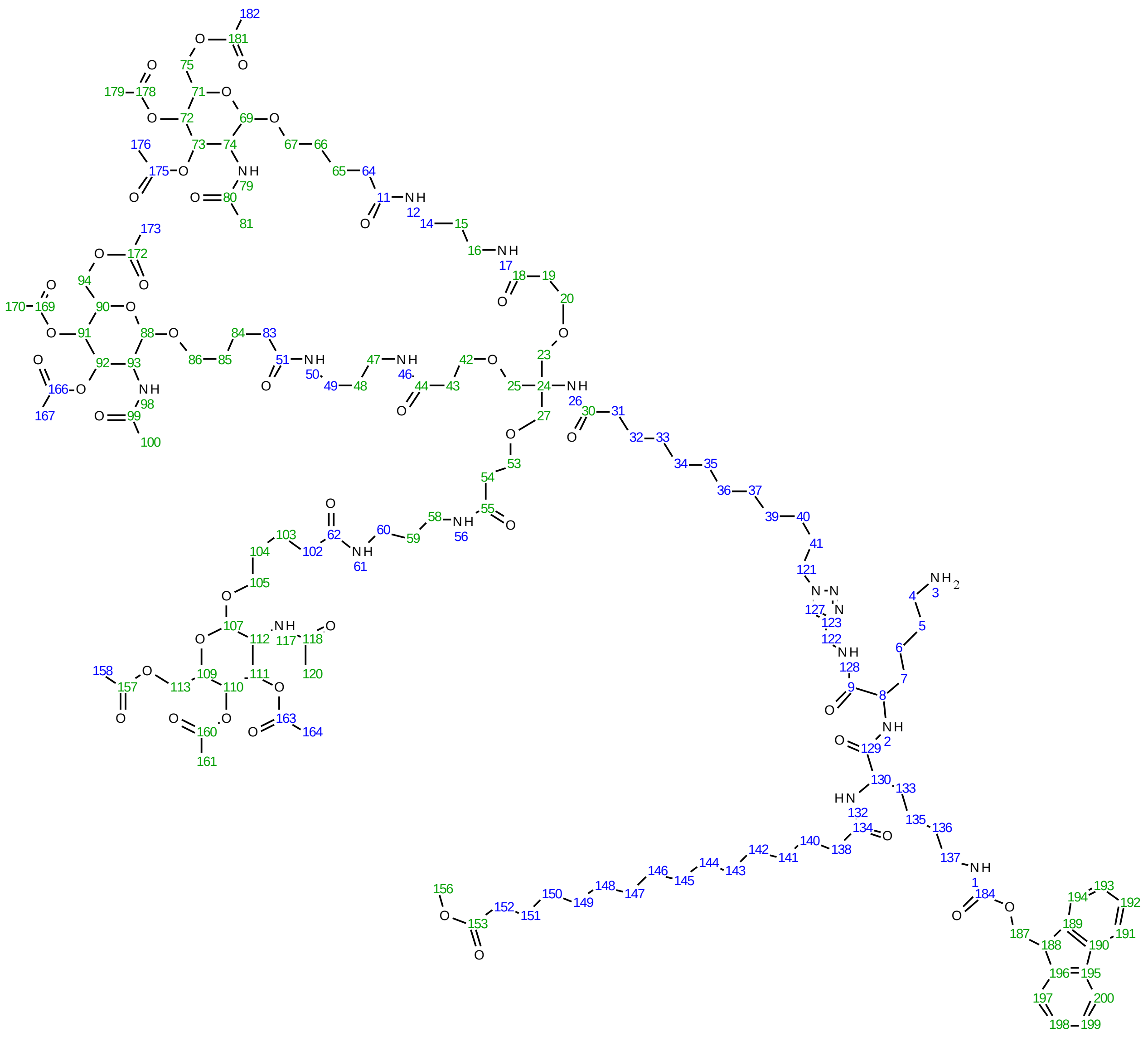

18

^{1}H NMR (700 MHz, CHLOROFORM-*d*) δ ppm: 0.03 - 0.08 (m, 1 H) 0.50 - 0.54 (m, 1 H) 0.84 - 0.95 (m, 2 H) 1.24 (br s, 42 H) 1.57 (br dd, *J*=18.90, 6.07 Hz, 10 H) 1.63 (quin, *J*=1.00 Hz, 4 H) 1.60 - 1.65 (m, 1 H) 1.66 - 1.75 (m, 26 H) 1.81 (br s, 3 H) 1.86 (br d, *J*=6.42 Hz, 4 H) 1.97 (s, 8 H) 1.99 - 2.02 (m, 14 H) 2.04 - 2.11 (m, 17 H) 2.15 (s, 12 H) 2.17 - 2.29 (m, 11 H) 2.31 (t, *J*=7.54 Hz, 3 H) 2.46 (t, *J*=1.00 Hz, 12 H) 3.11 - 3.23 (m, 6 H) 3.28 (br s, 20 H) 3.39 - 3.55 (m, 8 H) 3.63 - 3.67 (m, 4 H) 4.07 - 4.10 (m, 1 H) 4.10 - 4.23 (m, 2 H) 4.13 - 4.14 (m, 3 H) 4.17 - 4.21 (m, 3 H) 4.28 - 4.40 (m, 10 H) 4.41 - 4.48 (m, 5 H) 4.53 (br d, *J*=7.63 Hz, 1 H) 4.62 (br d, *J*=8.32 Hz, 3 H) 5.21 (br d, *J*=11.18 Hz, 14 H) 5.37 (br d, *J*=2.86 Hz, 3 H) 6.69 (br s, 2 H) 6.98 - 7.06

(m, 3 H) 7.28 - 7.29 (m, 2 H) 7.29 - 7.34 (m, 3 H) 7.62 - 7.66 (m, 1 H) 7.67 (br s, 1 H) 7.77 (br d, *J*=7.54 Hz, 6 H) 8.16 (br s, 4 H).

^{13}C NMR (176 MHz, CHLOROFORM-*d*) δ ppm: 20.62, 22.62, 23.07, 24.94, 25.72, 25.74, 26.12, 28.58, 29.31 (br t, *J*=65.25 Hz, 1 C), 28.98, 29.01, 29.12, 29.38 (br dd, *J*=33.70, 20.98 Hz, 1 C), 29.56, 29.61, 29.62, 36.34, 36.41, 36.65, 47.24, 51.38, 59.83, 66.76, 67.46, 69.40, 69.74, 70.33, 70.65, 76.99, 77.57 (br dd, *J*=17.64, 7.95 Hz, 1 C), 77.67, 77.74, 77.80, 101.20, 119.99, 125.05, 127.07, 127.74, 141.29, 143.93, 170.25, 170.46, 171.61, 172.43, 174.36, 174.40.

HRMS (ES^+) m/z $C_{138}H_{218}N_{18}O_{44}$ requires 2834.18 $[M+H]^+$; found 2834.1824 $[M+H]^+$

Compound 19 [2,2',2''-(10-(2-(((*S*)-6-(((1-(1-(((3*R*,4*R*,5*R*,6*R*)-3-acetamido-4,5-dihydroxy-6-(hydroxymethyl)tetrahydro-2*H*-pyran-2-yl)oxy)-16,16-bis((3-((3-(5-(((3*R*,4*R*,5*R*,6*R*)-3-acetamido-4,5-dihydroxy-6-(hydroxymethyl)tetrahydro-2*H*-pyran-2-yl)oxy)pentanamido)propyl)amino)-3-oxopropoxy)methyl)-5,11,18-trioxo-14-oxa-6,10,17-triazanonacosan-29-yl)-1*H*-1,2,3-triazol-4-yl)methyl)amino)-5-((*S*)-6-amino-2-(15-carboxypentadecanamido)hexanamido)-6-oxohexyl)amino)-2-oxoethyl)-1,4,7,10-tetraazacyclododecane-1,4,7-triyl)triacetic acid]: Compound **18** (50.0 mg, 0.018 mmol) was dissolved in DMF (0.5 mL), added to 1M $NaHCO_3$ solution (2 mL) and stirred at room temperature. DOTA-NHS ester (20.0 mg, 0.026 mmol) was dissolved in DMF (0.5 mL), added dropwise to reaction mixture and stirred for 1 h at room temperature. The completion of reaction was confirmed by ESI-LRMS. The solution was lyophilized, dissolved in 1 M phosphate buffer, added porcine liver esterase (100 µL) and stirred at room temperature for 2 h. The completion of demethylation at palmitic acid was confirmed by ESI-LRMS and solution was lyophilized overnight. The lyophilized residue was further dissolved in MeOH (2 mL) and NaOAc (12.0 mg, 0.22 mmol) was added to the reaction mixture. The completion of deacylation at GalNAc was confirmed by ESI-LRMS. Finally, solvent was evaporated under reduced pressure and the residue was dissolved in DMF (2 mL), diethylamine (1 mL) was added and stirred at room temperature for 1 h. The completion of reaction was confirmed by ESI-LRMS and the final product was purified by RP-HPLC and lyophilized to give compound **19** as a white solid (13 mg, 28%).

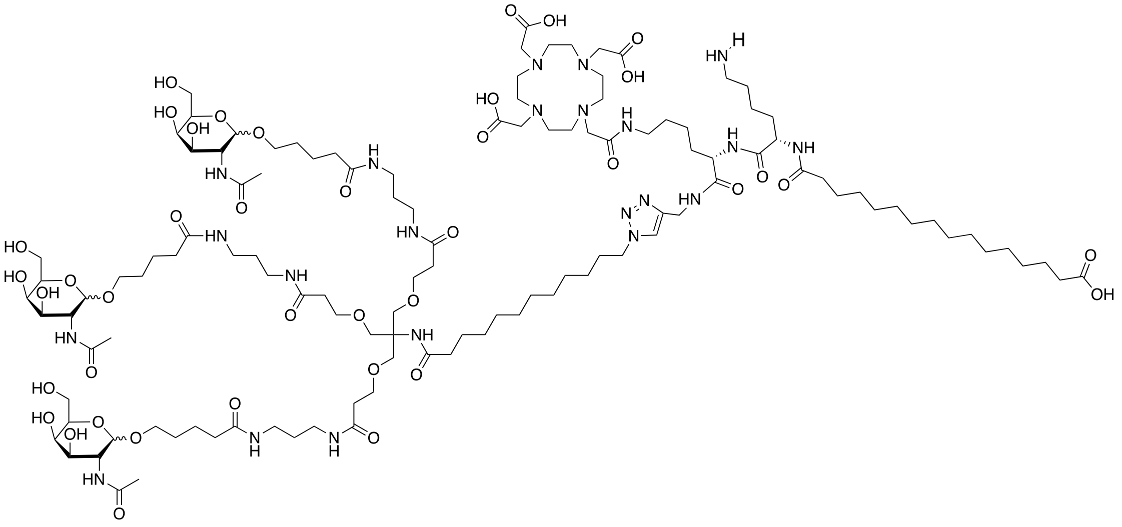

19

^{1}H NMR (700 MHz, DMSO-d_6) δ ppm: 1.20 - 1.23 (m, 3 H) 1.22 (s, 9 H) 1.21 - 1.23 (m, 2 H) 1.24 - 1.30 (m, 2 H) 1.24 - 1.29 (m, 4 H) 1.40 - 1.43 (m, 9 H) 1.41 - 1.44 (m, 2 H) 1.41 - 1.43 (m, 1 H) 1.48 - 1.51 (m, 2 H) 1.49 - 1.52 (m, 13 H) 1.64 - 1.74 (m, 4 H) 1.76 - 1.80 (m, 3 H) 1.77 - 1.81 (m, 9 H) 1.81 - 1.84 (m, 3 H) 2.05 (br t, *J*=7.37 Hz, 8 H) 2.03 - 2.07 (m, 2 H) 2.07 (s, 4 H) 2.41 (s, 1 H) 2.59 - 2.62 (m, 1 H) 3.02 - 3.05 (m, 17 H) 3.05 - 3.05 (m, 1 H) 3.07 (br d, *J*=5.72 Hz, 13 H) 3.30 (br d, *J*=6.24 Hz, 15 H) 3.33 - 3.37 (m, 116 H) 3.44 - 3.44 (m, 3 H) 3.48 (br d, *J*=6.24 Hz, 25 H) 3.50 (br d, *J*=6.24 Hz, 12 H) 3.51 - 3.54 (m, 7 H) 3.51 - 3.53 (m, 11 H) 3.62 - 3.68 (m, 4 H) 3.68 - 3.71 (m, 4 H) 3.68 - 3.76 (m, 7 H) 3.69 - 3.73 (m, 1 H) 3.86 (br d, *J*=11.10 Hz, 2 H) 4.03 - 4.06 (m, 1 H) 4.10 - 4.13 (m, 1 H) 4.14 (br d, *J*=2.86 Hz, 1 H) 4.22 (d, *J*=8.41 Hz, 3 H) 4.30 - 4.33 (m, 2 H) 4.33 - 4.34 (m, 2 H) 4.35 - 4.37 (m, 1 H) 4.41 (br d, *J*=5.55 Hz, 1 H) 4.41 - 4.43 (m, 1 H) 4.43 - 4.44 (m, 1 H) 6.99 (s, 1 H) 7.01 - 7.02 (m, 1 H) 7.64 - 7.64 (m, 1 H) 7.65 - 7.68 (m, 1 H) 7.75 (br t, *J*=5.55 Hz, 3 H) 7.85 (br t, *J*=5.55 Hz, 4 H) 7.97 (s, 1 H) 8.31 (s, 1 H) 8.31 - 8.32 (m, 1 H) 8.91 (br t, *J*=5.33 Hz, 1 H)

^{13}C NMR (176 MHz, DMSO-d_6) δ ppm: 1.17, 22.00, 23.05, 25.91, 28.46, 28.66, 28.95 (br dd, *J*=13.43, 8.03 Hz, 1 C), 29.35, 29.83, 35.14, 36.31, 36.40, 38.98, 39.01, 39.63, 39.75, 39.87, 40.01, 40.14, 52.09, 59.47 - 59.57 (m, 1 C), 60.51, 67.35, 67.55, 67.93, 71.53, 75.27, 79.18, 101.41, 116.23 (br t, *J*=12.56 Hz, 1 C), 117.94, 118.03 - 118.22 (m, 1 C), 143.54 - 143.64 (m, 1 C), 157.81, 157.99, 169.66, 170.21, 172.18, 172.61 - 172.66 (m, 1 C).

HRMS (ES$^+$) m/z $C_{120}H_{214}N_{22}O_{40}$ requires 1302.76 $[M]^{2+}$; found 1302.7664 $[M]^{2+}$.

Compound 20 [3-(6-(((*S*)-6-(((*S*)-1-(((1-(1-(((3*R*,4*R*,5*R*,6*R*)-3-acetamido-4,5-dihydroxy-6-(hydroxymethyl)tetrahydro-2*H*-pyran-2-yl)oxy)-16,16-bis((3-((3-(5-(((3*R*,4*R*,5*R*,6*R*)-3-acetamido-4,5-dihydroxy-6-(hydroxymethyl)tetrahydro-2*H*-pyran-2-yl)oxy)pentanamido)propyl)amino)-3-oxopropoxy)methyl)-5,11,18-trioxo-14-oxa-6,10,17-triazanonacosan-29-yl)-1*H*-1,2,3-triazol-4-yl)methyl)amino)-1-oxo-6-(2-(4,7,10-tris(carboxymethyl)-1,4,7,10-tetraazacyclododecan-1-yl)acetamido)hexan-2-yl)amino)-5-(15-carboxypentadecanamido)-6-oxohexyl)amino)-6-oxohexyl)-1,1-dimethyl-2-((1*E*,3*E*,5*Z*)-5-(1,1,3-trimethyl-1,3-dihydro-2*H*-benzo[*e*]indol-2-ylidene)penta-1,3-dien-1-yl)-1*H*-benzo[*e*]indol-3-ium]: Compound **19** (8.00 mg, 0.003 mmol) was dissolved in 1 M $NaHCO_3$ (0.5 mL, pH 8.5). Cy 5.5-NHS ester (3.50 mg, 0.0046 mmol) in DMF (0.5 mL) was added dropwise to reaction and mixture was stirred for 1 h at room temperature. The completion of reaction was confirmed by ESI-LRMS. The product was purified by RP-HPLC and lyophilized to give compound **20** as a light blue solid (1.2 mg, 13%).

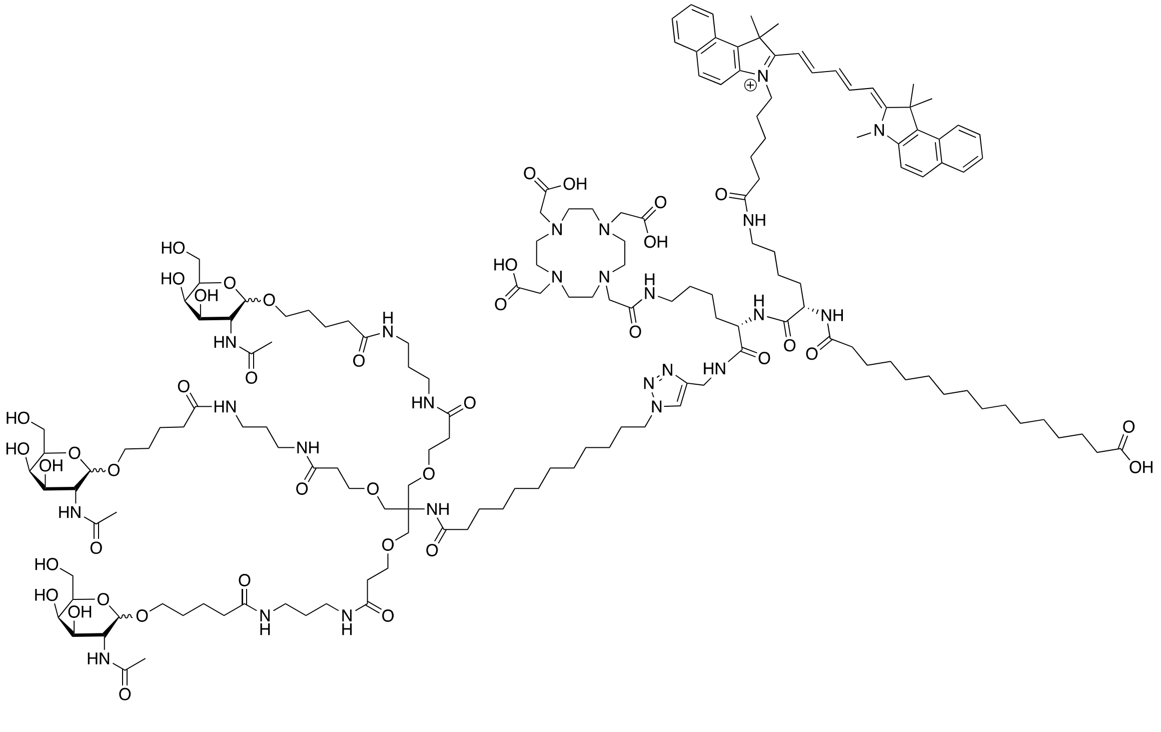

20

HRMS (ES^+) m/z $C_{160}H_{259}N_{24}O_{41}^+$ requires 1057.9574 $[M-2H]^{3+}$; found 1057.9565 $[M-2H]^{3+}$. RP-HPLC: 92% purity. $\lambda_{max}/\lambda_{em}$ (H_2O) 640/680 nm. Molar extinction coefficient = 194,000 M^{-1} cm^{-1}.

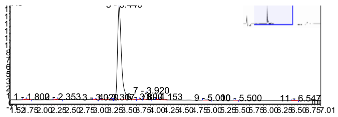

Compound 22 [1-benzyl 43-(*tert*-butyl) (2*R*,25*S*,*E*)-2-(4-((((9*H*-fluoren-9-yl)methoxy)carbonyl)amino)butyl)-25-(2-(*tert*-butoxy)-2-oxoethyl)-4,13,22,27-tetraoxo-6,9,15,18-tetraoxa-3,12,21,26-tetraazatritetracont-34-enedioate]: A solution of benzyl N^6-(((9*H*-fluoren-9-yl)methoxy)carbonyl)-*D*-lysinate (0.30 g, 0.66 mmol), (*S*,*E*)-22-(2-(*tert*-butoxy)-2-oxoethyl)-42,42-dimethyl-10,19,24,40-tetraoxo-3,6,12,15,41-pentaoxa-9,18,23-triazatritetracont-31-enoic acid (0.66 g, 0.78 mmol), DIPEA (0.23 mL, 1.31 mmol) and HATU (0.25 g, 0.66 mmol) in anhydrous DMF (5 mL) was stirred at room temp for overnight. The product formation was confirmed by ESI-LRMS. After completion of reaction, the product was purified by RP-HPLC and lyophilized to give compound 22 as a sticky yellowish solid (0.47 g, 56%).

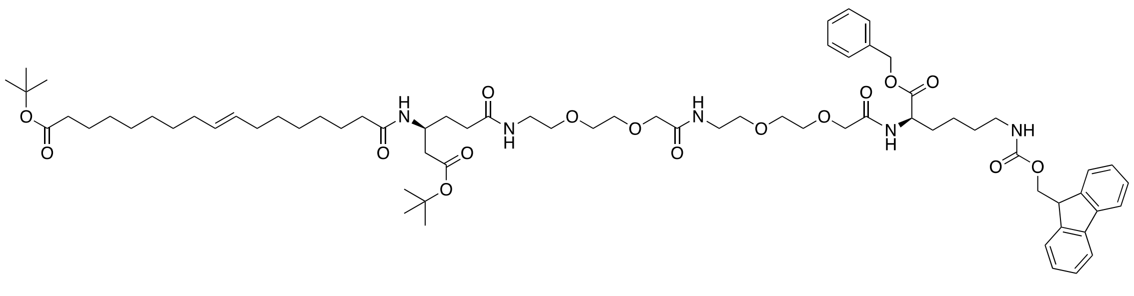

22

^{1}H NMR (300 MHz, CHLOROFORM-*d*) δ ppm: 1.25 – 1.37 (m, 18 H, 9 x -CH_2-), 1.44 (s, 9 H, 3x -CH_3), 1.45 (s, 9 H, 3x -CH_3), 1.52 – 1.77 (m, 6 H, 3x -CH_2-), 1.84 – 2.03 (m, 6 H, 3x -CH_2-), 2.16 – 2.27 (m, 4 H, 2x -CH_2-), 2.32 (t, *J*=7.2 Hz, 2 H, -NHCO-CH_2-), 3.13 (br. s., 2 H, 2x -N*H*-), 3.38 – 3.75 (m, 18 H, 9x -CH_2-), 3.96 – 4.08 (m, 4 H, -O-CH_2-CH_2-O), 4.18 – 4.30 (m, 3 H, -O-CH_2-CONH-, -C*H*-), 4.33 – 4.50 (m, 3 H, -C-CH_2-OCONH-, -C*H*-), 4.62 – 4.74 (m, 1 H, -C*H*-), 5.08 (br. s., 1 H, -N*H*-), 5.19 (s, 2 H, -COO-CH_2-C_6H_5), 5.29 – 5.43 (m, 2 H, -C*H*=C*H*-), 6.76 (br. s., 1 H, -N*H*-), 7.05 (br. s., 1 H, -N*H*-) 7.28 – 7.36 (m, 7 H, 7 x -CH_{Ar}), 7.40 (t, *J*=7.5 Hz, 2 H, 2 x -CH_{Ar}), 7.59 (d, *J*=7.2 Hz, 2 H, 2 x -CH_{Ar}), 7.76 (d, *J*=7.5 Hz, 2 H, 2 x -CH_{Ar}).

^{13}C NMR (75 MHz, CHLOROFORM-*d*) δ ppm: 22.41, 25.09, 25.63, 27.93, 28.11, 28.60, 28.97, 29.04, 29.16, 29.21, 29.55, 32.05, 32.35, 32.53, 35.62, 36.43, 38.66, 39.51, 40.61, 47.27, 51.49, 52.53, 66.47, 67.24, 70.59, 79.90, 82.50, 119.95, 124.99, 127.02, 127.67, 128.33, 128.64, 130.33, 135.20, 141.31, 143.96, 170.09, 170.67, 170.89, 171.95, 173.15, 173.37, 174.23.

HRMS (ES$^+$) m/z $C_{71}H_{105}N_5O_{16}$ requires 1284.7629 $[M+H]^+$; found 1284.7653 $[M+H]^+$.

Compound 23 [*tert*-butyl (10*S*,13*R*,36*S*)-13-(4-((((9*H*-fluoren-9-yl)methoxy)carbonyl)amino)butyl)-36-(2-(*tert*-butoxy)-2-oxoethyl)-2,2-dimethyl-4,12,15,24,33,38-hexaoxo-10-(prop-2-yn-1-ylcarbamoyl)-3,17,20,26,29-pentaoxa-5,11,14,23,32,37-hexaazatetrapentacontan-54-oate]: A solution of compound **22** (0.45 g, 0.35 mmol), (10%) Pd-C (1/10, w/w) in THF (5 mL) under H_2 (40 psi) was stirred at room temperature in a Parr apparatus for 6h. The reaction mixture was filtered through Celite, the filtrate evaporated under reduced pressure. An acid intermediate formation was confirmed by ESI-LRMS.

LRMS (ES$^+$) m/z $C_{64}H_{101}N_5O_{16}$ requires 1196.53 $[M+H]^+$; found 1196.88 $[M+H]^+$.

The crude acid intermediate (0.25 g, 0.21 mmol), compound **15** (71.0 mg, 3.21 mmol), DIPEA (0.073 mL, 0.42 mmol) and HATU (0.08 g, 0.21 mmol) in anhydrous DMF (5 mL) was stirred at room temp overnight. The completion of reaction was confirmed by ESI-LRMS. The final product was purified by RP-HPLC and lyophilized to give compound 23 as an off-white sticky solid (0.13 g, 43%).

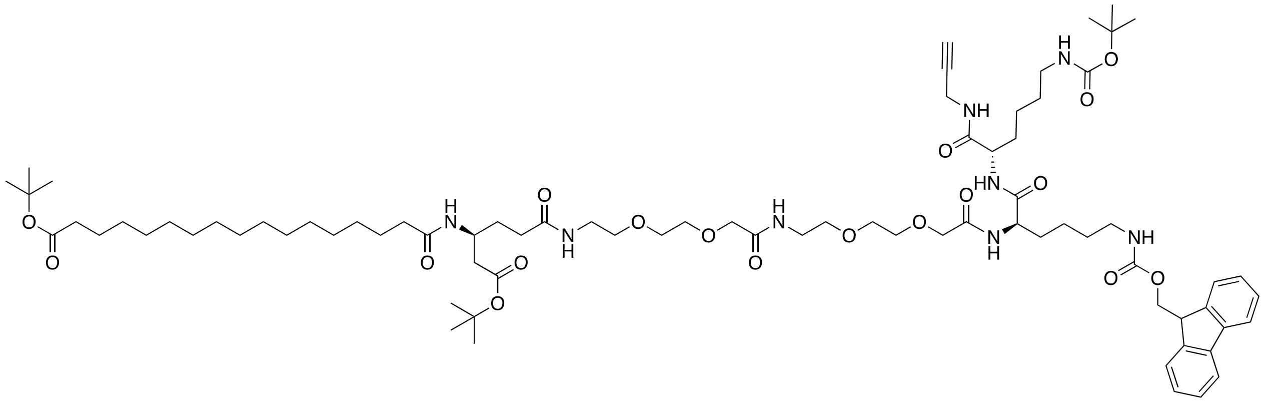

23

^{1}H NMR (300 MHz, CHLOROFORM-*d*) δ ppm: 1.22 – 1.34 (m, 26 H, 13 x -C*H$_2$*-), 1.43 (s, 9 H, 3x -C*H$_3$*), 1.44 (s, 9 H, 3x -C*H$_3$*), 1.46 (s, 9 H, 3x -C*H$_3$*), 1.50 – 1.98 (m, 12 H, 6x -C*H$_2$*-), 2.07 – 2.27 (m, 6 H, 3x -C*H$_2$*-), 1.95 (t, *J*=7.2 Hz, 2 H, -NHCO-C*H$_2$*-), 3.01 – 3.30 (m, 3 H, -C*H$_2$*-, -C*H*-), 3.39 – 3.74 (m, 16 H, 8x -C*H$_2$*-), 3.94 – 4.12 (m, 6 H, 3x -C*H$_2$*-), 4.18 – 4.27 (m, 1 H, -C*H*-), 4.33 – 4.48 (m, 5 H, 2x -C*H$_2$*-OCONH-, -C*H*-), 4.54 – 4.72 (m, 4 H, -C*H$_2$*-, 2x -C*H*-), 4.86 (br. s., 1 H, -N*H*-), 5.38 (br. s., 1 H, -N*H*-), 6.71 (d, *J*=7.5 Hz, 1 H, -N*H*-), 7.10 (d, *J*=7.74 Hz, 1 H, -N*H*-), 7.20 (m, 2 H, 2 x -N*H*-), 7.32 (t, *J*=7.5 Hz, 2 H, 2 x -C*H*$_{Ar}$), 7.40 (t, *J*=7.5 Hz, 2 H, 2 x -C*H*$_{Ar}$), 7.47 (m, 2 x -N*H*-), 7.60 (d, *J*=7.5 Hz, 2 H, 2 x -C*H*$_{Ar}$), 7.76 (d, *J*=7.5 Hz, 2 H, 2 x -C*H*$_{Ar}$).

^{13}C NMR (75 MHz, CHLOROFORM-*d*) δ ppm: 22.41, 22.61, 25.11, 25.69, 27.94, 28.10, 28.42, 29.08, 29.29, 29.47, 29.66, 31.44, 31.94, 32.55, 35.63, 36.53, 38.79, 39.41, 47.26, 52.60, 52.75, 53.12, 69.68, 69.95, 70.05, 70.46, 70.83, 71.06, 71.57, 79.31, 79.90, 82.56, 119.95, 125.03, 127.04, 127.66, 139.63, 141.28, 142.62, 143.97, 152.80, 156.67, 170.46, 170.77, 171.01, 171.35, 171.71, 173.18, 173.41, 174.19, 174.94.

HRMS (ES$^+$) m/z $C_{78}H_{124}N_8O_{18}$ requires 1461.9106 [M+H]$^+$; found 1462.9127 [M+H]$^+$.

Compound 24 [(6*S*,9*S*,32*S*)-9-(4-((((9*H*-fluoren-9-yl)methoxy)carbonyl)amino)butyl)-6-(4-aminobutyl)-32-(carboxymethyl)-5,8,11,20,29,34-hexaoxo-13,16,22,25-tetraoxa-4,7,10,19,28,33-hexaazapentacont-1-yn-50-oic acid]: A solution of compound **23** (0.13 g, 0.09 mmol) was dissolved in CH_2Cl_2 (4 mL), TFA (4 mL) was added and stirred at room temperature

for 3 h. After completion, the reaction mixture was evaporated and the residue was purified by RP-HPLC and lyophilized to give compound **24** as an off-white solid (76 mg, 68%).

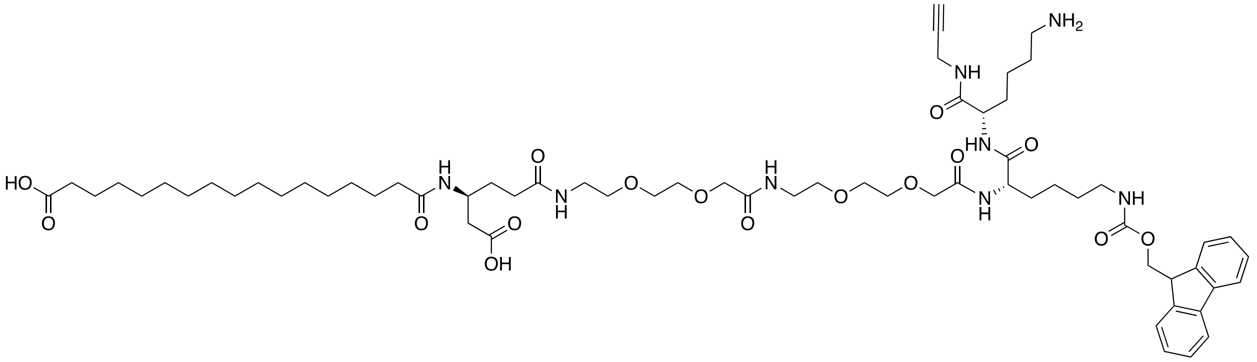

24

^{1}H NMR (300 MHz, CH_3CN+D_2O) δ ppm: 1.12 – 1.33 (m, 26 H, 13 x -C*H₂*-), 1.33 – 1.75 (m, 10 H, 5x -CH_2-), 2.13 (t, *J*=7.2 Hz, 2 H, -NHCO-CH_2-), 2.17 – 2.25 (m, 4 H, 2x -CH_2-), 2.94 – 3.37 (m, 19 H, 9x -CH_2-, -C*H*-), 3.42 – 3.74 (m, 11 H, 5x -CH_2-, -C*H*-**)**, 3.82 – 3.92 (m, 5 H, 2x -CH_2-OCONH-, -C*H*-), 4.22 – 4.34 (m, 4 H, -CH_2-, 2x -C*H*-), 7.29 (t, *J*=7.2 Hz, 2 H, 2 x -CH_{Ar}), 7.38 (t, *J*=7.2 Hz, 2 H, 2 x -CH_{Ar}), 7.59 (d, *J*=7.5 Hz, 2 H, 2 x -CH_{Ar}), 7.78 (d, *J*=7.5 Hz, 2 H, 2 x -CH_{Ar}).

^{13}C NMR (75 MHz, CHLOROFORM-*d*) δ ppm: 22.41, 22.61, 25.11, 25.69, 27.94, 28.10, 28.42, 29.08, 29.29, 29.47, 29.66, 31.44, 31.94, 32.55, 35.63, 36.53, 38.79, 39.41, 47.26, 52.60, 52.75, 53.12, 69.68, 69.95, 70.05, 70.46, 70.83, 71.06, 71.57, 79.31, 79.90, 82.56, 119.95, 125.03, 127.04, 127.66, 139.63, 141.28, 142.62, 143.97, 152.80, 156.67, 170.46, 170.77, 171.01, 171.35, 171.71, 173.18, 173.41, 174.19, 174.94.

HRMS (ES^+) m/z $C_{65}H_{100}N_8O_{16}$ requires 1249.7330 $[M+H]^+$; found 1249.7340 $[M+H]^+$.

Compound 25 [(9*S*,32*R*)-9-(((*S*)-1-(((1-(1-(((3*R*,4*R*,5*R*,6*R*)-3-acetamido-4,5-diacetoxy-6-(acetoxymethyl)tetrahydro-2*H*-pyran-2-yl)oxy)-16,16-bis((3-((3-(5-(((3*R*,4*R*,5*R*,6*R*)-3-acetamido-4,5-diacetoxy-6-(acetoxymethyl)tetrahydro-2*H*-pyran-2-yl)oxy)pentanamido)propyl)amino)-3-oxopropoxy)methyl)-5,11,18-trioxo-14-oxa-6,10,17-triazanonacosan-29-yl)-1*H*-1,2,3-triazol-4-yl)methyl)amino)-6-amino-1-oxohexan-2-yl)carbamoyl)-32-(carboxymethyl)-1-(9*H*-fluoren-9-yl)-3,11,20,29,34-pentaoxo-2,13,16,22,25-pentaoxa-4,10,19,28,33-pentaazapentacontan-50-oic acid]: A solution of

compound **6** (0.150 g, 0.074 mmol), compound **24** (92.0 mg, 0.074 mmol) and additive tris((1-benzyl-1H-1,2,3-triazol-5-yl)methyl)amine (39.0 mg, 0.074 mmol) were dissolved in THF:H_2O (9:1, 1 mL), stirred at room temperature and flushed by argon. Freshly prepared hydrated solution of sodium ascorbate (4 mg, 0.022 mmol) and $CuCl_2.2H_2O$ (4 mg, 0.022 mmol) were properly mixed in closed vial. This mixture was added to reaction mixture as one lot and stirring continued at room temperature for 1 h. The reaction progress was monitored ESI-LRMS. After completion of reaction, Cu was quenched by 1M EDTA (100 µL) and product was purified by RP-HPLC and lyophilized to give compound 25 as a white solid (0.15 g, 62%).

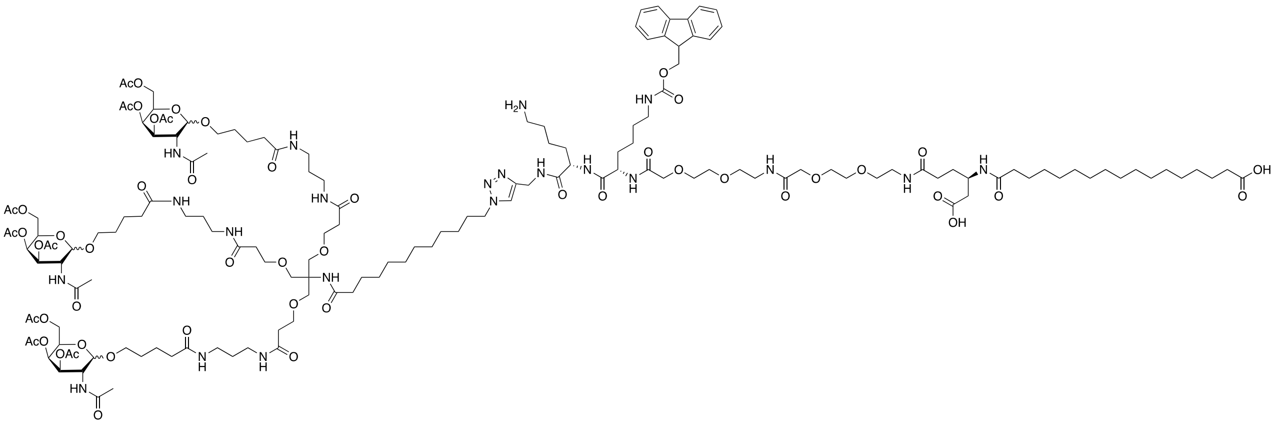

25

LRMS (ES^+) m/z $C_{156}H_{249}N_{21}O_{53}$ requires 3266.80 $[M+H]^+$; found 3267.18 $[M+H]^+$.

Compound 26 [2,2',2''-(10-((8*S*,11*S*,34*R*)-8-(((1-(1-(((3*R*,4*R*,5*R*,6*R*)-3-acetamido-4,5-dihydroxy-6-(hydroxymethyl)tetrahydro-2*H*-pyran-2-yl)oxy)-16,16-bis((3-((3-(5-(((3*R*,4*R*,5*R*,6*R*)-3-acetamido-4,5-dihydroxy-6-(hydroxymethyl)tetrahydro-2*H*-pyran-2-yl)oxy)pentanamido)propyl)amino)-3-oxopropoxy)methyl)-5,11,18-trioxo-14-oxa-6,10,17-triazanonacosan-29-yl)-1*H*-1,2,3-triazol-4-yl)methyl)carbamoyl)-11-(4-aminobutyl)-51-carboxy-34-(carboxymethyl)-2,10,13,22,31,36-hexaoxo-15,18,24,27-tetraoxa-3,9,12,21,30,35-hexaazahenpentacontyl)-1,4,7,10-tetraazacyclododecane-1,4,7-triyl)triacetic acid]: Compound **24** (0.10 g, 0.03 mmol) was dissolved in DMF (0.5 mL), added to 1M $NaHCO_3$ solution (2 mL) and stirred at room temperature. DOTA-NHS ester (35.0 mg, 0.046 mmol) was dissolved in DMF (0.5 mL), added dropwise to reaction mixture and stirred for 1 h at room temperature. The completion of reaction was confirmed by ESI-LRMS.

LRMS (ES$^+$) m/z $C_{157}H_{265}N_{25}O_{58}$ requires 3430.96 [M+H]$^+$; found 3431.13 [M+H]$^+$.

The crude solution was lyophilized, dissolved in MeOH (2 mL) and NaOAc (20.0 mg, 0.37 mmol) was added to the reaction mixture. The completion of deacylation at GalNAc was confirmed by ESI-LRMS. Finally, solvent was evaporated under reduced pressure and the residue was dissolved in DMF (2 mL), diethylamine (1 mL) was added and stirred at room temperature for 1 h. The completion of reaction was confirmed by ESI-LRMS and the final product was purified by RP-HPLC and lyophilized to give compound **26** as a white solid (20 mg, 22%).

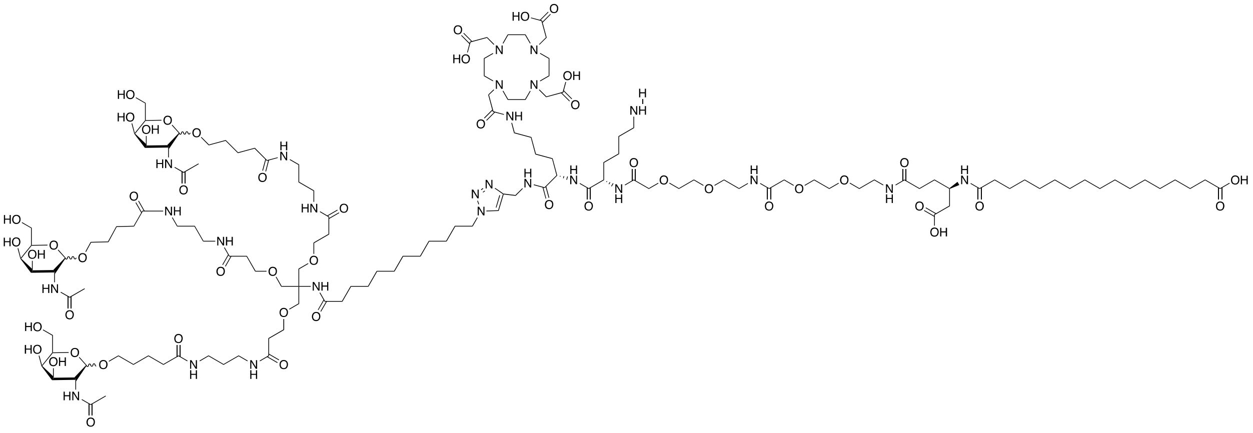

26

^{1}H NMR (700 MHz, DMSO-*d*$_6$) δ ppm: 1.17 (br s, 1 H) 1.23 (br s, 1 H) 1.26 (s, 2 H) 1.45 (br s, 1 H) 1.45 (br d, *J*=6.68 Hz, 1 H) 1.44 - 1.46 (m, 1 H) 1.49 (br s, 1 H) 1.50 (br s, 1 H) 1.50 - 1.53 (m, 1 H) 1.50 - 1.54 (m, 1 H) 1.53 (br s, 1 H) 1.70 (br s, 1 H) 1.77 - 1.81 (m, 1 H) 1.77 - 1.78 (m, 1 H) 1.79 - 1.80 (m, 1 H) 1.83 (d, *J*=3.29 Hz, 1 H) 1.89 (s, 1 H) 1.94 - 1.95 (m, 1 H) 2.05 - 2.10 (m, 1 H) 2.07 (br t, *J*=7.72 Hz, 1 H) 2.12 (br t, *J*=7.80 Hz, 1 H) 2.13 - 2.17 (m, 1 H) 2.20 (s, 1 H) 2.30 (br t, *J*=6.63 Hz, 1 H) 3.03 - 3.06 (m, 1 H) 3.07 (br s, 1 H) 3.22 (br d, *J*=5.98 Hz, 1 H) 3.30 (br s, 1 H) 3.30 - 3.33 (m, 1 H) 3.30 - 3.34 (m, 1 H) 3.37 (br d, *J*=9.80 Hz, 1 H) 3.37 (br s, 1 H) 3.41 - 3.48 (m, 1 H) 3.42 - 3.44 (m, 1 H) 3.43 - 3.46 (m, 1 H) 3.44 - 3.45 (m, 1 H) 3.45 - 3.46 (m, 1 H) 3.49 - 3.60 (m, 1 H) 3.53 - 3.55 (m, 1 H) 3.55 - 3.57 (m, 1 H) 3.55 - 3.57 (m, 1 H) 3.55 - 3.58 (m, 1 H) 3.58 - 3.60 (m, 1 H) 3.60 - 3.60 (m, 1 H) 3.67 - 3.67 (m, 1 H) 3.72 (br s, 1 H) 3.70 - 3.71 (m, 1 H) 3.70 - 3.74 (m, 1 H) 3.71 - 3.80 (m, 1 H) 3.73 - 3.75 (m, 1 H) 3.87 (s, 1 H) 3.90 (s, 1 H) 3.96 (br s, 1 H) 3.99 - 3.99 (m, 1 H) 4.06 - 4.06 (m, 1 H) 4.13 - 4.14 (m, 1 H) 4.15 - 4.15 (m, 1 H) 4.23 - 4.27 (m, 1 H) 4.24 (br d, *J*=8.76 Hz, 1 H) 4.31 (br d, *J*=7.37 Hz, 1 H) 4.31 - 4.32 (m,

1 H) 4.35 - 4.38 (m, 1 H) 5.36 - 5.51 (m, 1 H) 6.41 - 6.74 (m, 1 H) 7.01 (s, 1 H) 7.42 - 7.56 (m, 1 H) 7.66 (br s, 1 H) 7.68 - 7.69 (m, 1 H) 7.70 - 7.70 (m, 1 H) 7.75 - 7.79 (m, 1 H) 7.77 - 7.77 (m, 1 H) 7.85 - 7.88 (m, 1 H) 7.90 (s, 1 H) 7.95 - 7.95 (m, 1 H) 8.03 - 8.03 (m, 1 H) 8.10 - 8.12 (m, 1 H).

^{13}C NMR (176 MHz, DMSO-*d*$_6$) δ ppm: 22.32, 22.43, 23.44, 23.49, 23.50, 24.97, 25.73, 25.79, 26.40, 28.96, 29.16 (br dd, *J*=34.01, 17.48 Hz, 1 C), 29.06, 29.13, 29.37, 29.42, 29.44, 29.46, 29.50, 29.53, 29.80, 30.29, 32.24, 34.15, 35.55, 35.62, 36.39, 36.45, 36.49, 36.62, 36.75, 36.84, 38.44, 38.85, 38.97, 39.32, 39.37, 39.68, 40.49, 40.62, 40.74, 49.75, 51.77, 52.54, 59.96, 60.93, 65.08, 67.80, 67.97, 68.27, 68.36, 68.67, 68.73, 69.38, 69.56, 69.79, 70.39, 70.59, 70.65, 71.23, 71.99, 75.72, 101.55, 101.85, 115.29, 117.00, 118.71, 122.99, 145.10, 158.14, 158.32, 169.81, 170.04, 170.59, 172.10, 172.50, 172.55, 172.71, 173.01, 174.98.

HRMS (ES$^+$) m/z $C_{139}H_{245}N_{25}Na_2O_{49}$ requires 1033.9296 [M-2H]$^{3+}$; found 1033.9241 [M-2H]$^{3+}$.

Compound 27 [3-((35*S*)-12-(((*S*)-1-(((1-(1-(((3*R*,4*R*,5*R*,6*R*)-3-acetamido-4,5-dihydroxy-6-(hydroxymethyl)tetrahydro-2*H*-pyran-2-yl)oxy)-16,16-bis((3-((3-(5-(((3*R*,4*R*,5*R*,6*R*)-3-acetamido-4,5-dihydroxy-6-(hydroxymethyl)tetrahydro-2*H*-pyran-2-yl)oxy)pentanamido)propyl)amino)-3-oxopropoxy)methyl)-5,11,18-trioxo-14-oxa-6,10,17-triazanonacosan-29-yl)-1*H*-1,2,3-triazol-4-yl)methyl)amino)-1-oxo-6-(2-(4,7,10-tris(carboxymethyl)-1,4,7,10-tetraazacyclododecan-1-yl)acetamido)hexan-2-yl)carbamoyl)-52-carboxy-35-(carboxymethyl)-6,14,23,32,37-pentaoxo-16,19,25,28-tetraoxa-7,13,22,31,36-pentaazadopentacontyl)-1,1-dimethyl-2-((1*E*,3*E*,5*Z*)-5-(1,1,3-trimethyl-1,3-dihydro-2*H*-benzo[*e*]indol-2-ylidene)penta-1,3-dien-1-yl)-1*H*-benzo[*e*]indol-3-ium]: Compound **26** (10 mg, 3.0 μmol) was dissolved in 1 M $NaHCO_3$ (0.5 mL, pH 8.5). Cy 5.5-NHS ester (4.0 mg, 5.0 μmol) in DMF (0.5 mL) was added dropwise to reaction and mixture was stirred for 1 h at room temperature. The completion of reaction was confirmed by ESI-LRMS. The product was purified by RP-HPLC and lyophilized to give compound 27 as a light blue solid (2 mg, 18%).

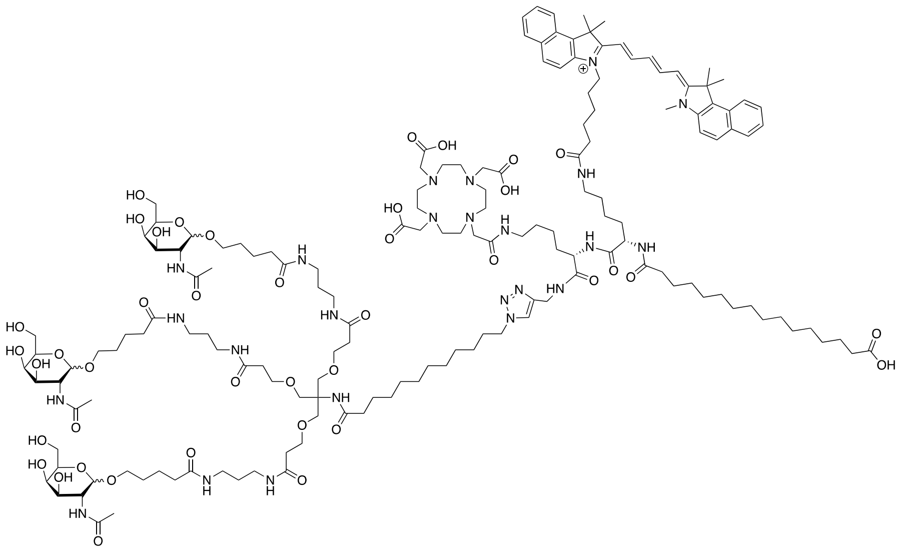

27

HRMS (ES^+) m/z $C_{179}H_{288}N_{27}Na_3O_{50}^+$ requires 1228.6843 $[M-2H]^{3+}$; found 1228.6662 $[M-2H]^{3+}$. RP-HPLC: 92% purity. $\lambda_{max}/\lambda_{em}$ (H_2O) 640/680 nm. Molar extinction coefficient = 189,000 M^{-1} cm^{-1}.

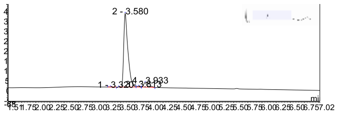

Compound 28: Compound **6** (0.2 g, 0.1 mmol) was dissolved in MeOH (2 mL) and NaOMe (64 mg, 1.2 mmol) was added to the reaction mixture. The completion of deacylation at GalNAc was confirmed by ESI-LRMS. The final product was purified by RP-HPLC and lyophilized to give compound **27** as a white solid (0.11 g, 72%).

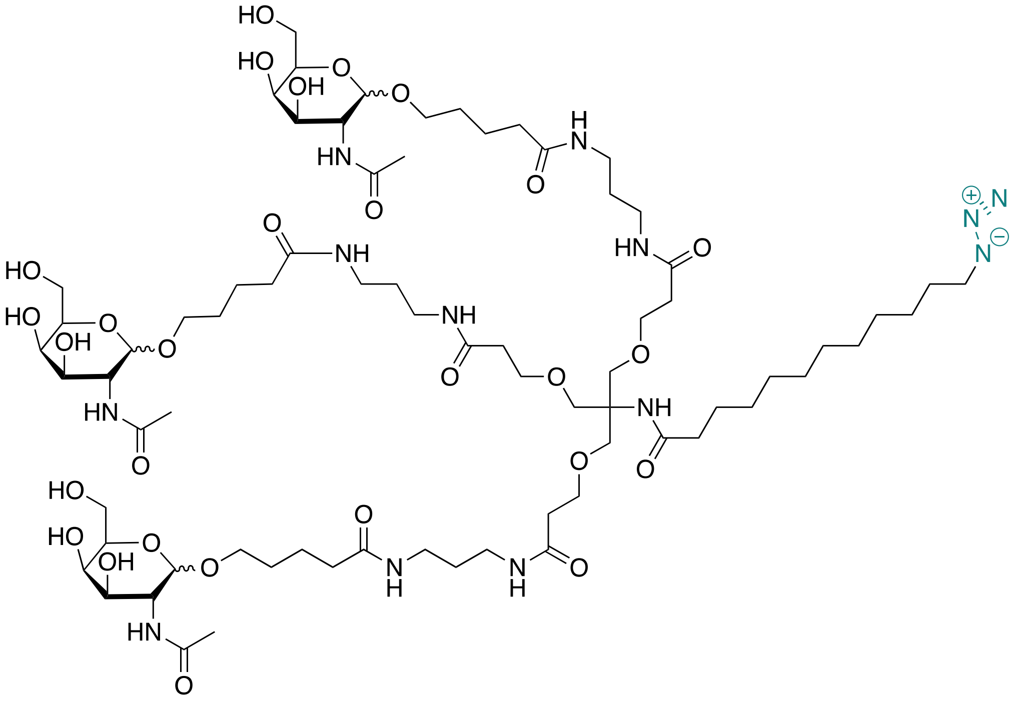

28

Atom#	C Shift [ppm]	XHn	H Shift [ppm]	C/X Multiplicity	H Multiplicity
55, 74, 93	22,026	CH2	1,694	s	m
71, 90, 110	22,229	CH3	2,092	s	m
22	25,287	CH2	1,620	s	m
30	25,981	CH2	1,437	s	m
31	28,007	CH2	1,652	s	br s
56, 75, 94	28,171	CH2	1,640	s	m
5, 38, 49	28,305	CH2	1,781	m	quin (1.00, 1.00, 1.00, 1.00)
23, 24, 25, 26, 27, 29	28,575	CH2	1,355	s	br s
54, 73, 92	35,429	CH2	2,325	s	m
21	36,394	CH2	2,274	s	m
9, 33, 44	36,394	CH2	2,567	s	t (6.11, 6.11)
4, 39, 50	36,730	CH2	3,286	s	m
111	51,234	CH2	3,391	s	m
64, 83, 102	52,463	CH	3,955	s	m
65, 84, 103	60,943	CH2	3,854	s	m
10, 32, 43	67,472	CH2	3,792	s	m
62, 81, 100	67,822	CH	4,016	s	d (3.55)
13, 15, 17, 14	68,583	C, CH2	3,742	s	m
57, 76, 95	69,778	CH2	3,672	s	m
57, 76, 95	69,778	CH2	3,997	s	br d (10.66)
63, 82, 101	70,887	CH	3,804	u	m
61, 80, 99	75,088	CH	3,742	s	m
59, 78, 97	101,611	CH	4,517	s	d (8.67)
8, 34, 45	174,002	C		s	
70, 89, 108	174,564	C		s	
1, 41, 52	176,416	C		s	
20	176,938	C		s	
6, 37, 48	36.85	CH2	3,304	s	t (1.00, 1.00)

HRMS (ES^+) m/z $C_{73}H_{131}N_{13}O_{28}$ requires 1638.92$[M+H]^+$; found 1638.9262 $[M+H]^+$.

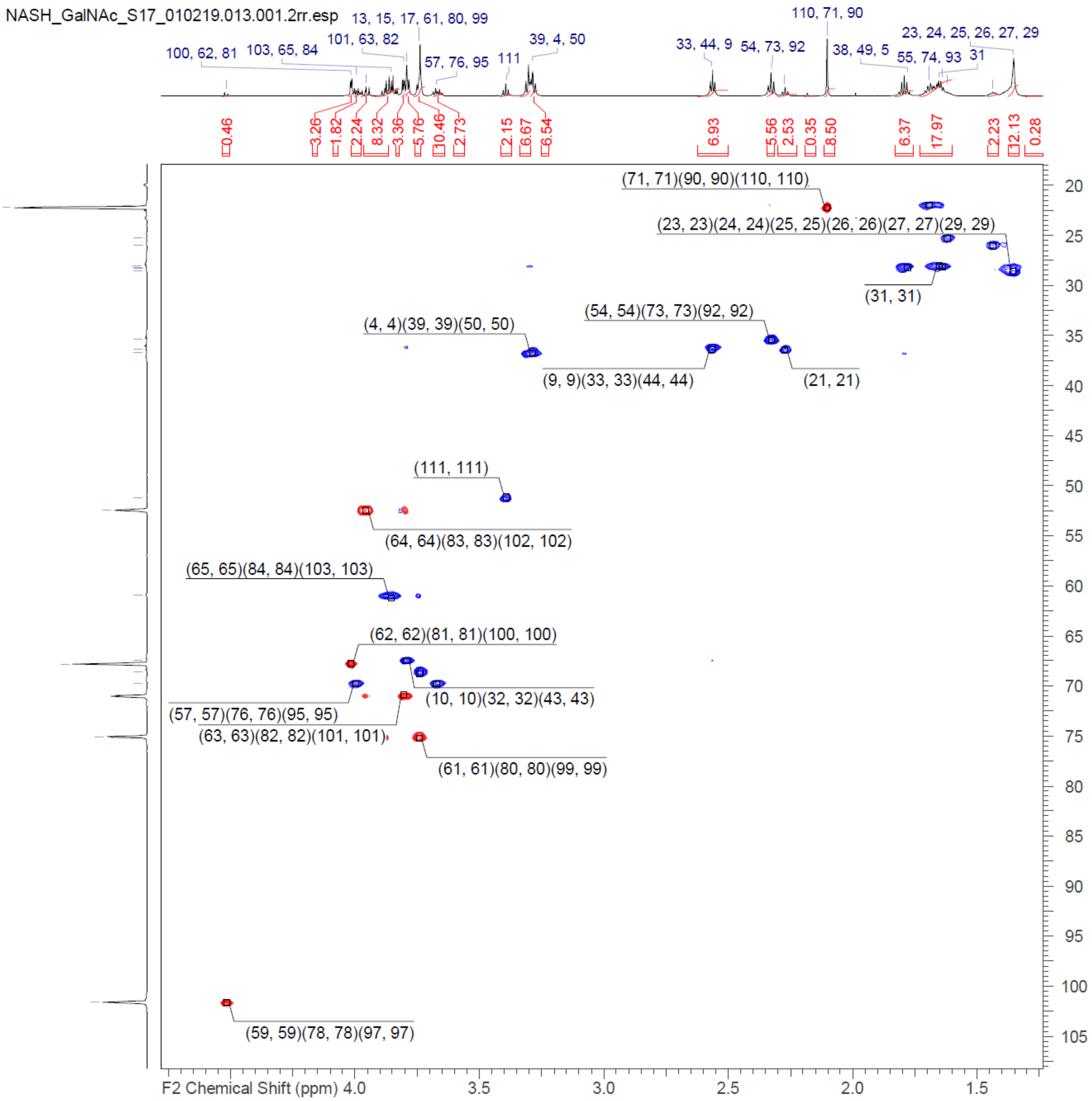
NASH_GalNAc_S17_010219.013.001.2rr.esp
100, 62, 81
103, 65, 84
101, 63, 82
13, 15, 17, 61, 80, 99
57, 76, 95
111
39, 4, 50
33, 44, 9
54, 73, 92
110, 71, 90
38, 49, 5
55, 74, 93
31
23, 24, 25, 26, 27, 29
0.46
3.26
1.82
2.24
8.32
3.36
5.76
10.46
2.73
2.15
6.67
6.54
6.93
5.56
2.53
0.35
8.50
6.37
17.97
2.23
12.13
0.28
(71, 71)(90, 90)(110, 110)
(23, 23)(24, 24)(25, 25)(26, 26)(27, 27)(29, 29)
(31, 31)
(54, 54)(73, 73)(92, 92)
(4, 4)(39, 39)(50, 50)
(9, 9)(33, 33)(44, 44)
(21, 21)
(111, 111)
(64, 64)(83, 83)(102, 102)
(65, 65)(84, 84)(103, 103)
(62, 62)(81, 81)(100, 100)
(10, 10)(32, 32)(43, 43)
(57, 57)(76, 76)(95, 95)
(63, 63)(82, 82)(101, 101)
(61, 61)(80, 80)(99, 99)
(59, 59)(78, 78)(97, 97)
F2 Chemical Shift (ppm) 4.0
3.5
3.0
2.5
2.0
1.5
20
25
30
35
40
45
50
55
60
65
70
75
80
85
90
95
100
105

9. General radiochemistry experimental details.

Materials.

The PSH^+ ion-exchange cartridge was purchased from ABX GmbH (Radeberg, Germany), the 0.05 M HCl (Alfa Aesar 035646.K2) from Fisher Scientific, and Oasis Light HLB SPE cartridges from Waters GmbH. Buffer was prepared from HEPES powder (Fisher Bioreagents) and stored at 4 °C. Ultrapure Ampuwa water (Fresenius Kabi Deutschland GmbH) was used unless otherwise stated.

Radiolabelling.

^{68}Ga was obtained from a commercially available itG 68-Ge/68-Ga generator (1100 MBq or 2000 MBq), and the labelling was performed using a commercial automated Scintomics GRP system. A general protocol consisted of ^{68}Ga elution from the generator with 0.05 M HCl (9 mL), with the eluate passing through a PSH^+ cation exchanged cartridge. The cartridge was then washed with water and ^{68}Ga was recovered using 1.5 mL of the eluent consisting of 5 M aq. NaCl and 37 µL 5.5 M aq. HCl directly into the reaction vessel containing GalNAc.NASH probes (20 µg) in 2.4 mL HEPES buffer (1.5 M, pH 4.5). The reaction mixture was heated at 100 °C for 20 min and thereafter the crude product was transferred onto a C18 solid phase extraction cartridge. The cartridge was washed with water (10 mL) and the product was eluted using EtOH:H_2O (0.5:0.5, v/v, 1 mL) into a sterile product vial, and formulated with PBS (3 mL, pH 7.4).

Radiochemical purity (HPLC-methods for quality control of gallium-68 labelled probes)

Radiochemical purity was determined using an Agilent 1100 series HPLC system equipped with a Xselect Peptide CSH 130 C18 50 x 4.6 mm 2.5 µm XP column (at 35 °C), an Agilent 1100 series diode array, and fLumo LB500 radioactivity detector.

<u>Chromatographic condition A:</u>

mob.Ph A:	water/acetonitrile / TFA	900 : 100 : 1
mob.Ph B:	water/acetonitrile / TFA	100 : 900 : 0.75
flow:	1 mL/min	
wavelength:	214 nm	

Inj-vol.: RD: 2µL typically

UV: 50µL (e.g.)

Gradient correlating to compound:

GN-01 min/%B: 0/05; 1/05; 6/15; 7.3/85; 7.9/85; 8/05; 11/05

GN-03 min/%B: 0/35; 1/35; 6/55; 7.3/90; 7.9/90; 8/35; 11/35

GN-05 min/%B: 0/25; 1/25; 6/50; 7.3/90; 7.9/90; 8/25; 11/25

Chromatographic condition B:

mob.Ph A: water/acetonitrile / TFA 900 : 100 : 1

mob.Ph B: water/acetonitrile / TFA 100 : 900 : 0.75

flow: 1 mL/min

wavelength: 214 nm

Inj-vol.: RD: 2µL typically

UV: 50µL (e.g.)

Gradient correlating to compound:

GN-02 min/%B: 0/10; 1/10; 6/20; 7.3/85; 7.9/85; 8/05; 11/05

GN-04 min/%B: 0/40; 1/40; 6/60; 7.3/90; 7.9/90; 8/35; 11/35

GN-06 min/%B: 0/30; 1/30; 6/55; 7.3/90; 7.9/90; 8/25; 11/25

Formulation stability.

Stability of the formulated product at room temperature was determined by radio-HPLC analysis at 0, 1 h, 2 h, 3 h and 4 h after synthesis, and was expressed as the radiochemical purity. Retention of radioactivity on the HPLC column was assessed by comparing the total radioactivity eluted to that eluted when the HPLC column was replaced with a restriction capillary.

Plasma and HSA stability.

A sample of either SD rat plasma (600 µL), or HSA solution (40 g/L) in DPBS buffer (600 µL), were incubated at 37 °C for 30 mins before addition of ^{68}Ga-GN probes formulation (200 µL, 30 ± 7 MBq), after which they were incubated at 37 °C. Samples were taken immediately, and after 30 min, 60 min. Each sample (100 µL) was treated with MeCN (100 µL) and the suspension

centrifuged for 5 mins at 13000 RPM and the clear supernatant analysed by radio-HPLC (as described above) to determine the percentage of intact product.

10. Physicochemical characterization by NMR

^{13}C oleic acid competition assay for GN-probes-HSA binding.

Lyophilized human serum albumin (HSA) purified from fatty acid free serum was purchased from Sigma Aldrich and dissolved in 50 m KH_2PO_4, 50 mM NaCl, 10 % D_2O buffer at pH 7.4. The concentration of HSA was determined by NanoDrop Photometer and set to 0.5 mM. Methyl-^{13}C oleic acid was purchased from Cambridge Isotopes and stock solutions prepared as described in Simard et al.[18] ^{1}H,^{13}C-HSQC spectra of samples containing 0.5 mM HSA and 2 mM methyl-^{13}C oleic acid were measured with a Bruker Avance Neo 700 MHz spectrometer with cryo probe at 298 K. Assignment of the peaks and competition studies were conducted according to the method described in [4]. GN probes (GN-01, GN-03, GN-05) were added in three titration steps of 0.25 mM, 0.5 mM and 1 mM concentration to the HSA/^{13}C oleic acid samples and the ^{1}H,^{13}C-HSQC spectra were recorded. Overlay of these spectra revealed the binding site of the probes to HSA in competition to the bound ^{13}C-oleic acid molecules.

Saturation Transfer Difference Spectroscopy (STD) measurements with ASGPR.

For ASGPR protein binding experiments, ^{1}H STD spectra[2] were recorded from compounds in buffer (as a negative control) and in presence of protein ASGPR. The buffer conditions were 25 mM d_{16}-Tris, 25 mM $CaCl_2$, 0.1 mM trimethylsilyl-3-propionic acid D_4-2,2,3,3, pH 7.0 in D_2O. The NMR samples contained 200 µM GN probes in buffer and with 10 µM ASGPR protein. STD spectra were recorded with the standard BRUKER pulse program stddiffesgp at 293 K and the irradiation frequency was set to 0.4 ppm to saturate the methyl protons of the protein. Binding of compounds with similar structure was quantified by the STD scaling factor which is the ratio of the intensities of signals in the STD spectrum to the signal intensities of a ^{1}H 1D spectrum recorded with the pulse sequence zgesgp.

11. X-ray diffraction data collection and structure determination

a) **<u>ASGPR – CPDY complex.</u>**

Crystallization.

The H1 subunit of human ASGPR, residues 148-291 from UniProt Entry P07306 with a C-terminal His6-tag was produced at Crelux, Martinsried, Germany, according to the publication of Meier et al.[1] ASGPR crystals of the GalNAc.NASH probes were grown by the hanging drop vapor diffusion method using a protein solution of 9 mg/ml ASGPR H1 domain, 10 mM GN probe, 20 mM Tris-HCl, pH 7.5, 150 mM NaCl and 25 mM $CaCl_2$ and a reservoir solution of 100 mM Tris-HCl, pH 7.4 and 28% (w/v) PEG4000. Crystals grew in about 1 month at 20 °C to full size. Streak-seeding was used to obtain bigger crystals.

To obtain crystals of the compound 28 (GN-A), a crystal was transferred to 1 µl reservoir solution, containing 10 mM compound 28 (GN-A). After overnight soaking, the crystal was picked up in a nylon loop, wetted with glycerol, and immediately flash frozen in liquid nitrogen. Previous experiments (not shown) had showed us that this procedure effectively removes the GalNAc used for the crystallization, and that any ligand observed in the electron density maps is the ligand of interest that was soaked in and not the ligand used for the crystallization.

Data collection and processing.

X-ray diffraction data were collected at beamline PX-II of the Swiss Light Source (SLS) in Villigen, Switzerland, and processed with XDS [19] and scaled with Aimless [20] and STARANISO [21] as implemented in autoProc[22]. The crystal was of space group C2 and contained one ASGPR-H1 molecule in the asymmetric unit. The unit cell dimensions of this crystal are as follows: a = 114.8, b= 32.5 and c = 39.7 Å with β = 93.8°. The crystal diffracted to 1.39 Å and R_{merge} was 8.0%. The diffraction was strongly anisotropic with the resolution in the best direction being 1.39 Å and 2.20 Å in the worst direction. Final data collection and refinement statistics are given in Table S1.

Structure solution and refinement.

The structure was determined by molecular replacement using the pdb structure 1dv8 [18] as a model. Since the cell parameters of our crystal were virtually identical to the cell parameters of 1dv8, no molecular replacement program was run. A rigid body refinement was sufficient to get the correct orientation of the model. Model building was done with Coot [23] and refinement was done with Buster [24] using default settings. The electron density of the N- and C-termini (residues

148-151 and 281-291 plus His6-tag) was very weak and could not be modeled. Otherwise, the electron density maps are of very high quality. The final model contains residues 152-280. The final data collection and refinement statistics are given in Table S1.

Table S1. Crystallographic Data Collection and Refinement Statistics for ASGPR (PDB code: 6YAU)

Data collection

Spacegroup name	*C2*
Unit cell parameters	114.75 32.53 39.65 90.00 93.82 90.00
Wavelength	1.00000 A

Diffraction limits & principal axes of ellipsoid fitted to diffraction cut-off surface:

1.386		0.8111	0.0000	-0.5849	0.965 _a_* - 0.262 _c_*
1.444	-	0.0000	1.0000	-0.0000	_b_*
2.205		0.5849	0.0000	0.8111	0.910 _a_* + 0.414 _c_*

	Overall	InnerShell	OuterShell
Low resolution limit	57.248	57.248	1.558
High resolution limit	1.397	4.715	1.397
R_{merge} (all I+ & I-)	0.080	0.036	0.932
R_{merge} (within I+/I-)	0.078	0.035	0.912
R_{meas} (all I+ & I-)	0.088	0.040	1.039
R_{meas} (within I+/I-)	0.091	0.041	1.031
R_{pim} (all I+ & I-)	0.034	0.016	0.449
R_{pim} (within I+/I-)	0.046	0.022	0.467
Total number of observations	103189	4940	4187
Total number unique	16299	815	815
Mean(I)/sd(I)	11.1	31.5	1.4
Completeness (spherical)	55.7	99.3	10.1
Completeness (ellipsoidal)	89.9	99.3	46.0
Multiplicity	6.3	6.1	5.1
CC(1/2)	0.998	0.999	0.593

Refinement

Protein atoms:	1115
Inhibitor atoms:	22
Water atoms:	174
Other atoms:	3
Resolution (Å)	57.25-1.39 (1.51-1.39)*
Rwork (%)	17.2 (19.9)
Rfree (%)	21.9 (21.0)
Average Bfactors (Å^2)	
Protein:	20.76
Inhibitor:	21.88
Water:	35.34
Other:	15.60
RMSD bond lengths (Å)	0.010
RMSD bond angles (°)	1.01

* the highest resolution bin is given in brackets

b) **<u>HSA – CPDX complex</u>**

Crystallization.

Essentially defatted human serum albumin from Sigma was purified by size exclusion chromatography to obtain pure monomeric protein [25]. The purified HSA was dissolved in 20 mM potassium phosphate (pH 7.0) and concentrated to 120 mg/mL. To the HSA solution we added 10 mM compound 24 and 3 mM myristate, dissolved DMSO. The final concentration of DMSO was 5% (v/v). The crystal was grown by the hanging drop vapor diffusion method using a reservoir solution containing 50 mM sodium-potassium-phosphate (pH 7.0), and 30% polyethylene glycol 3350. For crystallization 1 µL of HSA/compound 24 (GN-07) solution was equilibrated against 1 µL of reservoir solution. After about one-week, colorless crystals appeared.

Data collection and processing.

For cryoprotection, the nylon loop was wetted with glycerol and the crystal was picked directly from the crystallization drop and immediately flash frozen in liquid nitrogen.

X-ray diffraction data were collected at beamline PX-III of the Swiss Light Source (SLS) in Villigen, Switzerland, and processed with XDS [19] and scaled with Aimless [20] as implemented in autoProc [22]. The crystal was of space group C2 and contained one HSA molecule in the asymmetric unit. The unit cell dimensions of this crystal are as follows: a = 197.7, b= 38.41 and c = 95.4 Å with β = 105.9°. The crystal diffracted to 1.89 Å and R_{merge} was 6.3%. Final data collection and refinement statistics are given in Table S2.

Structure solution and refinement.

The structure was determined by molecular replacement using the program Phaser [26], using the pdb structure 2i30 [27] as search model. Model building was done with Coot [23] and refinement was done with Buster [24] using default settings. The electron density for the loops 363-365 and 553-564 was very weak and could not be modeled. Otherwise, the HSA molecule fits the electron density map quite well. The final model contains residues 1-362, 366-552 and 565-584. The final data collection and refinement statistics are given in Table S2.

We observed difference density for five bound myristate molecules in fatty acid sites 3, 4, 5, 6 and 7, and one very long blob of difference density in fatty acid sites 2 and 8, where we could fit

compound 24 (see figure 2B). The nomenclature for the fatty acid site was taken from Bhattacharya, A.A. et al [3]. The fatty acid part of compound 24 is very well defined but starting at the point where the linker is attached and the molecule leaves the binding pocket, the electron density becomes weak and fragmented, indicated that the linker is disordered in the solvent. After fitting 5 myristate molecules and one compound 24 molecule followed by more refinement, a final structure was obtained with a Rfactor of 23.9% and a free Rfactor of 31.3%. The free Rfactor is high, which might be due to some disorder in our crystals due to partial occupancy of some the myristate sites.

Table S2. Crystallographic Data Collection and Refinement Statistics for HSA (PDB code: 6YG9)

Data collection	
Space group	*C2*
Cell dimensions	
a,b,c (Å)	197.72 38.14 95.37 197.72 38.14 95.37
α,β,γ (°)	90.00 105.88 90.00 90.00 105.88 90.00
Resolution (Å)	91.73 -1.89 (1.99-1.89)*
<I>/sig<I>	11.7 (2.3)
Observed reflections	156458 (20574)
R_{meas} (%)	0.077 (0.620)
Completeness (%)	96.6 (95.7)
Redundancy	2.9 (2.7)
Refinement	
Protein atoms:	4497
Inhibitor atoms:	144
Water atoms:	662
Resolution (Å)	58.51-1.89 (1.94-1.89)*
R_{work} (%)	23.9 (24.5)
R_{free} (%)	31.3 (32.5)
Average Bfactors (Å^2)	
Protein:	35.94
Inhibitor:	42.41
Water:	39.71
rmsd bond lengths (Å)	0.010
rmsd bond angles (°)	1.20

* the highest resolution bin is given in brackets

12. *In cellulo* characterization of GN probes

DMEM culture medium, 5% FCS containing DMEM culture medium and Hoechst33342 were purchased from Invitrogen. HepG2 cells (human hepatocyte cell line) were cultured as according to the method reference manual (ATCC HB-8065, USA). Internalization and blocking experiments were performed on collagen I (Gibco) coated 96 well plates (Cell carrier, Perkin Elmer). Prior to the experiments the HepG2 cells were serum starved for 2h. Imaging was performed on a confocal microscope (Opera Phenix, Perkin Elmer) and the image analysis was performed with Harmony (Perkin Elmer).

To investigate the cell viability during compound treatment the nuclei number and shape was monitored over time using the harmony analysis software (Figure S3). The data shows no effect of the Cy5.5 labeled probes on HepG2 cells in the assay window and concentration of 1 µM.

13. Florescence polarization assays.

To determine the IC_{50} values of GN-probes, ASGPR-GN probes binding fluorescence polarization assays were performed on freshly prepared liver rat tissues. The method was opted from Moerke et al[28].

14. Animal studies for in vivo biodistribution studies

All experiments were approved by the Ethics Committee of the State Ministry of Agriculture, Nutrition and Forestry (State of Hessen, Germany).

For the proof of concept of the probe in PET analysis, female Sprague-Dawley rats (Charles River, Sulzfeld, Germany) (n = 19, 3-5 imaging experiments on single animal), weighing 400±89 g, were group-housed in a specific pathogen-free barrier facility on a 12:12 h light/dark cycle with *ad libitum* access to tap water and regular chow. The animals were not fasted before imaging.

CD-1 mice used in optical imaging studies were fed with chorofyll free diet (n= 12), EF Control with 5% fat low fluorescence, chlorophyll, Experimental diet, ssniff Spezialdiäten GmbH).

Whole body *in vivo* fluorescence images were acquired on an IVIS Spectrum Imaging System (Perkin Elmer) using the pre-defined filter set for Cy5.5 to correct for tissue autofluorescence.

Image analysis and quantification was performed using Living Image Software package (PerkinElmer). In brief, liver fluorescence intensity was determined using ROI measurements and subtraction of baseline fluorescence from the same body area of a control mouse.

15. Animals for NASH development.

Lean and obese male Zucker fatty/spontaneously hypertensive heart failure F1 hybrid (ZSF1-$Lepr^{fa}$ $Lepr^{cp}$/Crl) rats (Charles River, Kingston, NY, USA) were housed under a 12:12 h light–dark cycle (lights on at 6 a.m.) with *ad libitum* access to a standard high-glucose, high-protein diet for this animal model (PMI 5008 mod., Ssniff, Soest, Germany) and tap water during the whole study. For NASH development, at 8 weeks of age the ZSF1 obese rats (n=8) were switched to a modified 0% choline / 0.2% methionine diet (mMCD, TD.90262.mod, S8146-E025, Ssniff, Soest, Germany) for 17 weeks. Subsequently, during the last 9 weeks the MCD diet was combined with oral administration of carbon tetrachloride (CCl_4, 200 mg/Kg in olive oil, twice per week) (Sigma Aldrich, Germany). This group was compared with the healthy lean ZSF1 (fa/- (cp)) (n=8, 34-36 weeks old) and the obese diabetic ZSF1 (fa/fa (cp)) (n=8, 34-36 weeks old) control groups. All experiments were approved by the Ethics Committee of the State Ministry of Agriculture, Nutrition and Forestry (State of Hessen, Germany).

16. Characterization of the NASH animal model.

Body weight, blood and serum parameters. Body weight was monitored with a scale (Mettler Toledo, Modell New Classic MF, Switzerland and Kern PCB 1600, Kern & Sohn GmbH, Germany) and blood glucose was measured with a glucometer (ACCU-CHEK Aviva, Roche Mannheim Germany, Model:NC).

Rats were euthanized under isoflurane by aorta exsanguination. Blood was collected (S-Monovette Z-Gel, clot activator, Sarstedt) and let stand for 20 minutes at room temperature and centrifuged (4°C, 10 minutes, 4000 rpm) for serum collection. Serum safety liver enzymes alanine transaminase (ALT) and aspartate aminotransferase (AST) were measured in singles (Cobas 6000 with the c- Modul von Roche Diagnostics). Serum insulin was measured with an ELISA kit (Mercodia Rat/Mouse Insulin FIA No. 10-1248-10) in singles following manufacturer guidelines.

Liver triglycerides and cholesterol. 0.5 g of liver were placed in 5 ml of a dichloromethane / methanol mixture at a ratio of 2:1 and left for 2-3 days in a closed glass tube. On day 1, 2 ml of distilled water were added, and the samples centrifuged for 20 minutes at 3000 rpm. The upper aqueous phase was discarded, and the lower dichloromethane phase transferred into a graduated centrifuge tube where further 5 ml dichloromethane / methanol were added and left for 15 minutes. The samples were sonicated, 2 further ml of distilled water added and again centrifuged for 20 minutes at 3000 rpm. After discarding the supernatant, a constant volume of dichloromethane was added, and 1 ml transferred to an Eppendorf Cup safe-lock and allowed to evaporate for 24 hours or with the help of a water bath at 60 °C until the dichloromethane was gone. On day 2, 1 ml of 2-propanol was added, and the samples heated in a water bath at 60 °C for 30 minutes. Afterwards the samples were vortexed and centrifugated at 14000 rpm for 3-5 minutes. Triglycerides and cholesterol determination was done with Beckman Coulter AU680.

Liver glycogen: 1 g of liver was placed in 5 ml 0.6 molar perchloric acid and homogenized with Ultra-Turrax with the samples always kept on ice. Glycogen content was determined with Beckman Coulter AU680.

Histology and pathological analysis. According to the study plan the organs were sampled and fixed in 4% neutral buffered Formaldehyde for 24h. The liver trimming was done on the area of interest from each sample. Afterwards the dehydration of the tissue was done automatically by a VIP5 (Sacura) in approx. 20 h, followed by the blocking procedure. Slide section, 4 µm thick, were taken from each sample and dried overnight on a stretching table with 45°C.

The following stainings were done automatically: H&E and Sirius red used by a Gemini (Thermo Fisher).

H&E

Deparaffination in xylene 3 times for 3 min.; rehydration in Ethanol: 100 %, 95 % and 70 %, 1 min. each; Staining of the nuclei with Haemalum from Mayer within 5 min., rinsing with tap water for 10 min.; ascending ethanol 70 and 99%. Staining of tissue with Eosin for 30 sec.; 3x ethanol and xylene for dehydration and permanent covering at the end covering.

Result: nuclei: blue; tissue: red

Sirius red

Deparaffination in xylene 3 times for 3 min.; rehydration in Ethanol: 100 %, 95 % and 70 %, 1 min. each; Staining of the nuclei with haemalum from Weigerts within 8 min., rinsing with tap water for 10 min. Staining of tissue with pikro- sirius red for 30 sec.; differentiation 2 x with acetic acid 30%. 3x ethanol and xylene for dehydration and permanent covering at the end covering

Result: nuclei: black brown; tissue. yellow and fibrotic tissue: red

PAS

Deparaffination in xylene 3 times for 3 min.; rehydration in Ethanol: 100 %, 95 % and 70 %, 1 min. each; pickle the slides with periodic acid for 10 min., rinsing with tap water for 1 min Staining of glycoproteins with schiff's reagent within 10 min., rinsing with tap water for 1 min. Staining of the nuclei with haemalum for 2 min.; rinsing with tap water for 1 min 3x ethanol and xylene for dehydration and permanent covering at the end covering.

Result: nuclei: dark blue; glycoproteins pink

ED1

Deparaffination in xylene 3 times for 3 min.; rehydration in Ethanol: 100 %, 95 % and 70 % and aqua dest, 1 min. each. For blocking of the endogenous peroxidase 3% H_2O_2 for 20 min., rinsing with aqua dest for 1 min. For the renaturations citrate buffer pH 6.0 for 20 min. at 98 °C und 1 bar was taken, for blocking of the unspecifity the blocking reagent UV block was incubated for 5 min. Incubation of the primary ab with CD 68 (AbD) clone ED1 dilution 1:100 within 60 min. The further reagents were taken from the Ultra Vision LP/ HRP Polymer Kit (Thermo). First step from the kit are the ab enhancement for HRP (horseradish phosportase) for 10 min., following from HRP Polymer for 15 min. and the chromogen DAB for 3 min., between all steps a rinsing with tris buffer was done. The counterstaining follows at the end with haemalum for 2 min. 3x ethanol and xylene for dehydration and permanent covering at the end covering.

Result: macrophages dark brown, Tissue and nuclei: blue

17. *In vivo PET imaging.*

The rats were anesthetized with isoflurane-oxygen-nitric oxide mix with 4% isoflurane in at start, and 1,5-2% as maintenance dose. A tail vein catheter was inserted under anesthesia and the animal was placed on Siemens INVEON small animal PET/CT scanner (Siemens Medical Solutions, Erlangen, Germany). The animals were kept warm with a warming plate and body temperature and breathing was monitored over the course of the scan (BioVet CT1, m2m Imaging Corp. USA)

Each animal was dosed with single i.v. bolus of 5±0.5 MBq [^{68}Ga]-GN probes with a maximum volume of 200 µL. Dynamic PET acquisition was performed for 30 min or 150 min, followed by CT for attenuation correction. The animals were warmed and left to recover in their cages and were not used again in the studies earlier than 1 week.

The decay, attenuation and scatter corrected dataset was histogrammed to 23 frames (6 x 10 sec, 14 x 1 min, 3 x 5 min) for the 30min protocol and 21 frames (6 x 10 sec, 4 x 1 min, 2 x 5 min, 9 x 15 min) for the 150min protocol, and reconstructed using OSEM 3D algorithm. The matrix size of the reconstructed PET images was 128x128x159 with reconstructed pixel size 0.078 mm. The CT attenuation parameters were step and shoot, 121 projections, angle 0 to 200, 80 kVp, 500µA and Feldcamp reconstructed matrix size of 512 x 512 x 680 and pixel size of 0.195 mm.

Blocking studies were conducted as above, but various concentrations (4 mg/mL in PBS pH 7.4) of blocker (add compound) were injected 30 sec before tracer administration in the anesthetized SD rodents. Chasing studies were conducted as above, but 4 mg/kg (in PBS pH 7.4, 100 µL) single i.v. doses of blocker were injected 2 min, 5 min and 15 min after tracer administration in the anesthetized SD rodents via the tail vain catheter while the PET acquisition.

In vivo PET imaging of the ZSF1 animals was performed using ^{68}Ga labelled GN-01 as described before. The ZSF1 lean animals were imaged only once at timepoint 35-36 weeks. The ZSF1 obese animals were imaged at timepoint 24 weeks and again at timepoint 35-36 weeks. The obese ZSF1 (mMCD/CCl_4) animals were imaged at the start of mMCD diet (10 w), at start of CCl4 treatment (28 w) and on three following timepoints (week 31, 33 and 35-36).

18. PET image analysis.

PET image analysis was performed by the Siemens Inveon Research Workplace software (Siemens Medical Solutions). Regions of interest (ROI) from selected tissues of interest were

drawn manually on co-registered PET/CT images. In addition, total liver volume was calculated from the PET images using a summed image thresholding, and weight was estimated (1cc=1g). The radioactive uptake within the ROI was expressed as percentage injected dose per gram (% ID/g) calculated from the decay corrected radioactivity per area (1cc= 1g) within the ROI, and from the total injected dose. Time-activity curves were subsequently plotted from the dynamic images and ROI % ID/g. Data was further presented as Standardised Uptake Value (SUV) which normalises the %ID/g to body weight, and as total liver activity (%ID/g x image derived liver volume in grams) in order to take into account the changes in body mass and liver volume between the groups.

19. PCR and Western blot.

The expression of the genes encoding asialoglycoprotein receptors 1 and 2 (gene names Asgr1 and Asgr2) was quantified applying two-step droplet digital Polymerase Chain Reaction (ddPCR) as described.[29] The iScript™ cDNA synthesis kit (Bio-Rad GmbH, München, Germany) was used for the cDNA generation according to the manufacturer's instructions in a T100 thermal cycler (Bio-Rad). The ddPCR™ Supermix for probes (no dUTP, Bio-Rad) was used for the ddPCR reaction. After droplet generation with the Automatic Droplet Generator (Bio-Rad), thermal cycling took place followed by reading of the droplets on a QX200 Droplet Reader (Bio-Rad).

The following primers and probes were used:

Asgr1 (rat): Probe: ATTCCGTCTCTTCCTGCTTTCCCTG
Primer 1: CACAGACAACCACCAGCA
Primer 2: AGAACGACCACCATCAACTC

Asgr2 (rat): Probe: ACAACGAATTCCTGCTCCTCCCTG
Primer 1: GACCTATCCAAATGTGAAACGC
Primer 2: GTACTGCCAAATGGAGAATGC

Antibodies for Western Blotting were from Proteintech (Manchester, UK, ASGR1 rabbit polyclonal, cat. No. 11739-1-AP), ThermoFisher (Germany, ASGR2 rabbit polyclonal, cat. no. PA5-89547) and Abcam (Berlin, Germany, anti-beta actin mouse monoclonal, cat. no. ab8226).

Liver tissue was homogenized in 50 mM Tris-HCl, pH 7.5, 100 mM KCl, 5 mM $MgCl_2$, using the Precellys Lysing Kit (Bertin, Frankfurt, Germany, cat. no. KT03961-1-009.2). Protein concentration was determined using the Pierce BCA protein assay kit (ThermoFisher, Germany, cat. no. 23225). 50 µg protein per lane were separated on a 10 % Bis-Tris gel (ThermoFisher, cat. no. WG1203BX10) and blotted onto a nitrocellulose membrane using the iBlot system (ThermoFisher). Primary antibodies were used at 1:1000 dilution, secondary antibodies (Goat anti-rabbit-IRDye 680LT and Goat anti-mouse IRDye 800CW, both from LI-COR, Bad Homburg, Germany) were used at 1:15000 dilution. Band intensities were analyzed on a LI-COR Odyssey Imaging System.

20. Statistical analysis.

All data used for statistical evaluations (GraphPad Prism software) were checked for normality of the distributions by the Shapiro-Wilk and Kolmogorov-Smirnov tests. Nonparametric statistics were used when the assumption of normality was violated. All values are expressed as mean ± SEM. The statistical analysis has been done with one-way ANOVA or Kruskal-Wallis test followed with multiple comparisons.

The Spearman rank-order correlation was used to assess the relationships between two variables. When parametric statistics were applied, the t test for paired samples was used for comparison of repeated measures. $P \leq 0.05$ (two-tailed) were considered significant.

[^{68}Ga] GN-01

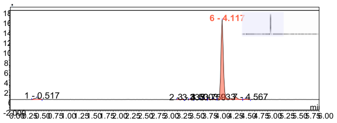

[^{68}Ga] GN-03

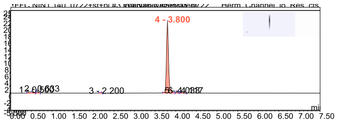

[^{68}Ga] GN-05

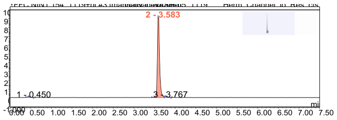

Figure S1. Representative radioHPLC chromatogram, with the peak in red the product.

A) **[^{68}Ga] GN-01**. ***B)*** **[^{68}Ga] GN-03**. ***C)*** **[^{68}Ga] GN-05**

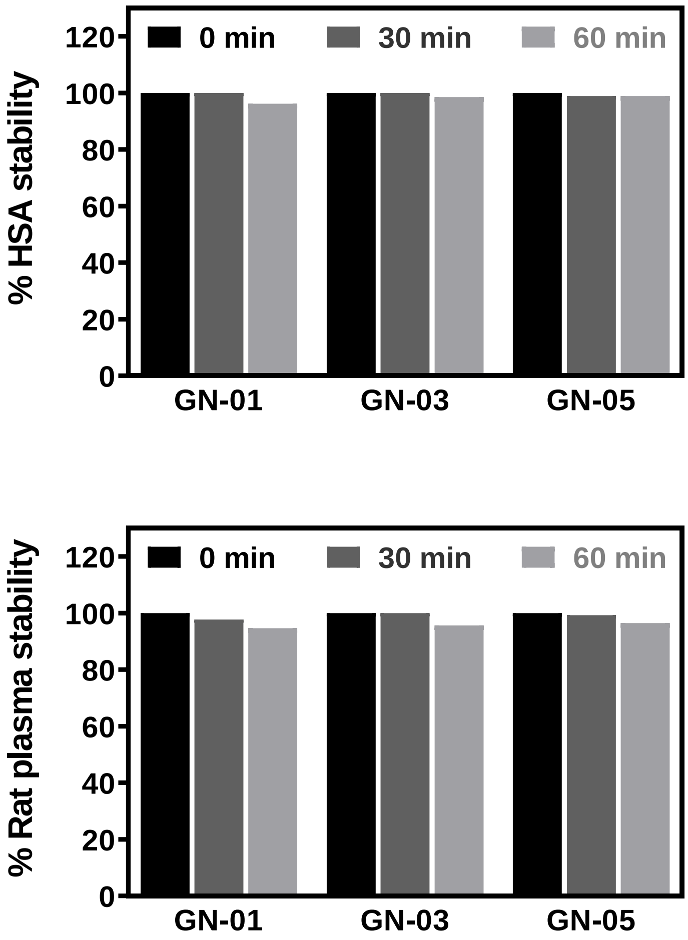

Figure S2: HSA and rat plasma studies of [^{68}Ga] - (GN-01, GN-03, GN-05) evaluated by radioHPLC chromatogram.

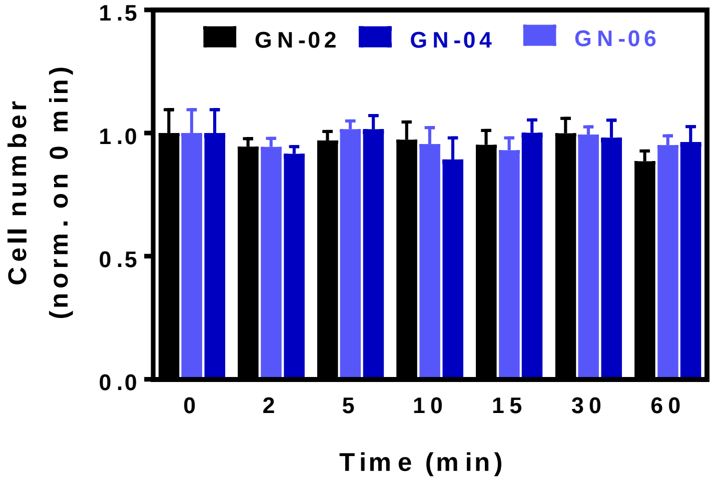

Figure S3: Cell viability assay. Cy5.5 labelled probes (GN-02, GN-04, GN-06) have no effect on the viability of HepG2 cells in the assay window. Concentration of probes 1 µM (n=4), mean ± S.D.

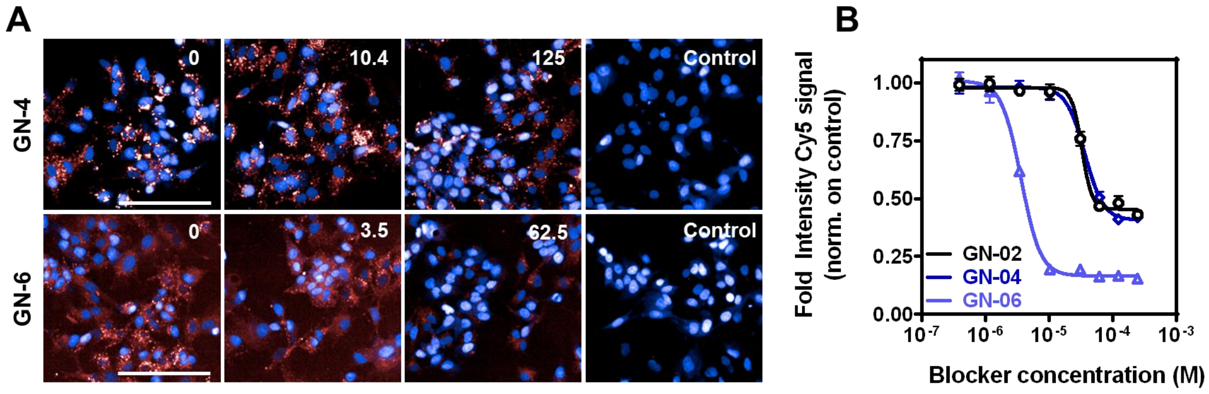

Figure S4: *In vitro* characterization of Cy5.5 labelled probes (GN-02, GN-04, GN-06) on HepG2 cells. Blocking experiments were performed upon pre-incubation of blocker (compound 28 = 0, 0.4, 1.2, 3.5, 10.4, 31.3, 62.5, 125 and 250 µM concentrations) on HepG2 cells for 5 minutes and then addition of 1 µM GN-02, GN-04, GN-06 for 30 minutes. **A)** Representative confocal images of GN-04 (upper) and 06 (lower panel) probes blocked by different concentrations blocker in HepG2 cells, concentration in µM indicated in upper right corner of image, control without GalNAc-NASH probe, blue = nuclei, red = Cy5 labeled GalNAc-NASH probes. **B)** EC_{50} value determination of GalNaC-NASH probe internalization via Cy5 signal intensity normalized on high and low controls. Data represented as mean ± S.D. (n=4) and scale bars = 100 µm.

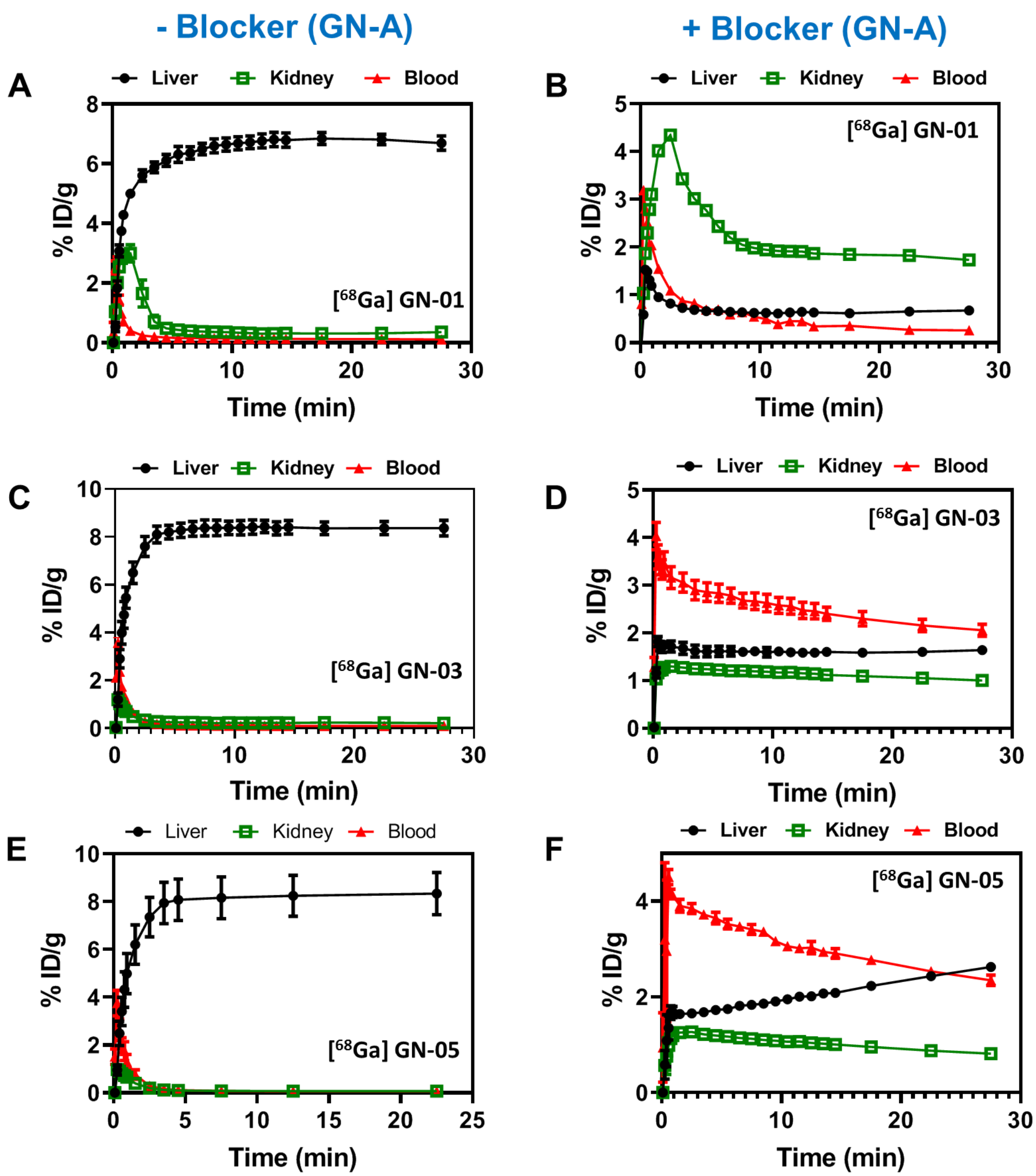

Figure S5: Comparative *in vivo* blocking studies of [^{68}Ga] - (GN-01, GN-03, GN-05) by PET imaging. ≈ 5 MBq dose of ^{68}Ga- GN probes were administered in SD rats [n (animals)= 3 per group]. Left panel is without blocker (GN-A) and Right panel is with 4mg/kg dose of blocker (GN-0). %IDg are shown with liver (black), kidney (green), blood (red). ***A)*** Time activity curve (TAC) of [^{68}Ga]-GN-01 without GN-0. ***B)*** TAC of [^{68}Ga]-GN-01 with GN-0. ***C)*** TAC of [^{68}Ga]-GN-03 without GN-0. ***D)*** TAC of [^{68}Ga]-GN-05 with GN-0. ***E)*** TAC of [^{68}Ga]-GN-05 without GN-0. ***F)*** TAC of [^{68}Ga]-GN-05 with GN-0. Data represented as mean ± S.E.M.

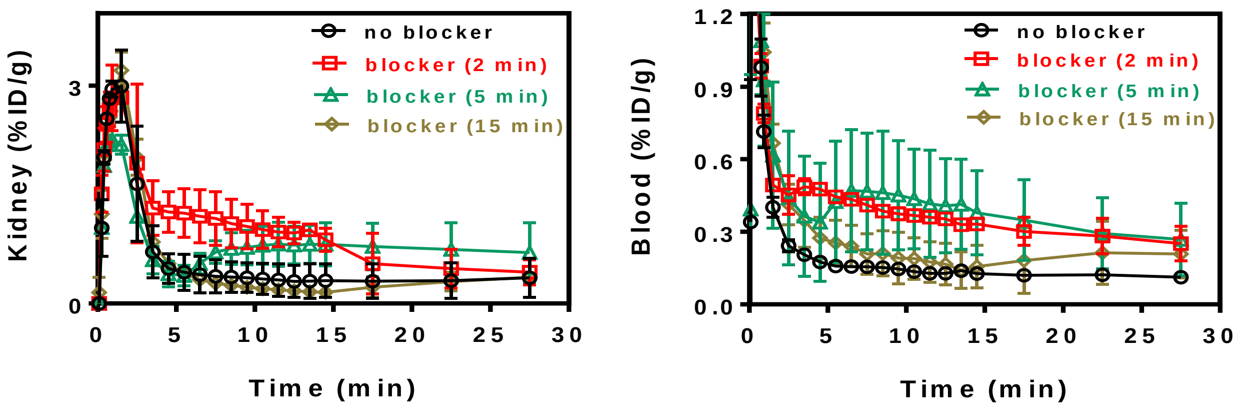

Figure S6: *In vivo* chasing studies of GN-01 in SD-Rats, kidney (left) and blood (right) activity (%ID/g) over time. [^{68}Ga]-GN-01 was injected (ca 5MBq) and second bolus of GN-A (4 mg/kg) was injected after 2 min (red), 5 min (green) and 15 min (brown). n(animals) = ≥ 3 per group, mean ± S.E.M.

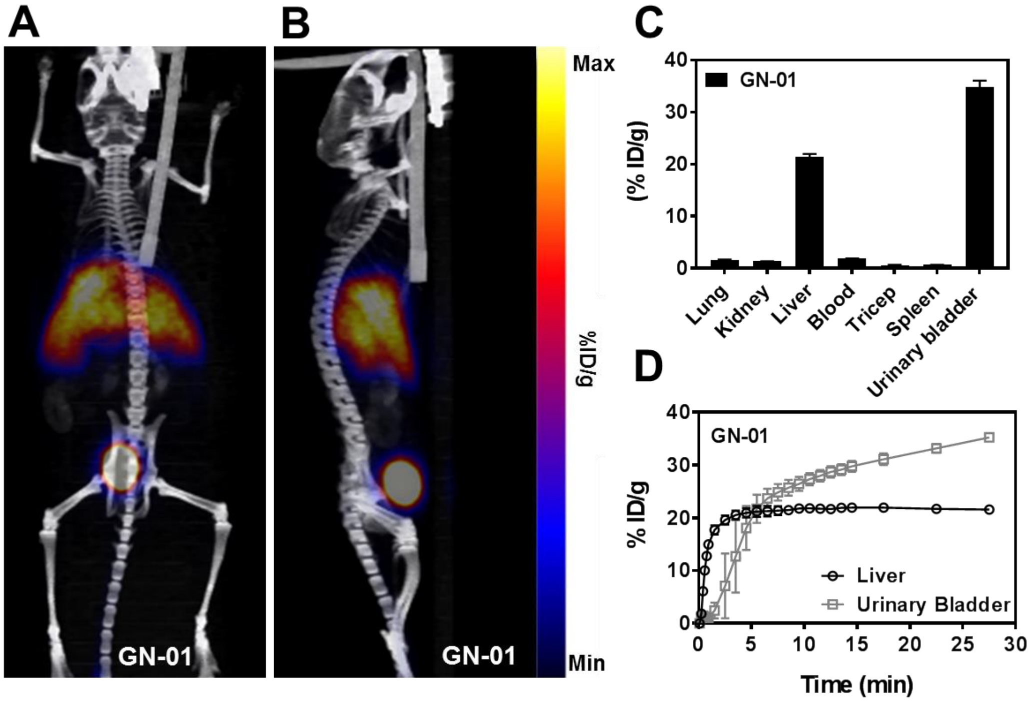

Figure S7: *In vivo* biodistribution of [^{68}Ga]-GN-01 in CD-1 mice. **A)** and **B)** Representative PET-CT images after 30 minutes upon ≈ 5 MBq dose of [^{68}Ga]-GN-01 administration in mice (n= 6). **C)** Image derived uptake of [^{68}Ga] GN-01 in tissues at 30 minutes. **D)** Time activity curve of [^{68}Ga]-GN-01 in liver and urine shows very rapid and high accumulation and retention in liver, and accumulation of activity in urine. Data represented as mean ± S.E.M. [n (animals)= 6].

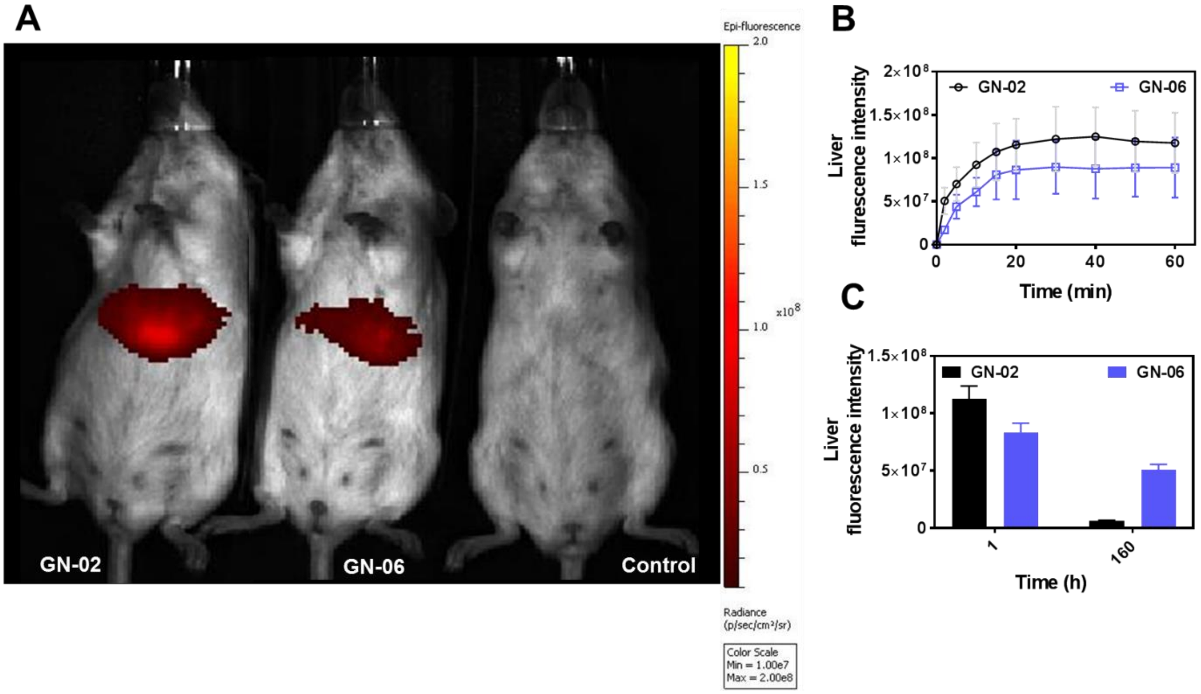

Figure S8: *In vivo* biodistribution studies of GN-02 and GN-06 in CD-1 mice via fluorescence imaging (IVIS). **A)** Representative background corrected fluorescence intensity images at 60 minutes of GN-02 and GN-06 administration (25µM of 50 µL) (i.v.) and a control mouse. **B)** Fluorescence intensity of GN-02 and GN-06 in liver at different time points. **C)** Retention of GN-02 and GN-06 in liver over time period time of one-week. Data represented as mean ± S.E.M. [n (animals)= 4 per group].

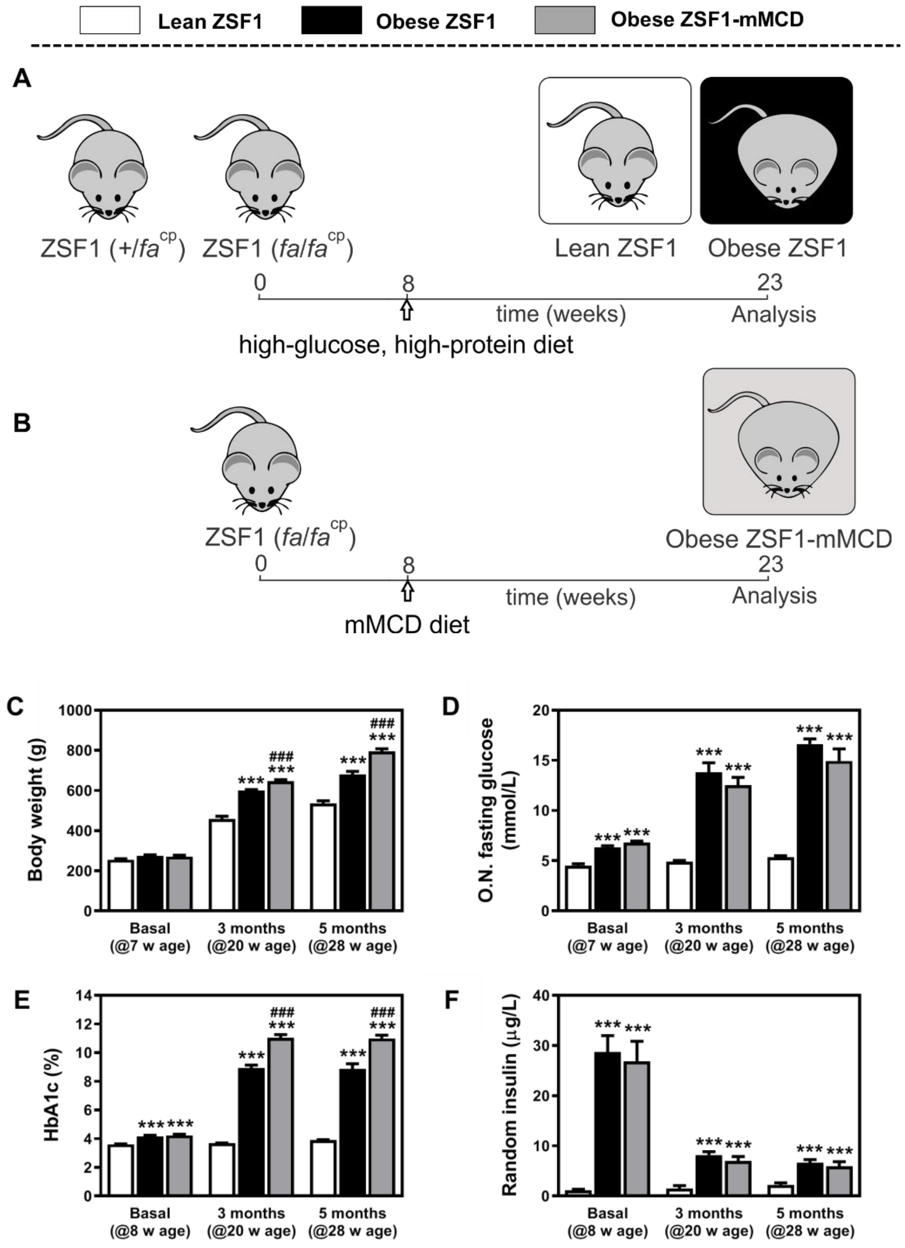

Figure S9: Obesity and diabetes. (A) ZSF1 (*fa*/*fa*^{cp}) and ZSF1 (+/*fa*^{cp}) animals served as controls. They were fed a standard high-glucose, high-protein diet but not exposed to hepatotoxin. The ZSF1 (*fa*/*fa*^{cp}) animals became obese, while the ZSF1 (+/*fa*^{cp}) animals remained lean (metabolically normal). **(B)** ZSF1 (*fa*/*fa*^{cp}) rats were fed a modified methionine/choline-deficient diet (0% choline, 0.2% methionine) for 23 weeks preserved the obese **(C)** and diabetic phenotype **(D-F)** observed in the obese ZSF1 group. Data represented as mean ± S.E.M. One-

way ANOVA statistical analysis ***p<0.001 vs the lean ZSF1 group, ###p<0.001 vs the obese ZSF1 group [n (animals) = 4-6].

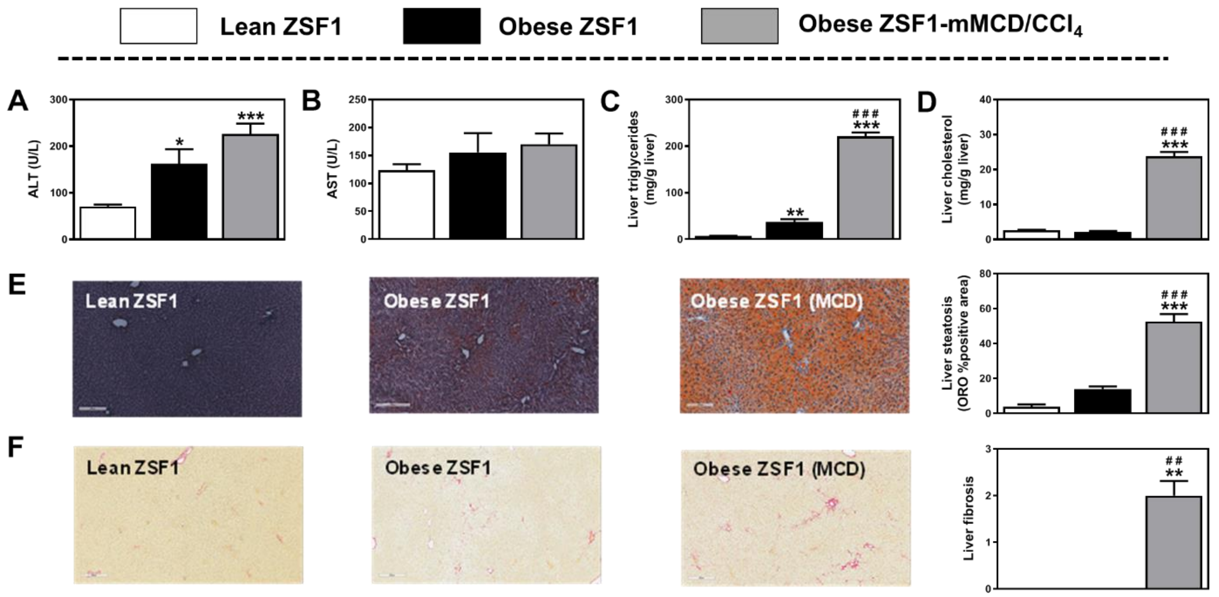

Figure S10: Liver NAFLD/NASH. The consumption of 0% Cho / 0.2% Met diet (mMCD) for 23 weeks led to high ALT (A) and no changes in AST (B) serum levels, like the obese ZSF1 group. However, liver triglycerides (C) and cholesterol (D) content was higher in the ZSF1 mMCD group even when compared with the ZSF1 obese group. Histological analysis shows no difference in liver steatosis (E) between the obese groups but higher fibrosis (F) with mMCD diet. Data represented as mean ± S.E.M. One-way ANOVA statistical analysis *p<0.05, ***p<0.001 vs the lean ZSF1 group, ###p<0.001 vs the obese ZSF1 group [n(animals) = 5-6].

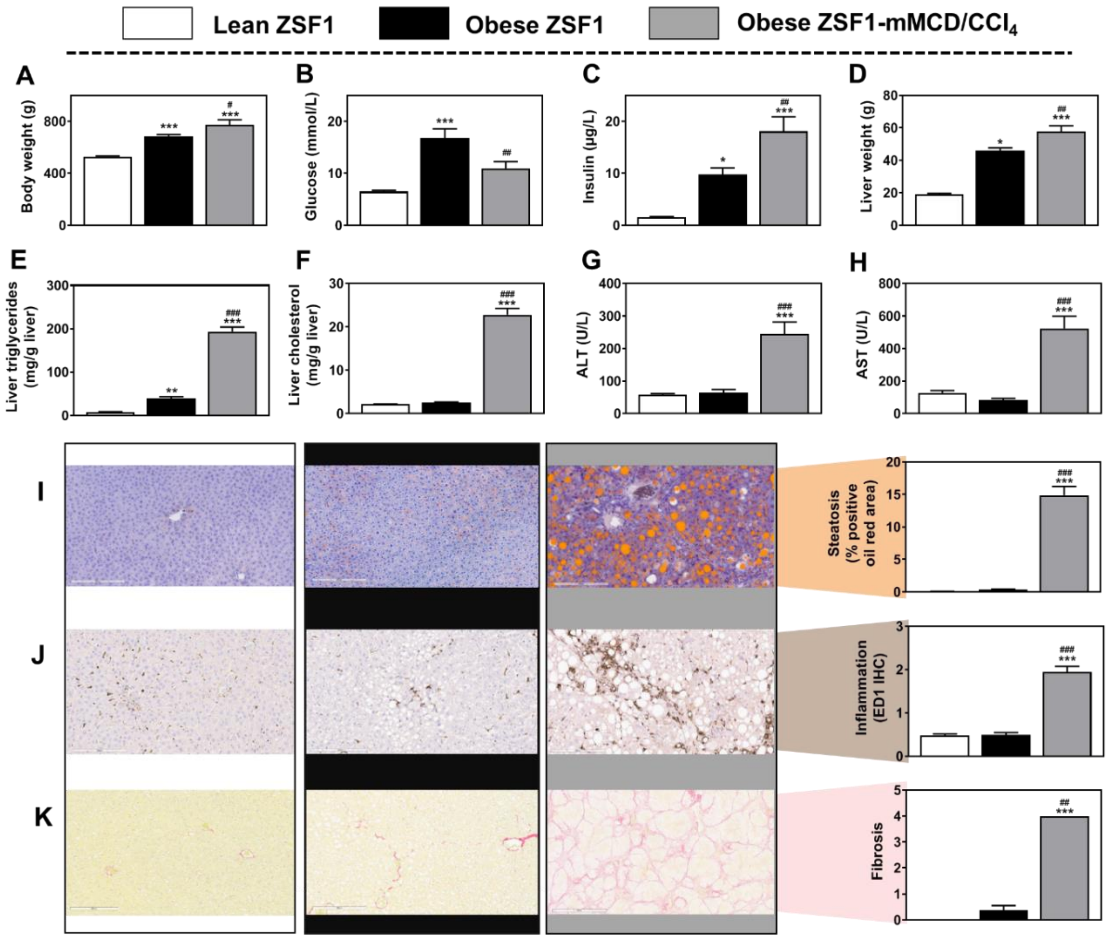

Figure 11: The ZSF1 rat as an obese, diabetic model of NASH. ZSF1 rats consuming a modified methionine/choline-deficient diet (0% choline, 0.2% methionine) for 17 weeks together with CCl_4 administration for 9 weeks (mMCD/CCl_4) retained the obese, insulin-resistant phenotype of the obese ZSF1 rats based on **(A)** high body weight, **(B)** high glucose trend **(p=0.056)** and **(C)** high insulin. **(D)** The obese ZSF1-mMCD/CCl_4 rats also showed enlarged liver in relation to **(E)** higher triglycerides and **(F)** cholesterol levels. These results together with higher serum liver transaminases **(G)** ALT and **(H)** AST, than the livers of the lean and obese ZSF1 groups indicated liver damage. Histopathology of liver tissue showed greater NASH-like **(I)** steatosis (based on oil red O staining), **(J)** inflammation (based on immunohistochemistry against ED1) and **(K)** fibrosis (based on Sirius Red staining) in ZSF1-mMCD/CCL_4 rats than in the other two groups. 200 µm scale bar in each IHC pictures. Data shown are mean ± S.E.M. Differences were analyzed using one-way ANOVA and Kruskal-Wallis test. *p<0.05, *p<0.01, ***p<0.001 vs. the lean ZSF1 group; $^{\#\#}$p<0.01, $^{\#\#\#}$p<0.001 vs. the ZSF1 obese group (n=7-8).

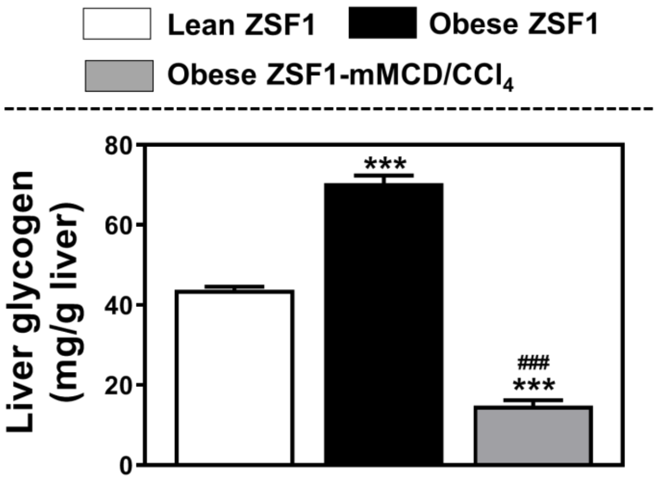

Figure S12: Liver glycogen. The combination of 0% Cho / 0.2% Met diet consumption and CCl_4 administration (mMCD/CCl_4) led to lower glycogen content compared with the lean and the obese ZSF1 groups. Data represented as mean ± S.E.M. One-way ANOVA statistical analysis ***p<0.001 vs the lean ZSF1 group, ###p<0.001 vs the obese ZSF1 group [n(animals) = 8].

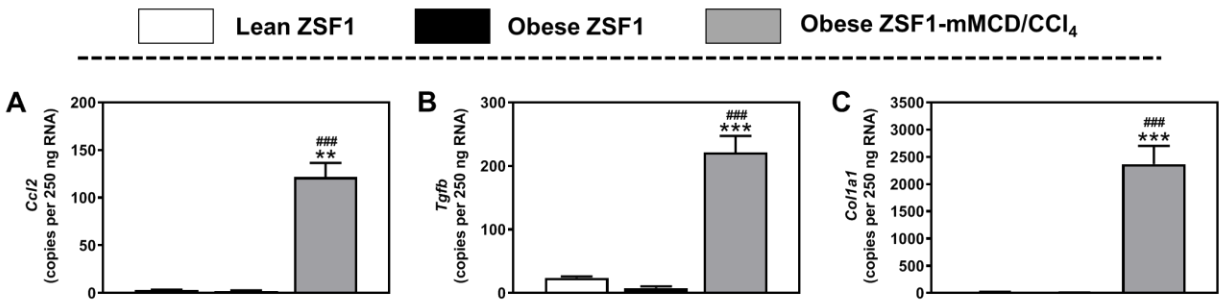

Figure S13: Liver NAFLD/NASH – gene expression. The combination of 0% Cho / 0.2% Met diet consumption and CCl_4 administration (mMCD/CCl_4) led to higher *Ccl2* (A), *Tgfb* (B) and *Col1a1* (C) liver expression compared with the lean and obese ZSF1 groups. Data represented as mean ± S.E.M. One-way ANOVA statistical analysis **p<0.01, ***p<0.001 vs the lean ZSF1 group, ###p<0.001 vs the obese ZSF1 group [n(animals) = 8].

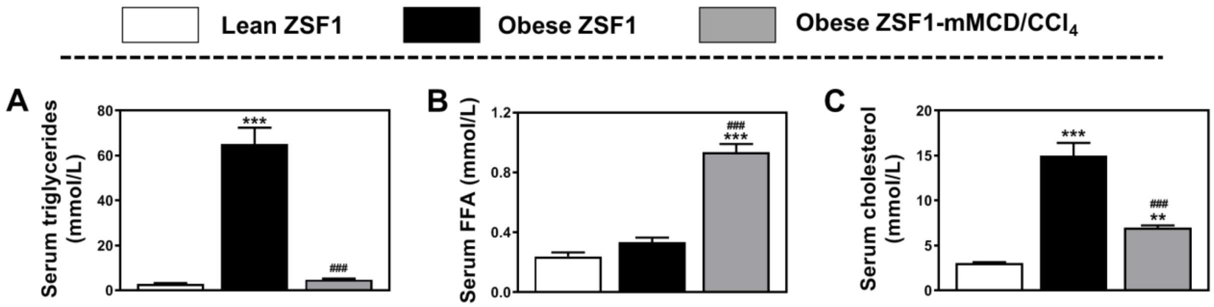

Figure S14: Serum lipids. The combination of 0% Cho / 0.2% Met diet consumption and CCl_4 administration (mMCD/CCl4) led to lower triglycerides (A) compared with the obese ZSF1 group and higher free fatty acids (FFA) (B) compared with the lean and obese ZSF1 groups. The obese ZSF1-mMCD/CCl_4 group presented higher cholesterol compared with the lean ZSF1 group but lower than the obese ZSF1 group (C). Data represented as mean ± S.E.M. One-way ANOVA statistical analysis **p<0.01, ***p<0.001 vs the lean ZSF1 group, ###p<0.001 vs the obese ZSF1 group [n(animals) = 8).

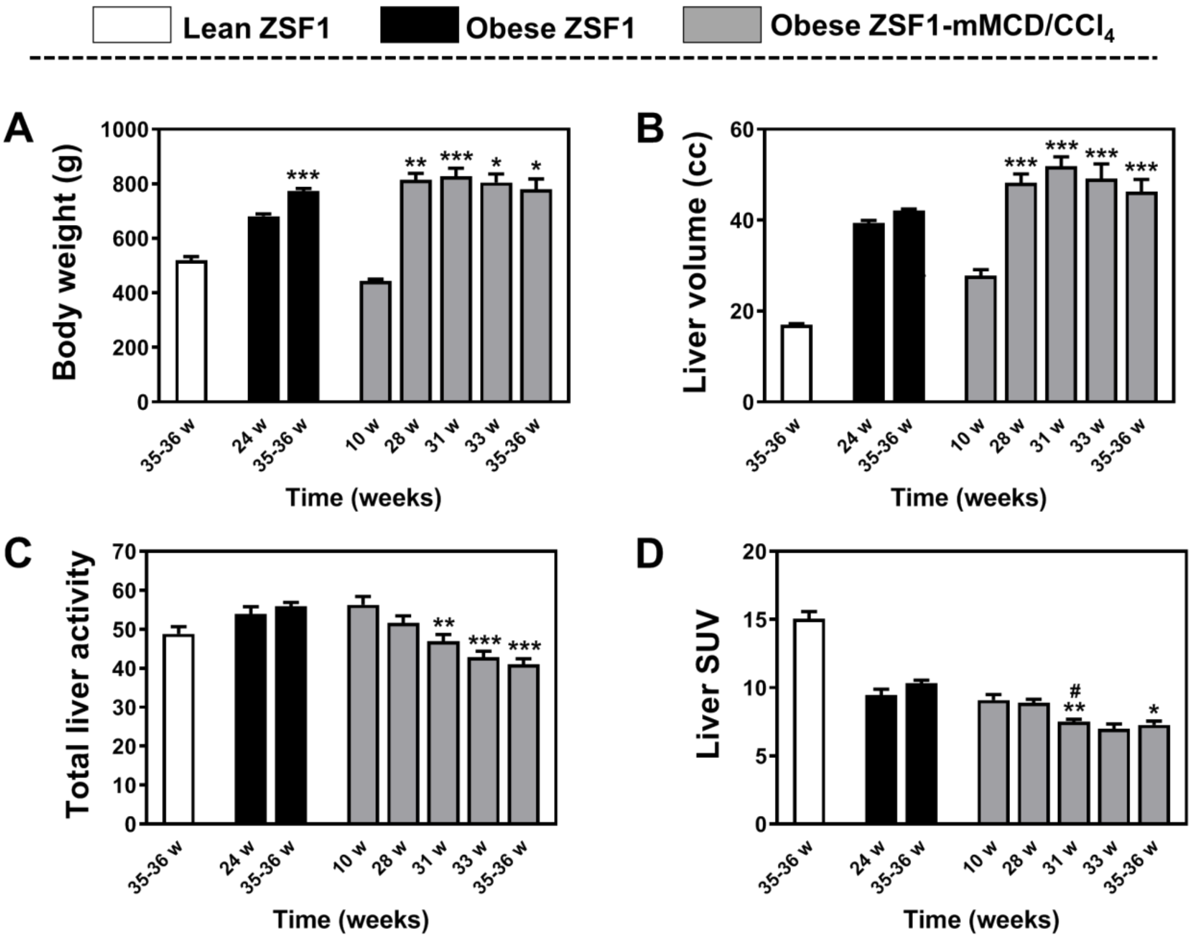

Figure S15: Comparative analysis for NASH time course. A) Graph represents body weight of each animals at different age. Although liver volume of the obese ZSF1-mMCD/CCl_4 group does not show differences with aging from 28 weeks on (B), PET analysis with the [^{68}Ga]-GN-01 shows a decrease of signal parallel to the progression of NASH (C-D). Data represented as mean ± S.E.M. One-way ANOVA and unpaired t-test statistical analysis *p<0.05, **p<0.01, ***p<0.001 vs the initial time point for the corresponding group, #p<0.05 vs the second time point for the corresponding group [n (animals) = 5-8 per group].

Table S3. [6-12]

Publication	Animal species and strain	Sex	Diet	Time on diet (weeks)	Hepatotoxin	Time on hepatotoxin (weeks)	NASH				Obesity	GI/ IR/Diabetes
							Steatosis	Inflammation	Ballooning	Fibrosis		
Yamaguchi K. et al., *Hepatology* 2007	BKS.Cg-m+/+Lep^{db}/J mouse	Male	MCD diet	8	--	--	M	Yes (NS)	ND	Yes (NS)	No	No
Kristiansen M.N. et al., *WJH* 2016	C57BL/6J DIO mouse	Male	AMLN diet	26	--	--	S	M	No	L	Yes	IR
	Lep^{ob}/Lep^{ob} mouse	Male	AMLN diet	12	--	--	M	M	L	S	Yes	Improvement vs Lep^{ob}/Lep^{ob} control
Asgharpour A. et al., *J. Hepatol.* 2016	DIAMOND mouse (C57BL/6Jx129S1/SvImJ)	Male	WD + fructose-glucose water	16-24	---	---	S	L	L	M	Yes	IR
Tsuchida T. et al., *J. Hepatol.* 2018	C57BL/6J mouse	Male	WD + fructose water	12	CCl_4	12 (320 mg/kg 1x)	S	S	M	S	No	No
Boland M.L. et al., *WJH* 2019	C57BL/6J DIO mouse	Male	GAN diet	28	--	--	S	M	No	M	Yes	ND
	C57BL/6J DIO mouse	Male	AMLN diet	28	--	--	S	M	No	M	Yes	ND
	Lep^{ob}/Lep^{ob} mouse	Male	GAN diet	16	--	--	S	M	No-L	M	Yes	GI
	Lep^{ob}/Lep^{ob} mouse	Male	AMLN diet	16	--	--	S	L-M	No-L	M	Yes	No
Sun G. et al., *BMC Gastroenterol.* 2019	FATZO (MS-NASH) mouse (AKR/JxC57BL/6J)	Male	WD + fructose water	20	--	--	S	L	L	L	Yes	ND
Gapp B., Jourdain M. et al., *Hepatol. Commun.* 2020	C57BL/6J DIO mouse	Male	HFD + fructose & sucrose water	20	--	--	S	S	ND	M	Yes	GI / no IR
Tølbøl K.S. et al., *Dig Dis Sci.* 2019	Wistar rat	Male	CD + cholesterol diet	12	--	--	M	M	L	ND	No	ND
Present study	ZSF1-$Lepr^{fa}Lepr^{cp}$/Crl rat (ZDF female x SHHF male)	Male	Modified MCD diet	26	CCl_4	9 (200 mg/Kg 2x)	S	M	ND	S	Yes	IR

Scoring:

0: No; 1: Low (L); 2: Moderate (M); 3: Severe (S)

AMLN: Amylin diet; CD: Choline diet; GAN: Gubra amylin NASH diet; GI: Glucose intolerance; HFD: High fat diet; IR: Insulin resistance; MCD: Methionine/choline-deficient diet; ND: Not determined; NS: No score; WD: Western diet.

References

1. Meier, M., et al., Crystal Structure of the Carbohydrate Recognition Domain of the H1 Subunit of the Asialoglycoprotein Receptor. *J. Mol. Bio.*, **300(4)**, 857-865 (2000).
2. Mayer M. et al. Characterization of ligand binding by saturation transfer difference NMR spectroscopy. Angew Chem Int Ed. 38,1784–1788 (1999).
3. Bhattacharya, A.A. et al. Crystallographic analysis reveals common modes of binding of medium and long-chain fatty acids to human serum albumin1. *J. Mol. Bio.*, **303(5)**, 721-732 (2000).
4. Krenzel et al. Correspondence of Fatty Acid and Drug Binding Sites on Human Serum Albumin: A Two-Dimensional Nuclear Magnetic Resonance Study. *Biochemistry* **52**, 1559−1567 (2013)
5. Hamilton, J.A. NMR reveals molecular interactions and dynamics of fatty acid binding to albumin. *Biochimica et Biophysica Acta* **1830(12)**, 5418−5426 (2013).
6. Yamaguchi K. et al. Inhibiting triglyceride synthesis improves hepatic steatosis but exacerbates liver damage and fibrosis in obese mice with nonalcoholic steatohepatitis. Hepatology **45(6),** 1366–1374 (2007).
7. Kristiansen M.N. et al., Obese diet-induced mouse models of nonalcoholic steatohepatitis-tracking disease by liver biopsy. World J. Hepatol. **8(16),** 673–684 (2016).
8. Tsuchida T. et al. A simple diet- and chemical-induced murine NASH model with rapid progression of steatohepatitis, fibrosis and liver cancer. J. Hepatol. **69(2),** 385–395 (2018).
9. Boland M.L. et al. Towards a standard diet-induced and biopsy-confirmed mouse model of non-alcoholic steatohepatitis: Impact of dietary fat source. World J Gastroenterol. **25(33),** 4904–4920 (2019).
10. Asgharpour A. et al., A diet-induced animal model of non-alcoholic fatty liver disease and hepatocellular cancer. J. Hepatol. **65(3),** 579-588 (2016).

11. Sun G. et al. The FATZO mouse, a next generation model of type 2 diabetes, develops NAFLD and NASH when fed a Western diet supplemented with fructose. BMC Gastroenterol. **19:41,** 1–11 (2019).

12. Tølbøl K.S. et al. Disease Progression and Pharmacological Intervention in a Nutrient-Deficient Rat Model of Nonalcoholic Steatohepatitis. Dig. Dis. Sci. **64**, 1238–1256 (2019).

13. Su Z. et al. Longitudinal Changes in Measured Glomerular Filtration Rate, Renal Fibrosis and Biomarkers in a Rat Model of Type 2 Diabetic Nephropathy. *Am J Nephrol.* **44(5)**, 339–353 (2016).

14. Dower K. et al. High resolution molecular and histological analysis of renal disease progression in ZSF1 fa/faCP rats, a model of type 2 diabetic nephropathy. *PLOS one* **23**, 1–23 (2017).

15. Yamaguchi K. et al. Inhibiting triglyceride synthesis improves hepatic steatosis but exacerbates liver damage and fibrosis in obese mice with nonalcoholic steatohepatitis. *Hepatology* **45(6)**, 1366–1374 (2007).

16. Kristiansen M.N. et al., Obese diet-induced mouse models of nonalcoholic steatohepatitis-tracking disease by liver biopsy. *World J. Hepatol.* **8(16),** 673–684 (2016).

17. Gapp B., Jourdain M. et al. Farnesoid X Receptor Agonism, Acetyl-Coenzyme A Carboxylase Inhibition, and Back Translation of Clinically Observed Endpoints of De Novo Lipogenesis in a Murine NASH Model. *Hepatol Commun* **4(1)**,109–125 (2019).

18. Simard, J. R., Zunszain, P. A., Ha, C.-E., Yang, J. S., Bhagavan, N.V., Petitpas, I., Curry, S., and Hamilton, J. A. *Proc. Natl. Acad. Sci. U.S.A.* **102**, 17958−17963 (2005).

19. Kabsch, W., XDS. *Acta Crystallographica Section D* **66(2)**, 125-132 (2010).

20. Evans, P., Scaling and assessment of data quality. *Acta Crystallographica Section D* **62(1)**, 72-82 (2006).

21. Tickle, I.J., Flensburg, C., Keller, P., Paciorek, W., Sharff, A., and C. Vonrhein, Bricogne, G., STARANISO., Global Phasing Ltd.: Cambridge, United Kingdom (2017).

22. Vonrhein, C., et al., Data processing and analysis with the autoPROC toolbox. *Acta Crystallographica Section D* **67(4)**, 293-302 (2011).
23. Emsley, P., et al., Features and development of Coot. *Acta Crystallographica Section D* **66(4)**, 486-501 (2010).
24. Bricogne G., B.E., Brandl M., Flensburg C., Keller P., Paciorek W., and S.A. Roversi P, Smart O.S., Vonrhein C., Womack T.O., Buster., Global Phasing Ltd: Cambridge, United Kingdom (2011).
25. Curry, S., et al., Crystal structure of human serum albumin complexed with fatty acid reveals an asymmetric distribution of binding sites. *Nature Structural Biology* **5**: 827 (1998).
26. McCoy, A.J., et al., Phaser crystallographic software. *Journal of Applied Crystallography* **40(4)**, 658-674 (2007).
27. Yang, F., et al., Effect of human serum albumin on drug metabolism: Structural evidence of esterase activity of human serum albumin. *Journal of Structural Biology* **157(2)**, 348-355 (2007).
28. Moerke N.J. et al., Fluorescence Polarization (FP) Assays for Monitoring Peptide-Protein or Nucleic Acid-Protein Binding. *Current Protocols in Chemical Biology* **1**, 1-15, (2009).
29. Kannt A. et al. Mastiha (Pistacia lentiscus) Improves Gut Microbiota Diversity, Hepatic Steatosis, and Disease Activity in a Biopsy-Confirmed Mouse Model of Advanced Non-Alcoholic Steatohepatitis and Fibrosis. *Mol Nutr Food Res.* **63(24)**, e1900927 (2019).
